# Projected impacts of climate change on functional diversity of frugivorous birds along a tropical elevational gradient

**DOI:** 10.1038/s41598-019-53409-6

**Published:** 2019-11-27

**Authors:** Irene M. A. Bender, W. Daniel Kissling, Katrin Böhning-Gaese, Isabell Hensen, Ingolf Kühn, Larissa Nowak, Till Töpfer, Thorsten Wiegand, D. Matthias Dehling, Matthias Schleuning

**Affiliations:** 1 German Centre for Integrative Biodiversity Research (iDiv) Halle-Jena-Leipzig, 04103 Leipzig, Germany; 2 Institute of Biology, Geobotany and Botanical Garden, Martin Luther University Halle-Wittenberg, 06108 Halle, Germany; 3 Senckenberg Biodiversity and Climate Research Centre (SBiK-F), 60325 Frankfurt am Main, Germany; 4 Institute for Biodiversity and Ecosystem Dynamics (IBED), University of Amsterdam, P.O. Box 94240, 1090 GE Amsterdam, the Netherlands; 5 Goethe University Frankfurt, Institute for Ecology, Evolution & Diversity, Biologicum, 60439 Frankfurt am Main, Germany; 6 Helmholtz Centre for Environmental Research - UFZ, Department Community Ecology, 06120 Halle, Germany; 7 Zoological Research Museum Alexander Koenig, Section Ornithology, 53113 Bonn, Germany; 8 Helmholtz Centre for Environmental Research - UFZ, Department Ecological Modelling, 04318 Leipzig, Germany; 9 Centre for Integrative Ecology, School of Biological Sciences, University of Canterbury, Private Bag 4800, Christchurch, 8140 New Zealand

**Keywords:** Biodiversity, Biogeography, Climate-change ecology, Ecological modelling, Ecological networks

## Abstract

Climate change forces many species to move their ranges to higher latitudes or elevations. Resulting immigration or emigration of species might lead to functional changes, *e*.*g*., in the trait distribution and composition of ecological assemblages. Here, we combined approaches from biogeography (species distribution models; SDMs) and community ecology (functional diversity) to investigate potential effects of climate-driven range changes on frugivorous bird assemblages along a 3000 m elevational gradient in the tropical Andes. We used SDMs to model current and projected future occurrence probabilities of frugivorous bird species from the lowlands to the tree line. SDM-derived probabilities of occurrence were combined with traits relevant for seed dispersal of fleshy-fruited plants to calculate functional dispersion (FDis; a measure of functional diversity) for current and future bird assemblages. Comparisons of FDis between current and projected future assemblages showed consistent results across four dispersal scenarios, five climate models and two representative concentration pathways. Projections indicated a decrease of FDis in the lowlands, an increase of FDis at lower mid-elevations and little changes at high elevations. This suggests that functional dispersion responds differently to global warming at different elevational levels, likely modifying avian seed dispersal functions and plant regeneration in forest ecosystems along tropical mountains.

## Introduction

Climate change is one of the main threats to biodiversity and its intensity is expected to increase in the future^1^. Species in tropical ecosystems are particularly sensitive to climate change, due to low climatic variability and high niche specialization of species^2,3^. Climate change is expected to be especially relevant for the biodiversity in tropical mountains because elevational gradients show high species turnover^4^ and steep temperature gradients, whereas latitudinal temperature gradients in the tropics are comparably shallow^5^. In response to changing climates, species are expected to alter their geographical and elevational ranges according to their climatic niche^5,6^. In mountainous areas, species likely shift their distribution upwards in response to increasing temperatures^5,7^. Although the direct and indirect effects of temperature on species’ elevational range limits in the tropics are not yet fully understood^8^, upward shifts in tropical mountains have already been observed for a variety of taxa, including insects, vertebrates and plants^9–12^. Yet, there is a lack of knowledge on how diversity patterns of entire ecological assemblages might change under climate change on tropical mountains.

Changes in biodiversity along elevational ranges might occur in a variety of ways^5^. When species at a given elevation shift their geographic ranges upwards in response to increasing temperatures, species from lower elevations that are already adapted to higher temperatures could move in and replace the emigrating species^5,13^. These range shifts might cause species turnover that could buffer changes in species richness at the given elevational levels^14^. At the lowest elevations in the tropical lowlands, however, upward range shifts and a lack of compensation by immigrating species might lead to a net loss of species richness, a process referred to as “lowland biotic attrition”^5,15^. At the highest elevations, species face a different challenge since they are not able to move their ranges beyond the mountain top and hence might go extinct^5,16,17^. In addition, other physical or biotic barriers (*e*.*g*., the tree line) could limit upward movements of species on mountains and cause species extinctions because these barriers might not necessarily shift upslope at the same pace^18^. Beyond these general theoretical expectations, little is known about the potential changes in the composition of ecological assemblages along mountains in response to climate change.

Taxonomic measures of biodiversity, such as species richness, do not necessarily reflect functional aspects of biodiversity, such as seed dispersal functions^19,20^. Functional aspects of biodiversity are influenced by the functional traits of species that are present in an assemblage. For instance, seed dispersal of fleshy-fruited plants is influenced by variation in beak size and wing shape of animal dispersers because these functional traits influence fruit-handling and foraging strategies of frugivorous animals^4,21–25^. Information about traits that affect specific ecosystem functions^26^ therefore allows to link interspecific trait variability to ecosystem functioning^19^, *e*.*g*., by looking at the range and distribution of relevant functional traits within an assemblage (*i*.*e*., functional diversity). Measures of functional diversity have been shown to be related to ecosystem functioning^19,27^, including functions performed by animals such as seed removal and pollination^28^.

A variety of metrics are available to quantify functional diversity^29,30^. We focus on functional dispersion (FDis) as a measure of functional diversity because it is independent of species richness and particularly suitable to estimate functional complementarity among interacting species in an assemblage^31,32^. FDis is defined as the mean distance of individual species to the centroid of all species in a multidimensional trait space^31^ and hence measures the dispersion of species traits within the trait space of the assemblage. To connect this measure of functional diversity with species distribution models, we applied a novel approach by weighting FDis with the SDM-derived probability of occurrence of each species in current and projected future assemblages. This SDM-weighted index measures FDis based on the likelihood that a functional role is realized in current and projected future assemblages. A reduced occurrence probability of species with traits located at the margin of the multi-dimensional trait space will thus lead to a reduction of FDis.

While most research on functional diversity has focused on local communities, there is an increasing interest to study functional diversity at the level of assemblages (*e*.*g*., grid cells or elevational levels)^33^ and across biogeographic extents^34^. This approach can provide insights into the geographic distribution of functional trait diversity across latitudinal and elevational gradients (*e*.*g*., refs. ^4,35^). By combining FDis with projections of species ranges under scenarios of future climate change^36^, we can gain insight into how functional aspects of assemblage structure and associated ecosystem functions might change in the future^14^. To date, such assessments are particularly rare for species-rich elevational gradients in the tropics, since accurate data on species distributions, local co-occurrence and functional traits are difficult to obtain.

Here, we combine species distribution models (SDMs) with an analysis of functional diversity to assess the potential impact of future temperature and precipitation change on frugivore assemblages along an elevational gradient in the tropical Andes of southeast Peru. We focus on frugivorous birds because they are of key importance for the functioning of tropical forests, due their important role as seed dispersers^37,38^, and because their functional traits related to foraging and seed dispersal (*e*.*g*., beak, wing and tarsus shape) are well known^4,21^. Our approach comprises three steps. First, we derived projected frugivore assemblages by using species-specific SDMs based on mean annual temperature and mean annual precipitation to model current and future occurrence probabilities of bird species on each of seven studied elevational levels. Second, SDM-derived occurrence probabilities were refined by four dispersal scenarios. These hypothetical scenarios included a *no range change* scenario and three range change scenarios (*range contraction*, *range expansion* and *range shift*) which assumed that birds need to shift their lower and/or upper elevational range upslope to track their temperature niche^6^. This is consistent with observed species responses to climate change in mountainous areas^39,40^. Third, to assess possible changes in functional diversity under climate change, we calculated FDis as a measure of functional diversity, for current and projected future assemblages.

**Figure 1:**
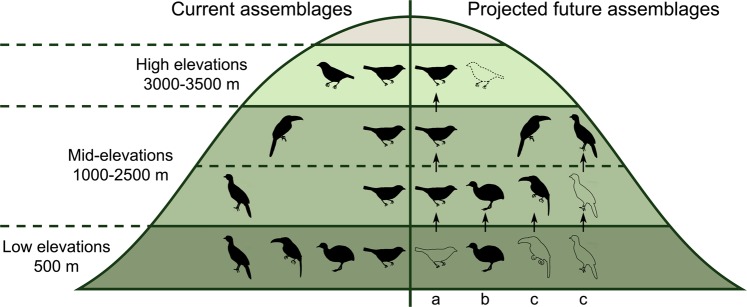
Current and potential future patterns in functional dispersion (FDis) of frugivorous bird assemblages along an elevational gradient in the Peruvian Andes. Current patterns in bird assemblages are shown on the left (“current assemblages”) and expected changes under future climate change are shown on the right side of the mountain (“projected future assemblages”). Currently, low elevations harbour the highest FDis, and FDis decreases with increasing elevation. In the future, projected changes could include (i) losses of FDis at the lowest elevations, due to the emigration of functional specialists, (ii) a rather constant FDis at mid-elevations if species immigrating and emigrating are functionally similar, and (iii) species extinction at high elevations, due to a dispersal barrier beyond the tree line. Ranges of species can be constant, contract (a), expand (b) or shift (c) in response to projected climate change; black bird silhouettes indicate the presence of a species on an elevational level, species outlines indicate that a species moved out of the respective elevational level, and the dotted silhouette indicates species extinction from the entire gradient. The entire gradient covers an elevational range from lowland (250 m) up to the mountaintop (3750 m). Forests cover the mountain up to 3500 m (green shading) and the studied gradient covers 500–3500 m of elevation.

We used the output from the climate-driven projection models to compare FDis values of current and future assemblages (Fig. 1) and to test the following hypotheses: (i) loss of FDis at the lowest elevation of the gradient, due to a decline in the number of functional specialists (*i*.*e*., species with a set of morphological traits that are distinct from the rest of the assemblage), (ii) little change in FDis at mid-elevations as the loss of emigrating species might be compensated by immigration of functionally similar species, and (iii) little change in FDis at high elevations because species extinctions might be functionally compensated by the immigration of species from mid-elevations. Our scenarios suggest that functional aspects of bird assemblages are likely to change differently in response to climate change at different elevations, potentially causing future changes in the distribution of functional diversity along tropical elevational gradients.

## Results

Our study included nearly all frugivorous bird species (*n* = 232, 95%) that currently occur along the elevational gradient of the Manú biosphere reserve and covered an elevational range from 500 m (lowlands) to 3500 m (tree line), at 500 m intervals (Supplementary Table S1). The highest number of species was found on the lowest elevational level (*n* = 139 at 500 m), followed by a continuous decrease of species richness towards higher elevations (*n* = 17 at 3500 m). Five species only occurred below 500 m and were therefore currently not represented in any of the seven analysed assemblages. A total of 52 species were restricted to the elevational level of the lowlands (*i*.*e*., 500 m), five species were restricted to the highlands (3000–3500 m, tree line), and no species occurred on all elevational levels. The species pool covered a wide range of trait values, *e*.*g*., body mass ranged from 7.2 g (the Slender-footed Tyrannulet *Zimmerius gracilipes*) to 2813 g (the Razor-billed Curassow *Mitu tuberosum*), and included species that differed widely in their foraging-related traits, *i*.*e*., bill width, bill length, bill height, tail length, tarsus length, tarsus sagittal width, tarsus lateral width, wing length, Kipp’s distance and body mass. Under current conditions, the highest value of FDis was found at 500 m and the lowest at 2500 m (Supplementary Fig. S1).

**Figure 2:**
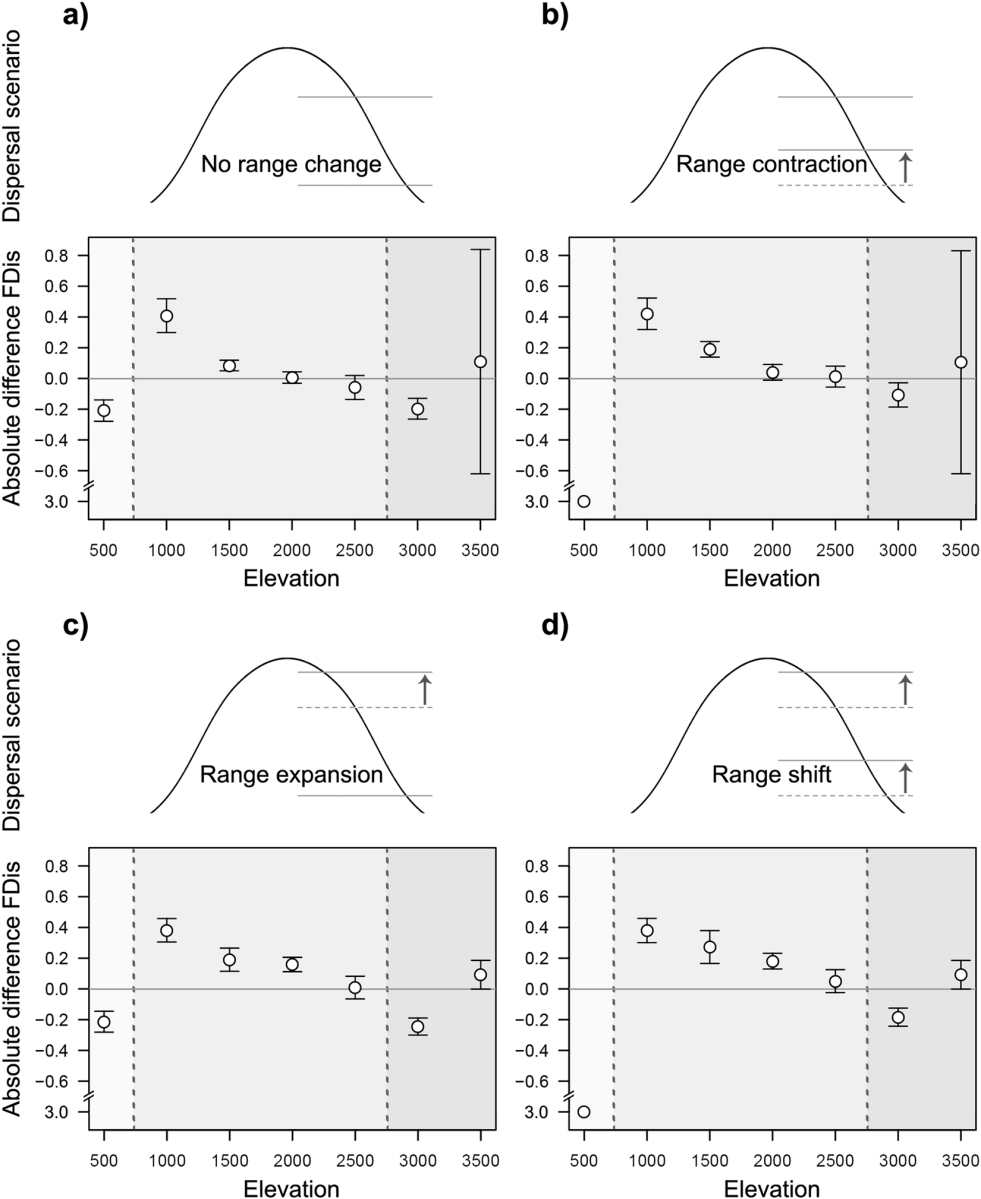
Potential changes in functional dispersion (FDis) of bird assemblages under projected future climate change (based on the RCP 8.5 scenario). Shown are four potential dispersal scenarios representing (**a**) *no range change*, (**b**) *range contraction* (lower range limit moves upwards), (**c**) *range expansion* (upper range limit moves upwards), and (**d**) *range shift* (both lower and upper range limit move upwards), with the lower panels showing the projected absolute changes in FDis, for each of seven elevational levels, representing a gradient from 500 m to 3500 m (at 500 m intervals). Absolute changes in FDis are based on species distribution models (SDMs) of 232 birds under current and projected future climate change scenarios using representative concentration pathway RCP 8.5 and the respective dispersal scenario. The values represent mean and standard deviation of the results derived from five general circulation models (CC, HE, MC, MG, NO). FDis calculations were derived from the first five PCoA axes, computed from ten morphological bird traits and are based on the SDM-derived probability of occurrence of bird species. Positive FDis values indicate an increase of FDis under climate change, and negative values a decrease. Projected future assemblages at 500 m were completely depleted of species under range contraction and shift scenarios.

Future climate was projected to change considerably along the elevational gradient under both considered representative concentration pathways (RCP, from the Fifth Assessment Report of the IPCC^41,42^), *i*.*e*., RCP 6.0 and RCP 8.5. Mean annual temperature and precipitation were projected to increase under both RCP 6.0 (ΔT = 2.3 ± 0.06 °C, ΔP = 136.9 ± 94.81 mm/yr) and RCP 8.5 (ΔT = 3.3 ± 0.08 °C, ΔP = 166.1 ± 95.71 mm/yr). Mean annual temperature and precipitation were used to fit SDMs to predict the probability of occurrence of species at the spatial extent of whole South America and subsequently used to derive probabilities of occurrence on the local gradient (see Methods). SDMs performed generally well (prediction accuracy across all SDMs measured as true skill statistic = 0.87 ± 0.07). Based on these projections, we calculated FDis (weighted by SDM-derived occurrence probabilities) of projected future bird assemblages and compared these to the corresponding estimates from current assemblages for four different dispersal scenarios (*no range change*, *range contraction*, *expansion* and *shift*; Fig. 2). Projected changes in FDis showed a similar pattern across elevational levels, independent of the specific dispersal scenario (Fig. 2).

**Figure 3:**
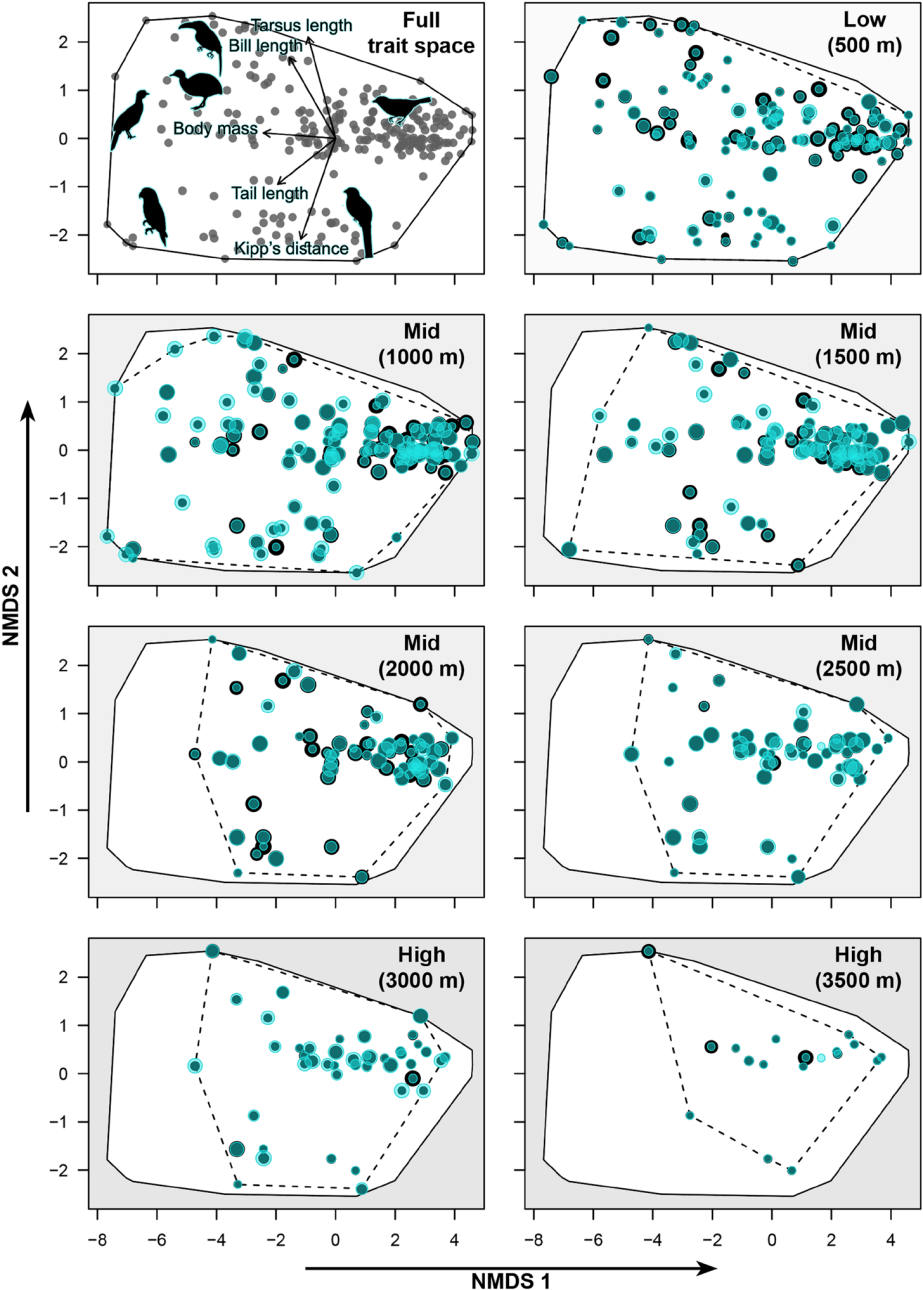
Projected changes in functional trait spaces of frugivorous bird assemblages under climate change assuming a *no range change scenario*. The functional trait space is derived from Nonmetric Multidimensional Scaling (NMDS, based on Euclidean distances) which condenses the dissimilarity of the first five PCoA axes, computed from ten morphological bird traits (bill width, bill length, bill height, tail length, tarsus length, tarsus sagittal width, tarsus lateral width, wing length, Kipp’s distance and body mass) onto two axes for visualization (NMDS 1, NMDS 2). Trait spaces are shown for the entire bird assemblage (upper left) and for assemblages on each of the seven elevational levels (500 m to 3500 m, at 500 m intervals). Black polygons illustrate the functional trait space of the entire bird assemblage. Black stippled polygons illustrate the functional trait space of the current local assemblage at the given elevation. Each dot represents one bird species (*n* = 232). Within each elevational level, dot size represents the probability of occurrence as derived from SDMs. Black dots represent current assemblages, transparent green dots represent occurrence probabilities under projected climate change (based on the RCP 8.5 scenario for 2080, MIROC5 climate model). Species-level changes are visible in four ways: (1) complete black dots indicate future loss of species, (2) complete light green dots indicate future immigration of species, (3) dark green dots with a black ring indicate future decline in occurrence probability, and (4) dark green dots with a light green ring indicate future increase in occurrence probability. Bird silhouettes in the upper left panel indicate the approximate location of key taxonomic groups in trait space (counterclockwise from top): toucans, tinamous, guans, parrots, trogons and tanagers. Fitted trait arrows indicate the relationship between five morphological bird traits (representing the different types of traits) and the ordination axes.

In line with the first expectation, we found a loss of FDis in the lowlands (500 m) (Fig. 2). This was largely consistent across dispersal scenarios and emission scenarios (RCP 6.0 and RCP 8.5) (Figs. 2 and S2, 500 m). The loss of FDis was due to a projected decrease in occurrence probabilities of species at the margins of the functional trait space (Figs. 3 and S3–5, 500 m).

In line with the second expectation, we found no or little projected change in FDis at the higher mid-elevation (2500 m), regardless of the dispersal scenario (Fig. 2). At this elevation, species with projected increases in occurrence probabilities had similar relative positions in functional trait space as species with projected decreasing occurrence probabilities, leading to little difference in FDis between current and projected future assemblages (Fig. 3, 2500 m). At lower mid-elevations (1000–2000 m), we found an increase in FDis (Fig. 2), in contrast to our expectation. At these elevations, occurrence probabilities of species at the margins of the functional trait space (*e*.*g*., larger bodied species) tended to increase, while occurrence probabilities of species in the centre of the trait space were projected to decrease (Figs. 3, S3–S5, 1000–2000 m). Due to increasing occurrence probabilities of functionally specialized species, the weight of these species in the calculation of FDis increases, resulting in a projected increase of FDis at lower mid-elevations.

Related to the third expectation, the models according the RCP emission scenario 8.5 suggested a slight increase in FDis at the highest elevations. This was partly due to a lack of species extinctions, as none of the species were projected to occur only beyond the tree line in future scenarios (Figs. 3, S3–S5, 3000–3500 m). In addition, many bird species, from across the trait space, were projected to immigrate to high elevations (Figs. 3, S3–S5). However, projected changes in FDis varied substantially among climate models (Fig. 2a,b) and emission scenarios (Supplementary Fig. S2) at high elevations. In line with our hypothesis, these results suggest relatively little changes in FDis at high elevations, although models revealed a large uncertainty in the projections at these elevations.

## Discussion

We combined SDMs with an analysis of functional dispersion (FDis, a measure of functional diversity) to explore how climate change might affect the functional diversity of frugivorous bird assemblages along an elevational gradient in the Andes. We found strong support for a reduction of FDis in the lowlands, a projected increase at lower mid-elevations, and little change at elevations higher than 2000 m. Under the assumption that climatic factors (especially temperature) are a major limiting factor for current and projected future occurrences of birds, these findings suggest that climate change has varying effects on avian functional diversity and associated ecosystem functions along tropical mountains.

Our projections consistently predicted reductions of FDis of frugivore assemblages in the lowlands. This decrease in FDis was found in all scenarios (four dispersal and two RCP scenarios). The consistent decrease in projected FDis in lowland assemblages under future conditions suggests that functionally specialized species (with a set of morphological traits that is different from the rest of the assemblage) might decrease in prevalence in the future, whereas species with more generalized morphologies and functional roles are less likely to be affected by future climate change. Because morphologically specialized species provide complementary functional roles to the assemblage^43^, their local disappearance may cause a loss of seed dispersal functions in the lowlands, especially for plant species with extreme morphologies, such as large-fruited plants^44,45^. Species that were projected to be lost from future lowland bird assemblages included, for instance, the functionally specialized Razor-billed Curassow (*Mitu tuberosum*), which was among the species with the largest body mass, and the Chestnut-eared and Ivory-billed Aracari (*Pteroglossus castanotis*, *P*. *azara*), which were among the species with the largest tarsus and bill lengths. The loss of such functionally specialized species may reduce long-distance seed dispersal that is required to maintain forest connectivity^46^ and colonization potential^47^. A non-random loss of functionally specialized species has been reported in response to land-use change^48^, *e*.*g*., the reduction of large-bodied frugivores due to habitat disturbance^49^ and hunting^50^. Our results indicate that climate change could have similar effects on functionally specialized species in tropical lowlands. This is especially alarming since the effects of human land use on bird diversity are also most severe at the base of tropical mountains^51^.

In contrast to losses in the lowlands, the models predicted an increase of FDis at the lower mid-elevations (1000–2000 m) of the gradient. This suggests that there is a high potential for species immigration at these elevations, in particular at the margins of the functional trait space. A major factor driving this trend is the projected upslope dispersal of lowland species in response to global warming^15,52^. Upslope dispersal potentially causes the immigration of functionally specialized species (that currently are very rare or absent from higher elevations) to the lower mid-elevations. Examples include the Oilbird (*Steatornis caripensis*), which is among the species with the most pointed wings, and the White-throated Toucan (*Ramphastos tucanus*), which is among the birds with the largest tarsus and bill lengths. The immigration of such species is likely to cause a pronounced change in FDis between current and projected future assemblages at these elevations, since these species occupy distinct positions in the functional trait space. In addition, FDis can also increase due to an increase in the occurrence probability of species with specialized morphologies that are already present in the current assemblages. Examples include the Razor-billed Curassow (*Mitu tuberosum*) and Ivory-billed Aracari (*Pteroglossus azara*) that are currently present at the mid-elevations, but were projected to have increasing probabilities of occurrence under projected future climate.

A high species turnover in bird assemblages at mid-elevations has been observed previously on tropical mountains (*e*.*g*., refs. ^4,53^). If these changes in species composition concern species with similar functional traits, this could foster redundancy among species as different species might be able to fulfil similar functional roles. When species with similar functional traits occur at different elevations of the Manú gradient, turnover of species driven by climate change could be an important mechanism in maintaining ecosystem functions, such as seed dispersal. Under such a scenario, the immigration of species with similar or new sets of functional traits could compensate for the loss of other species^4,54,55^. However, upward movements does not only depend on climate, but also on interactions among birds, such as interspecific competition^56^ or foraging interactions in mixed species flocks^57^. While knowledge on biotic factors shaping species’ distributions is available for some species, comprehensive information is currently lacking for species-rich tropical systems. In addition, species movements in response to climate change may depend on the availability of food resources^58^. Currently distributional patterns of birds and plants along this elevational gradient seem to broadly match^53,59^, while the future upslope dispersal of plants may lag behind that of birds^60^. Potential asynchronous species’ responses could force bird species immigrating from the lowlands to higher elevations to find new foraging plants^58^. Although many frugivorous birds tend to be relatively flexible in fruit choice^55^, the potential for rewiring species interactions, such as those between plants and birds, is not yet sufficiently understood^61,62^. Therefore, it is not yet fully clear how mid-elevation assemblages of frugivorous birds would re-assemble and to what extent vital seed dispersal functions could be maintained at these elevations in the future.

At the highest elevations close to the Manú tree line, we expected the extinction of certain species from the regional species pool, but all species currently present in the assemblage were projected to persist. This could be a consequence of the wide elevational ranges of species at these elevations. Consistent with Rapoport’s rule^63^, species in the lowlands tended to have narrow elevational ranges, whereas species at high elevations tended to have wide elevational ranges, covering several elevational levels. This pattern potentially mitigates decreases of FDis at high elevations since highland species are expected to have wide elevational ranges and potentially broad climatic niches, which would make them comparatively robust to future climate change. The Blue-and-yellow Tanager (*Thraupis bonariensis*), for example, occurs from 1000–3500 m and the Band-tailed Pigeon (*Patagioenas fasciata*) from 1500–3500 m, which means their current range is wider than the projected temperature shifts of 370 m and 520 m of elevation under RCP 6.0 and RCP 8.5, respectively (Supplementary Table S1).

In current highland assemblages, FDis is low compared to lowland assemblages, which might be a consequence of environmental filtering because only species with certain combinations of traits can persist under harsh environmental conditions^4,64^. At high elevations, the functional trait space is only sparsely filled and functional redundancy is lower than at lower elevations. Consequently, the increase or decrease in the probability of occurrence of a single species can have a large impact on the FDis of these assemblages. For example, the projected 80% decline in occurrence probability of the Grey-breasted Mountain-toucan (*Andigena hypoglauca*) would strongly affect FDis as the toucan fulfils a unique functional role in this assemblage. The highest elevation on our gradient (3500 m) coincides with the current tree line^65^, which might constrain bird movements to higher elevations^18^. Little functional redundancy and idiosyncratic changes in the species composition of frugivorous bird assemblages may, therefore, lead to unpredictable assemblage dynamics at the tree line of tropical mountains.

In conclusion, we show that the combination of SDMs and functional dispersion as a measure of functional diversity is a promising way to study potential changes in the functional diversity of ecological assemblages under future climate change. While remaining correlative, our study suggests that FDis of bird assemblages at different elevations of tropical mountains could be affected differently by projected global warming. First, lowland assemblages might face a loss of functional diversity in the future because functionally specialized species appear to be more vulnerable to climate change than functional generalists. Second, assemblages at mid-elevations are likely to reshuffle in the future, which may lead to the immigration of functional specialists and a high species turnover in these assemblages which could buffer climate-change impacts at these elevations. Our findings are relevant for forest ecosystems along tropical mountains as the projected elevation-specific changes in frugivorous bird assemblages could have important feedbacks on seed dispersal and plant regeneration.

## Methods

### Bird data

Current elevational ranges of the 245 frugivorous bird species occurring in the Manú biosphere reserve (Andes of southeast Peru, 250–3750 m) were obtained from Dehling *et al*.^4^, who combined data (derived from bird observations via sight, sound or sign records) from Walker *et al*.^66^ and Merkord^67^, complemented with data collected by D.M. Dehling during field work in Manú between December 2009 and September 2011. The data set reflected observations from 1973 to 2011 and contained all frugivorous bird species occurring on this specific gradient, including rare species^66^. We used these local elevational distribution ranges of birds to derive the assemblage composition at seven discrete elevational levels of the elevational gradient in the Manú biosphere reserve (500–3500 m, at 500 m intervals).

We additionally downloaded occurrence records from continental South America for each bird species in our dataset from the Global Biodiversity Information Facility (GBIF; www.gbif.org) which we used to fit species-specific SDMs. The data were subjected to a comprehensive quality check, in which we deleted all entries without coordinates, with coordinates located outside the extent of South America (81.5° W to 34.5° W, 56° S to 12.5° N), and all entries where coordinates and country of origin did not match. This resulted in a total of 16–9,138 occurrence records per species (median = 663). Species with less than 40 occurrence records (*n* = 5 spp) were considered unsuitable for modelling and excluded from further analysis. In addition, we excluded all migratory species that are only present in the region in a certain season (*n* = 8 spp). This resulted in a species pool of 232 frugivorous bird species with 46–9,138 occurrence records (median = 668.5) per species. Our dataset included species that are widely distributed across South America (*e*.*g*., the White-eyed Parakeet *Aratinga leucophthalma* with 2308 GBIF occurrence records across South America) as well as narrowly distributed species (*e*.*g*., the Scarlet-hooded Barbet *Eubucco tucinkae* with 86 GBIF occurrence records).

### Climate

Climatic data on mean annual temperature and mean annual precipitation were compiled across South America (for fitting continental SDMs) and at the local level along the elevational gradient of the Manú biosphere reserve (for elevational level SDM projections). Mean annual temperature and precipitation for each of the seven elevational levels were compiled from literature^68,69^ and from local measurements by W. Farfan-Rios (elevation 3500 m) and C. Beirne (CREES, MLC, elevation 500 m). The use of local mean annual temperature and precipitation data was preferred over the use of global climate data since they were measured on site and therefore were not affected by interpolation between climate stations in mountainous areas.

To compile the future climate projections for the seven elevational levels, we downloaded current and projected future climate rasters for mean annual temperature and precipitation from WorldClim^41^ at a spatial resolution of 2.5 minutes (approx. 4.6 km on the equator). Current climate values from WorldClim are based on a 30-year time period between 1961–1990 (“current”) and the future projected climate values on the 20-year time period between 2061–2080 (“2080”). Future climate projections were downloaded for five general circulation models (GCMs) from the Fifth Assessment Report of the Intergovernmental Panel on Climate Change (IPCC 2013^42^); CCSM4 (CC), HadGEM2-ES (HE), MIROC5 (MC), MRI-CGCM3 (MG) and NorESM1-M (NO)^41,42^, using two representative concentration pathways (RCP), RCP 6.0 (assumed global average increase of 2.85 ± 0.62 °C in MAT) and RCP 8.5 (assumed global average increase of 4.02 ± 0.80 °C in MAT). Projected climate anomalies were calculated by subtracting the current mean annual temperature and precipitation rasters from the projected future mean annual temperature and precipitation rasters for each climate model. For the elevational gradient, we extracted the mean annual temperature and precipitation anomaly values from the locations of the seven studied elevational levels (in seven grid cells of 2.5 minutes resolution). Anomalies were added to the mean annual temperature and precipitation values from each respective elevational level to obtain the future projected climate along the Manú elevational gradient.

### Species distribution models (SDM)

We used species distribution models (SDMs) to obtain the current and projected future probability of occurrence of the 232 bird species within each of the seven elevational levels of the Manú biosphere reserve. All SDMs were fitted on a spatial extent of continental South America to capture the whole climatic niche space of each species. Since spatial sampling bias (*e*.*g*., towards easily accessible areas or due to differences in sampling intensity among taxa) can affect the performance of SDMs, we minimized this effect by following the suggestions from Phillips *et al*.^70^ through restricting the background data to an extent with the same spatial bias as the pooled data of all species occurrences. Hence, we downloaded GBIF occurrence records for all bird species in South America and restricted the background to only those grid cells in which at least one bird species was observed. This resulted in a background of 34,297 grid cells (2.5 minutes resolution) across the entire extent of continental South America.

To identify the best performing model for each bird species, we compared five SDM algorithms: generalized linear models (GLM), generalized additive models (GAM), boosted regression trees (GBM), random forest (RF) and maximum entropy models (MAXENT)^71^. SDMs were based on mean annual temperature and precipitation values (WorldClim^41^). As a random cross-validation of model performance, species occurrence data were split into 80% calibration data and 20% evaluation data. Prevalence was set to 0.5, to give presences and absences the same importance in the calibration process. We followed the suggestions of Barbet-Massin *et al*.^72^, who provide a comparative analysis from which they derive recommendations on how, where and how many pseudo-absences should be generated for various SDM algorithms. Prior to model fitting, we consequently selected 10,000 random PAs from the background data for fitting GLM and GAM models^72^. For the GBM and RF models, we selected the same amount of PAs as there were presence data (occurrence records) available for each species^72^. For these two methods, we ran the models ten times per species, each run with a new selection of PAs. The average of these ten models was then considered further. For MAXENT, a simple form of a Poisson point-process model, the presence records were contrasted against the entire background because presence/absence is not measured^72,73^. All SDMs were evaluated with the true skill statistic (TSS) evaluation metric, *i*.*e*., the sum of sensitivity and specificity minus one (the sensitivity is the proportion of correctly predicted presences, and the specificity is the proportion of correctly predicted absences), GBM and RF were evaluated with the average TSS value of the ten averaged models. The cut-off value of the models, *i*.*e*., the threshold value of the probability of occurrence that maximizes the TSS metric for each model, ranged between 0.62–0.85 (see Supplementary Table S5). To optimize the SDM projection per species, we selected the model algorithm with the highest TSS value for the respective species (see Supplementary Table S5 for model evaluation metrics for all SDMs). The TSS metric for the selected SDMs ranged between 0.63–0.99. The interpretation of SDMs, as well as any other correlative approach, has to be done with caution because correlations do not necessarily imply causation.

The best performing SDM was used to determine the probability that a species could occur on each of the seven elevational levels, under current and future conditions. To do this, we projected the SDMs (BIOMOD_Projection in “biomod2” package in R^71^) using the current local climate as well as the 10 projected future local climates (2080; RCP 6.0 and RCP 8.5 under five GCMs). We used the full continuum of occurrence probabilities and interpreted a low probability of occurrence of a species as indicating a low representation of its functional type in the respective assemblage. Hence, no presence/absence threshold was required in the analysis and species were weighted continuously by their current and projected occurrence probability.

### Elevational bird assemblages

We compiled current bird assemblages for each elevational level along the Manú elevational gradient using the current elevational range data of birds (see above and Supplementary Table S1). A species was considered to be present in the assemblage of an elevational level when that elevation fell within the elevational range of a species. We are aware that local bird communities within elevational levels might show some variation in species composition and that not all species may interact at a given locality. The data compilation resulted in a binary presence/absence matrix of the 232 bird species at the seven elevational levels (500–3500 m, at 500 m intervals).

Future bird assemblages were based on SDM-derived projected probabilities of occurrences and were further refined by four temperature-driven dispersal scenarios (*no range change*, *range contraction*, *range expansion*, and *range shift*, Fig. 2). Under the *no range change* scenario, projected changes in the elevational ranges of species were not taken into account and the future assemblages were based on the SDM-derived probabilities of occurrences and the current elevational ranges (Fig. 2a). Under the *range contraction*, *range expansion*, and *range shift* scenarios, we tested three scenarios in which the future elevational ranges of species were altered according to temperature changes with altitude (see below), under the assumption that species have to move upwards to track their climate niche^74^. Under the *range contraction scenario*, bird species could only have an upward movement of their current lower range limit, while the upper limit remained constant. This leads to a contraction of species ranges; *i*.*e*., species could not adapt to future climatic conditions at the lower range limit and were unable to disperse upslope (Fig. 2b). Under the *range expansion scenario*, bird species were projected to expand their range upwards, while the lower limit of their elevational range remained the same; *i*.*e*., species could adapt to future conditions at their lower range limit and could disperse upslope (Fig. 2c). Finally, under the *range shift scenario*, bird species could move their complete range upwards, *i*.*e*., both the lower and upper limit changed and species fully tracked their temperature niche upslope along the mountain (Fig. 2d).

To project future elevational ranges of species according to these dispersal scenarios, we acquired annual mean tropospheric lapse-rate values (*i*.*e*., how temperature decreases with an increase in altitude) at a resolution of 2.5° (approx. 276 km at the equator) for the period 1948–2001 from Mokhov & Akperov^75^. This lapse-rate raster was bilinearly interpolated to match the resolution of 2.5 minutes of the mean annual temperature (MAT) rasters and ranged between 6.17 °C/km and 6.22 °C/km. Following La Sorte & Jetz^76^, we then estimated the projected vertical shifts in temperature by dividing the projected temperature anomaly (°C) by the cell’s tropospheric lapse rate (°C/km). Projected vertical distances (km) were extracted for the entire Manú elevational gradient, *i*.*e*., for the seven studied elevational levels (seven grid cells, 500–3500 m, at 500 m intervals) and two additional grid cells which represent the lowest (250 m) and highest (3750 m) point of the Manú elevational gradient (all grid cells at a 2.5 minutes resolution). We then computed species-specific vertical distance as the mean across the vertical distance values of all elevational levels at which the respective species currently occurs. The mean projected vertical distance per bird species was calculated for the year 2080, two emission scenarios (RCP 6.0 and RCP 8.5) and five GCMs (CC, HE, MC, MG and NO). The mean vertical distance by which a species would change its elevational distribution to track its temperature niche, according to our projections, ranged between 290 m and 490 m under RCP 6.0 and between 465 m and 700 m under RCP 8.5.

The future elevational ranges of species were subsequently calculated by adding the mean projected vertical distance per species to the lower and/or upper values of their current elevational range, according to the respective dispersal scenario (see Supplementary Table S1 for current and projected future elevational ranges of all bird species). Future assemblages under the dispersal scenarios *range contraction*, *range expansion* and *range shift* were subsequently derived by cropping the SDM-derived probabilities of occurrence of each species by the respective future elevational range of the species. Hence, if a species was projected to occur on an elevational level by its SDM, while the respective elevational level was not included in its future elevational range according to the respective dispersal scenario, the species was considered absent and its probability to occur was set to 0. Only under the scenario of *no range change*, assemblages were directly defined by the SDM-derived probabilities of occurrence, according to the current elevational ranges of species.

Future assemblages for the four dispersal scenarios were derived for two RCP scenarios (RCP 6.0, RCP 8.5), leading to eight potential future bird assemblages per elevational level for each of the five GCMs (see Supplementary Tables S2–S4 for current and future probabilities of occurrence of all bird species under the various future scenarios).

### Functional diversity of bird assemblages

To quantify functional diversity, we used ten morphological traits that are relevant for seed dispersal by frugivorous birds^4^. Traits related to beak morphology influence seed dispersal by imposing direct constraints on the possibility that a bird is able to handle and swallow a fruit^21,59^. Traits related to flight performance and bipedal locomotion influence which fruits a foraging bird species encounters^22,23^. Finally, body mass (g) gives an indication of the energy requirements of a bird and therefore the type of fruit it might prefer^24,25^. Information on avian body mass was derived from Dunning^77^. All other traits were measured on at least four museum specimens for each of the 232 bird species, mainly following the methodology from Eck *et al*.^78^. We used the mean trait value for each species. Specifically, the ten traits included bill width, length and height (mm), tail length (mm), tarsus length and tarsus sagittal/lateral width (mm), wing length (mm), Kipp’s distance (the distance between the tip of the first secondary and the wing tip, measured on the folded wing, in mm), and body mass (g)^4^.

For all current and projected future assemblages, according to the four dispersal scenarios (*no range change*, *range contraction*, *expansion* and *shift*) under RCP 6.0 and RCP 8.5 for five GCMs, we calculated functional dispersion as measure of functional diversity to determine potential impacts of climate change on FDis of bird assemblages along the elevational gradient. Functional dispersion (FDis) quantifies the weighted averaged distance of all species to the weighted centroid of an assemblage^31^ and has been shown to be a robust measure of functional diversity. Specifically, FDis is largely independent of species richness^31^, and log-transformed FDis and species richness of current bird assemblages were not significantly correlated in our dataset (Pearson *r* = 0.53, *P* = 0.22, Supplementary Fig. S1). We weighted FDis by the probability of occurrence of each species in current and projected future assemblages (derived from SDMs). Weighting FDis with the probability of occurrence in current and future assemblages does not imply that occurrence probabilities are a proxy for species abundances (see ref. ^79^), but assumes that the probability that a species occurs in a local assemblage is associated with the likelihood that its functional role is realized in this assemblage. Consequently, species with a lower probability of occurrence in an assemblage will influence FDis less than species which are projected to have a high probability of occurrence in the assemblage. We calculated FDis with the dbFD function in the “FD” package in R^80^, based on the ten log-transformed morphological bird traits (see above). Dissimilarities between species were calculated based on the Euclidean trait distance between species. These dissimilarities were used to project the variability in the ten morphological traits onto ten axes in a Principle Coordinate Analysis (PCoA)^29^. Ordination analysis, such as PCoA, do not suffer from collinearity among variables (for Pearson’s correlation coefficients between the ten morphological traits, see Supplementary Table S6). In addition, the PCoA provides the possibility to study species’ differences in trait combinations, rather than in single trait values, which is why we used the PCoA axes and not the raw trait data^29^.

Following Maire *et al*.^81^, we evaluated how many dimensions, *i*.*e*., PCoA axes, should be considered in the calculation of FDis. The quality of the functional trait spaces was calculated as the mean squared deviation (mSD) between the initial distances between species (based on raw trait values) and the distance between species in the projected functional trait space^81^. The functional trait space with the lowest mSD is considered to have the highest quality, *i*.*e*., this trait space is the best representation of the initial functional trait values. We tested nine functional trait spaces (from two to ten dimensions). mSD ranged from 0.01 to 0.03, with the lowest value belonging to the functional trait space with five dimensions. The five PCoA axes explained 98.1% of total variation among species (78.6%, 9.1%, 5.3%, 2.9% and 2.2%, respectively). Consequently, FDis values were calculated from the five-dimensional trait space. The centre of the trait space corresponds to average combinations of functional traits in the trait space. This is where the species with the most widespread trait combinations are located. The margin of the trait space corresponds to more extreme trait combinations and is where functionally more specialized species are located.

To visualize the functional trait spaces of current and projected future bird assemblages on two trait dimensions (see Fig. 3), we computed the Euclidean distances between species in the functional trait space of the first five PCoA axes and used these distances in a Nonmetric Multidimensional Scaling (NMDS, metaMDS in “vegan” package in R). The NMDS condensed the variation of the five PCoA axes onto two axes. The scaling achieved a stable solution (stress value = 0.07) and the two NMDS dimensions can therefore be considered representative for visualizing the overall trait variation among species based on the ten morphological traits. We fitted trait-arrows to the NMDS space to indicate how five of the ten morphological bird traits, representing the different types of traits (body mass, bill length, Kipp’s distance, tail length, tarsus length), were related to the ordination axes. These arrows indicate that variation in body mass was primarily related to the first NMDS axis, while the second axes primarily represented variation in Kipp’s distance (higher at negative values) and tarsus length (higher at positive values) (Fig. 3, first panel).

## Supplementary information

Supporting Information

## Data Availability

Data will be made available on Dryad upon acceptance.
