# Supplementary material for: Projected impacts of climate change on functional diversity of frugivorous birds along a tropical elevational gradient
Source: Sci Rep. 2019 Nov 27;9:17708. doi: 10.1038/s41598-019-53409-6 (PMC6881284; doi:10.1038/s41598-019-53409-6)
Supplement: Supplementary file 1 — Supporting Information [file 41598_2019_53409_MOESM1_ESM.pdf]

Supporting Information

Irene M.A. Bender, W. Daniel Kissling, Katrin Böhning-Gaese, Isabell Hensen, Ingolf Kühn, Larissa Nowak, Till Töpfer, Thorsten Wiegand, D. Matthias Dehling and Matthias Schleuning. **Projected impacts of climate change on functional diversity of frugivorous birds along a tropical elevational gradient.**

Table S1. Current and future elevational ranges of all 232 frugivorous bird species.

Table S2. Current and future probabilities of occurrence derived from SDMs for all 232 frugivorous bird species.

Table S3. Current and future assemblage composition at all seven elevational levels, including probabilities of occurrence. Based on the MIROC5 climate model, RCP 6.0.

Table S4. Current and future assemblage composition at all seven elevational levels, including probabilities of occurrence. Based on the MIROC5 climate model, RCP 8.5.

Table S5. Model evaluation metrics for each bird species and SDM algorithm.

Table S6. Pearson's correlation coefficients between morphological traits.

Figure S1. Species richness and functional dispersion under current conditions along the forested part of an elevational gradient in the Peruvian Andes.

Figure S2. Potential changes in functional dispersion of bird assemblages under projected future climate change, RCP 6.0.

Figure S3. Projected changes in functional trait spaces of frugivorous birds under climate change assuming a *range contraction scenario,* RCP 8.5.

Figure S4. Projected changes in functional trait spaces of frugivorous birds under climate change assuming a *range expansion scenario,* RCP 8.5.

Figure S5. Projected changes in functional trait spaces of frugivorous birds under climate change assuming a *range shift scenario*, RCP 8.5.

Table S1. Current and future elevational ranges of all 232 frugivorous bird species of the Manú Biosphere Reserve, Peru, based on the MIROC5 climate model. Current elevational ranges and bird taxonomy are derived from Dehling *et al.* (2014). Future elevational ranges were projected based on vertical distance movements of each bird species based on future temperature scenarios and tropospheric lapse-rates. Future temperature scenarios were based on two representative concentration pathways RCP 6.0 and RCP 8.5. Future elevational ranges were applied to three of the potential migration scenarios: *range contraction* (where the lower range limit moves upwards), *range expansion* (where the upper range limit moves upwards), and *range shift* where both the lower and upper range limit move upwards.

		Current		Range expansion			Range contraction			Range shift			
					RCP 6.0	RCP 8.5	RCP 6.0	RCP 8.5		RCP 6.0		RCP 8.5	
Family	Bird species	min	max	min	max	max	min	min	max	min	max	min	max
Cardinalidae	*Cyanocompsa cyanoides*	250	1400	250	1786	1953	636	803	1400	636	1786	803	1953
Cardinalidae	*Pheucticus aureoventris*	600	3250	600	3622	3774	972	1124	3250	972	3622	1124	3774
Cardinalidae	*Pheucticus chrysogaster*	650	3500	650	3869	4023	1019	1173	3500	1019	3869	1173	4023
Cardinalidae	*Saltator aurantiirostris*	2600	3500	2600	3864	4026	2964	3126	3500	2964	3864	3126	4026
Cardinalidae	*Saltator coerulescens*	250	900	250	1294	1463	644	813	900	644	1294	813	1463
Cardinalidae	*Saltator grossus*	250	1006	250	1392	1559	636	803	1006	636	1392	803	1559
Cardinalidae	*Saltator maximus*	250	2507	250	2886	3042	629	785	2507	629	2886	785	3042
Columbidae	*Geotrygon montana*	250	1489	250	1875	2042	636	803	1489	636	1875	803	2042
Columbidae	*Patagioenas cayennensis*	250	900	250	1294	1463	644	813	900	644	1294	813	1463
Columbidae	*Patagioenas fasciata*	1200	3530	1200	3899	4051	1569	1721	3530	1569	3899	1721	4051
Columbidae	*Patagioenas plumbea*	250	1991	250	2374	2535	633	794	1991	633	2374	794	2535
Columbidae	*Patagioenas subvinacea*	250	1550	250	1933	2094	633	794	1550	633	1933	794	2094
Corvidae	*Cyanocorax cyanomelas*	250	1100	250	1486	1653	636	803	1100	636	1486	803	1653
Corvidae	*Cyanocorax violaceus*	250	1400	250	1786	1953	636	803	1400	636	1786	803	1953
Corvidae	*Cyanocorax yncas*	1150	2200	1150	2572	2718	1522	1668	2200	1522	2572	1668	2718
Cotingidae	*Ampelioides tschudii*	700	1400	700	1771	1932	1071	1232	1400	1071	1771	1232	1932
Cotingidae	*Ampelion rubrocristatus*	2500	3450	2500	3822	3976	2872	3026	3450	2872	3822	3026	3976
Cotingidae	*Ampelion rufaxilla*	1600	2850	1600	3222	3368	1972	2118	2850	1972	3222	2118	3368
Cotingidae	*Cephalopterus ornatus*	250	1650	250	2033	2194	633	794	1650	633	2033	794	2194
Cotingidae	*Conioptilon mcilhennyi*	250	450	250	852	1029	652	829	450	652	852	829	1029
Cotingidae	*Cotinga cayana*	250	800	250	1194	1363	644	813	800	644	1194	813	1363
Cotingidae	*Cotinga maynana*	250	1000	250	1386	1553	636	803	1000	636	1386	803	1553
Cotingidae	*Gymnoderus foetidus*	250	500	250	894	1063	644	813	500	644	894	813	1063
Cotingidae	*Iodopleura isabellae*	250	850	250	1244	1413	644	813	850	644	1244	813	1413
Cotingidae	*Laniocera hypopyrra*	250	750	250	1144	1313	644	813	750	644	1144	813	1313
Cotingidae	*Lipaugus vociferans*	250	800	250	1194	1363	644	813	800	644	1194	813	1363
Cotingidae	*Oxyruncus cristatus*	850	1300	850	1671	1832	1221	1382	1300	1221	1671	1382	1832
Cotingidae	*Pipreola arcuata*	1821	3650	1821	4018	4172	2189	2343	3650	2189	4018	2343	4172
Cotingidae	*Pipreola frontalis*	1000	1780	1000	2151	2305	1371	1525	1780	1371	2151	1525	2305
Cotingidae	*Pipreola intermedia*	1459	3101	1459	3473	3623	1831	1981	3101	1831	3473	1981	3623
Cotingidae	*Porphyrolaema porphyrolaema*	250	450	250	852	1029	652	829	450	652	852	829	1029
Cotingidae	*Querula purpurata*	250	1050	250	1436	1603	636	803	1050	636	1436	803	1603
Cotingidae	*Rupicola peruvianus*	650	2800	650	3172	3321	1022	1171	2800	1022	3172	1171	3321
Cotingidae	*Snowornis subalaris*	850	1350	850	1721	1882	1221	1382	1350	1221	1721	1382	1882
Cotingidae	*Tityra cayana*	250	450	250	852	1029	652	829	450	652	852	829	1029
Cotingidae	*Tityra inquisitor*	250	670	250	1064	1233	644	813	670	644	1064	813	1233
Cotingidae	*Tityra semifasciata*	250	1500	250	1883	2044	633	794	1500	633	1883	794	2044
Cracidae	*Aburria aburri*	650	1948	650	2319	2473	1021	1175	1948	1021	2319	1175	2473

Cracidae	*Mitu tuberosum*	250	1000	250	1386	1553	636	803	1000	636	1386	803	1553
Cracidae	*Ortalis guttata*	250	1600	250	1983	2144	633	794	1600	633	1983	794	2144
Cracidae	*Penelope jacquacu*	250	1500	250	1883	2044	633	794	1500	633	1883	794	2044
Cracidae	*Penelope montagnii*	900	3200	900	3572	3724	1272	1424	3200	1272	3572	1424	3724
Cracidae	*Pipile cumanensis*	250	500	250	894	1063	644	813	500	644	894	813	1063
Icteridae	*Cacicus cela*	250	1050	250	1436	1603	636	803	1050	636	1436	803	1603
Icteridae	*Cacicus chrysonotus*	2010	3342	2010	3714	3868	2382	2536	3342	2382	3714	2536	3868
Icteridae	*Cacicus haemorrhous*	250	1000	250	1386	1553	636	803	1000	636	1386	803	1553
Icteridae	*Clypicterus oseryi*	250	800	250	1194	1363	644	813	800	644	1194	813	1363
Icteridae	*Icterus icterus*	250	800	250	1194	1363	644	813	800	644	1194	813	1363
Icteridae	*Molothrus oryzivorus*	250	800	250	1194	1363	644	813	800	644	1194	813	1363
Icteridae	*Psarocolius angustifrons*	250	2083	250	2464	2622	631	789	2083	631	2464	789	2622
Icteridae	*Psarocolius atrovirens*	983	2750	983	3122	3271	1355	1504	2750	1355	3122	1504	3271
Icteridae	*Psarocolius bifasciatus*	250	970	250	1364	1533	644	813	970	644	1364	813	1533
Icteridae	*Psarocolius decumanus*	250	1320	250	1706	1873	636	803	1320	636	1706	803	1873
Odontophoridae	*Odontophorus balliviani*	800	3100	800	3472	3624	1172	1324	3100	1172	3472	1324	3624
Odontophoridae	*Odontophorus speciosus*	1000	2100	1000	2472	2622	1372	1522	2100	1372	2472	1522	2622
Odontophoridae	*Odontophorus stellatus*	250	1050	250	1436	1603	636	803	1050	636	1436	803	1603
Picidae	*Celeus elegans*	250	500	250	894	1063	644	813	500	644	894	813	1063
Picidae	*Celeus flavus*	250	500	250	894	1063	644	813	500	644	894	813	1063
Picidae	*Celeus grammicus*	250	1200	250	1586	1753	636	803	1200	636	1586	803	1753
Picidae	*Celeus spectabilis*	250	500	250	894	1063	644	813	500	644	894	813	1063
Picidae	*Celeus torquatus*	250	500	250	894	1063	644	813	500	644	894	813	1063
Picidae	*Colaptes rubiginosus*	750	2010	750	2382	2532	1122	1272	2010	1122	2382	1272	2532
Pipridae	*Chiroxiphia boliviana*	950	2033	950	2405	2555	1322	1472	2033	1322	2405	1472	2555
Pipridae	*Chiroxiphia pareola*	250	700	250	1094	1263	644	813	700	644	1094	813	1263
Pipridae	*Lepidothrix coeruleocapilla*	797	1709	797	2080	2234	1168	1322	1709	1168	2080	1322	2234
Pipridae	*Lepidothrix coronata*	250	900	250	1294	1463	644	813	900	644	1294	813	1463
Pipridae	*Machaeropterus pyrocephalus*	250	1429	250	1815	1982	636	803	1429	636	1815	803	1982
Pipridae	*Manacus manacus*	450	975	450	1362	1523	837	998	975	837	1362	998	1523
Pipridae	*Neopelma sulphureiventer*	250	500	250	894	1063	644	813	500	644	894	813	1063
Pipridae	*Pipra chloromeros*	250	1400	250	1786	1953	636	803	1400	636	1786	803	1953
Pipridae	*Pipra fasciicauda*	250	1055	250	1441	1608	636	803	1055	636	1441	803	1608
Pipridae	*Xenopipo holochlora*	450	1100	450	1479	1640	829	990	1100	829	1479	990	1640
Pipridae	*Xenopipo unicolor*	1000	1800	1000	2171	2325	1371	1525	1800	1371	2171	1525	2325
Psittacidae	*Amazona farinosa*	250	1350	250	1736	1903	636	803	1350	636	1736	803	1903
Psittacidae	*Amazona mercenaria*	826	3100	826	3472	3624	1198	1350	3100	1198	3472	1350	3624
Psittacidae	*Amazona ochrocephala*	250	850	250	1244	1413	644	813	850	644	1244	813	1413
Psittacidae	*Ara ararauna*	250	1000	250	1386	1553	636	803	1000	636	1386	803	1553
Psittacidae	*Ara chloropterus*	250	1390	250	1776	1943	636	803	1390	636	1776	803	1943
Psittacidae	*Ara macao*	250	1000	250	1386	1553	636	803	1000	636	1386	803	1553
Psittacidae	*Ara militaris*	600	1716	600	2087	2241	971	1125	1716	971	2087	1125	2241
Psittacidae	*Ara severus*	250	1236	250	1622	1789	636	803	1236	636	1622	803	1789
Psittacidae	*Aratinga leucophthalma*	250	1650	250	2033	2194	633	794	1650	633	2033	794	2194
Psittacidae	*Aratinga mitrata*	1621	3414	1621	3786	3937	1993	2144	3414	1993	3786	2144	3937
Psittacidae	*Aratinga weddellii*	250	700	250	1094	1263	644	813	700	644	1094	813	1263
Psittacidae	*Bolborhynchus lineola*	1100	3383	1100	3755	3905	1472	1622	3383	1472	3755	1622	3905
Psittacidae	*Bolborhynchus orbygnesius*	2500	3500	2500	3867	4023	2867	3023	3500	2867	3867	3023	4023
Psittacidae	*Brotogeris cyanoptera*	250	500	250	894	1063	644	813	500	644	894	813	1063
Psittacidae	*Brotogeris sanctithomae*	250	500	250	894	1063	644	813	500	644	894	813	1063

Psittacidae	*Forpus modestus*	250	500	250	894	1063	644	813	500	644	894	813	1063
Psittacidae	*Nannopsittaca dachilleae*	250	1088	250	1474	1641	636	803	1088	636	1474	803	1641
Psittacidae	*Orthopsittaca manilata*	250	650	250	1044	1213	644	813	650	644	1044	813	1213
Psittacidae	*Pionites leucogaster*	250	500	250	894	1063	644	813	500	644	894	813	1063
Psittacidae	*Pionus menstruus*	250	2383	250	2764	2922	631	789	2383	631	2764	789	2922
Psittacidae	*Pionus tumultuosus*	1100	3032	1100	3404	3554	1472	1622	3032	1472	3404	1622	3554
Psittacidae	*Primolius couloni*	250	1300	250	1686	1853	636	803	1300	636	1686	803	1853
Psittacidae	*Pyrilia barrabandi*	250	500	250	894	1063	644	813	500	644	894	813	1063
Psittacidae	*Pyrrhura roseifrons*	250	1000	250	1386	1553	636	803	1000	636	1386	803	1553
Psittacidae	*Pyrrhura rupicola*	250	1100	250	1486	1653	636	803	1100	636	1486	803	1653
Psittacidae	*Touit huetii*	300	1300	300	1679	1840	679	840	1300	679	1679	840	1840
Psophiidae	*Psophia leucoptera*	250	1050	250	1436	1603	636	803	1050	636	1436	803	1603
Ramphastidae	*Andigena hypoglauca*	1450	3500	1450	3869	4021	1819	1971	3500	1819	3869	1971	4021
Ramphastidae	*Aulacorhynchus coeruleicinctis*	1059	2937	1059	3309	3455	1431	1577	2937	1431	3309	1577	3455
Ramphastidae	*Aulacorhynchus derbianus*	800	1852	800	2223	2377	1171	1325	1852	1171	2223	1325	2377
Ramphastidae	*Aulacorhynchus prasinus*	250	1500	250	1883	2044	633	794	1500	633	1883	794	2044
Ramphastidae	*Capito auratus*	250	1350	250	1736	1903	636	803	1350	636	1736	803	1903
Ramphastidae	*Eubucco richardsoni*	250	1000	250	1386	1553	636	803	1000	636	1386	803	1553
Ramphastidae	*Eubucco tucinkae*	250	800	250	1194	1363	644	813	800	644	1194	813	1363
Ramphastidae	*Eubucco versicolor*	750	2130	750	2502	2652	1122	1272	2130	1122	2502	1272	2652
Ramphastidae	*Pteroglossus azara*	250	1200	250	1586	1753	636	803	1200	636	1586	803	1753
Ramphastidae	*Pteroglossus beauharnaesii*	250	951	250	1345	1514	644	813	951	644	1345	813	1514
Ramphastidae	*Pteroglossus castanotis*	250	1000	250	1386	1553	636	803	1000	636	1386	803	1553
Ramphastidae	*Pteroglossus inscriptus*	250	500	250	894	1063	644	813	500	644	894	813	1063
Ramphastidae	*Ramphastos tucanus*	250	876	250	1270	1439	644	813	876	644	1270	813	1439
Ramphastidae	*Ramphastos vitellinus*	250	600	250	994	1163	644	813	600	644	994	813	1163
Ramphastidae	*Selenidera reinwardtii*	250	1682	250	2065	2226	633	794	1682	633	2065	794	2226
Steatornithidae	*Steatornis caripensis*	450	900	450	1287	1448	837	998	900	837	1287	998	1448
Thraupidae	*Anisognathus igniventris*	2525	3600	2525	3964	4126	2889	3051	3600	2889	3964	3051	4126
Thraupidae	*Anisognathus somptuosus*	1375	3000	1375	3372	3522	1747	1897	3000	1747	3372	1897	3522
Thraupidae	*Buthraupis montana*	2312	3250	2312	3622	3776	2684	2838	3250	2684	3622	2838	3776
Thraupidae	*Catamblyrhynchus diadema*	2000	3250	2000	3622	3773	2372	2523	3250	2372	3622	2523	3773
Thraupidae	*Chlorochrysa calliparaea*	1000	2200	1000	2572	2722	1372	1522	2200	1372	2572	1522	2722
Thraupidae	*Chlorophanes spiza*	250	1430	250	1816	1983	636	803	1430	636	1816	803	1983
Thraupidae	*Chlorophonia cyanea*	250	2115	250	2496	2654	631	789	2115	631	2496	789	2654
Thraupidae	*Chlorornis riefferii*	2100	3215	2100	3587	3741	2472	2626	3215	2472	3587	2626	3741
Thraupidae	*Chlorospingus canigularis*	1000	1600	1000	1971	2125	1371	1525	1600	1371	1971	1525	2125
Thraupidae	*Chlorospingus flavigularis*	800	2350	800	2722	2872	1172	1322	2350	1172	2722	1322	2872
Thraupidae	*Chlorospingus ophthalmicus*	1000	2561	1000	2933	3082	1372	1521	2561	1372	2933	1521	3082
Thraupidae	*Chlorospingus parvirostris*	1100	2600	1100	2972	3118	1472	1618	2600	1472	2972	1618	3118
Thraupidae	*Cissopis leverianus*	250	1600	250	1983	2144	633	794	1600	633	1983	794	2144
Thraupidae	*Conirostrum cinereum*	2850	3500	2850	3864	4026	3214	3376	3500	3214	3864	3376	4026
Thraupidae	*Conirostrum speciosum*	250	900	250	1294	1463	644	813	900	644	1294	813	1463
Thraupidae	*Cyanerpes caeruleus*	250	1400	250	1786	1953	636	803	1400	636	1786	803	1953
Thraupidae	*Cyanerpes cyaneus*	250	1000	250	1386	1553	636	803	1000	636	1386	803	1553
Thraupidae	*Dacnis cayana*	250	1550	250	1933	2094	633	794	1550	633	1933	794	2094
Thraupidae	*Dacnis flaviventer*	250	1000	250	1386	1553	636	803	1000	636	1386	803	1553
Thraupidae	*Dacnis lineata*	250	1300	250	1686	1853	636	803	1300	636	1686	803	1853
Thraupidae	*Delothraupis castaneoventris*	2305	3520	2305	3887	4043	2672	2828	3520	2672	3887	2828	4043
Thraupidae	*Diglossa brunneiventris*	2518	3560	2518	3924	4086	2882	3044	3560	2882	3924	3044	4086

Thraupidae	*Diglossa caerulescens*	1399	2700	1399	3072	3218	1771	1917	2700	1771	3072	1917	3218
Thraupidae	*Diglossa cyanea*	1394	3600	1394	3969	4121	1763	1915	3600	1763	3969	1915	4121
Thraupidae	*Diglossa glauca*	1000	2300	1000	2672	2822	1372	1522	2300	1372	2672	1522	2822
Thraupidae	*Diglossa mystacalis*	2600	3600	2600	3964	4126	2964	3126	3600	2964	3964	3126	4126
Thraupidae	*Diglossa sittoides*	1900	3600	1900	3968	4122	2268	2422	3600	2268	3968	2422	4122
Thraupidae	*Dubusia taeniata*	1900	3200	1900	3572	3723	2272	2423	3200	2272	3572	2423	3723
Thraupidae	*Euphonia chlorotica*	250	450	250	852	1029	652	829	450	652	852	829	1029
Thraupidae	*Euphonia chrysopasta*	250	950	250	1344	1513	644	813	950	644	1344	813	1513
Thraupidae	*Euphonia laniirostris*	250	1500	250	1883	2044	633	794	1500	633	1883	794	2044
Thraupidae	*Euphonia mesochrysa*	600	1800	600	2171	2325	971	1125	1800	971	2171	1125	2325
Thraupidae	*Euphonia minuta*	250	1050	250	1436	1603	636	803	1050	636	1436	803	1603
Thraupidae	*Euphonia rufiventris*	250	950	250	1344	1513	644	813	950	644	1344	813	1513
Thraupidae	*Euphonia xanthogaster*	250	2130	250	2511	2669	631	789	2130	631	2511	789	2669
Thraupidae	*Iridophanes pulcherrimus*	1100	1800	1100	2172	2318	1472	1618	1800	1472	2172	1618	2318
Thraupidae	*Iridosornis analis*	1000	2200	1000	2572	2722	1372	1522	2200	1372	2572	1522	2722
Thraupidae	*Iridosornis jelskii*	2200	3500	2200	3867	4023	2567	2723	3500	2567	3867	2723	4023
Thraupidae	*Lamprospiza melanoleuca*	250	900	250	1294	1463	644	813	900	644	1294	813	1463
Thraupidae	*Pipraeidea melanonota*	450	3000	450	3374	3528	824	978	3000	824	3374	978	3528
Thraupidae	*Piranga leucoptera*	900	1800	900	2171	2325	1271	1425	1800	1271	2171	1425	2325
Thraupidae	*Ramphocelus carbo*	250	1600	250	1983	2144	633	794	1600	633	1983	794	2144
Thraupidae	*Ramphocelus nigrogularis*	250	1000	250	1386	1553	636	803	1000	636	1386	803	1553
Thraupidae	*Schistochlamys melanopis*	600	2600	600	2972	3121	972	1121	2600	972	2972	1121	3121
Thraupidae	*Tachyphonus cristatus*	300	450	300	852	1029	702	879	450	702	852	879	1029
Thraupidae	*Tachyphonus rufiventer*	250	1250	250	1636	1803	636	803	1250	636	1636	803	1803
Thraupidae	*Tangara arthus*	600	1800	600	2171	2325	971	1125	1800	971	2171	1125	2325
Thraupidae	*Tangara callophrys*	250	950	250	1344	1513	644	813	950	644	1344	813	1513
Thraupidae	*Tangara chilensis*	250	1921	250	2304	2465	633	794	1921	633	2304	794	2465
Thraupidae	*Tangara chrysotis*	850	2115	850	2487	2637	1222	1372	2115	1222	2487	1372	2637
Thraupidae	*Tangara cyanicollis*	500	2000	500	2375	2529	875	1029	2000	875	2375	1029	2529
Thraupidae	*Tangara cyanotis*	1300	2000	1300	2372	2518	1672	1818	2000	1672	2372	1818	2518
Thraupidae	*Tangara gyrola*	250	1500	250	1883	2044	633	794	1500	633	1883	794	2044
Thraupidae	*Tangara mexicana*	250	1000	250	1386	1553	636	803	1000	636	1386	803	1553
Thraupidae	*Tangara nigrocincta*	250	1100	250	1486	1653	636	803	1100	636	1486	803	1653
Thraupidae	*Tangara nigroviridis*	1220	2500	1220	2872	3018	1592	1738	2500	1592	2872	1738	3018
Thraupidae	*Tangara parzudakii*	1375	2000	1375	2372	2518	1747	1893	2000	1747	2372	1893	2518
Thraupidae	*Tangara punctata*	600	2000	600	2372	2522	972	1122	2000	972	2372	1122	2522
Thraupidae	*Tangara ruficervix*	1000	2100	1000	2472	2622	1372	1522	2100	1372	2472	1522	2622
Thraupidae	*Tangara schrankii*	250	1100	250	1486	1653	636	803	1100	636	1486	803	1653
Thraupidae	*Tangara vassorii*	1690	3250	1690	3622	3773	2062	2213	3250	2062	3622	2213	3773
Thraupidae	*Tangara velia*	250	600	250	994	1163	644	813	600	644	994	813	1163
Thraupidae	*Tangara viridicollis*	800	2600	800	2972	3121	1172	1321	2600	1172	2972	1321	3121
Thraupidae	*Tangara xanthocephala*	1000	2500	1000	2872	3021	1372	1521	2500	1372	2872	1521	3021
Thraupidae	*Tangara xanthogastra*	250	1400	250	1786	1953	636	803	1400	636	1786	803	1953
Thraupidae	*Tersina viridis*	250	1450	250	1836	2003	636	803	1450	636	1836	803	2003
Thraupidae	*Thlypopsis sordida*	250	600	250	994	1163	644	813	600	644	994	813	1163
Thraupidae	*Thraupis bonariensis*	1000	3500	1000	3869	4023	1369	1523	3500	1369	3869	1523	4023
Thraupidae	*Thraupis cyanocephala*	1335	3032	1335	3404	3554	1707	1857	3032	1707	3404	1857	3554
Thraupidae	*Thraupis episcopus*	250	1600	250	1983	2144	633	794	1600	633	1983	794	2144
Thraupidae	*Thraupis palmarum*	250	1600	250	1983	2144	633	794	1600	633	1983	794	2144
Thraupidae	*Trichothraupis melanops*	1000	1650	1000	2021	2175	1371	1525	1650	1371	2021	1525	2175

Tinamidae	*Crypturellus atrocapillus*	250	1000	250	1386	1553	636	803	1000	636	1386	803	1553
Tinamidae	*Crypturellus bartletti*	250	500	250	894	1063	644	813	500	644	894	813	1063
Tinamidae	*Crypturellus cinereus*	250	1000	250	1386	1553	636	803	1000	636	1386	803	1553
Tinamidae	*Crypturellus obsoletus*	450	3013	450	3387	3541	824	978	3013	824	3387	978	3541
Tinamidae	*Crypturellus soui*	250	1500	250	1883	2044	633	794	1500	633	1883	794	2044
Tinamidae	*Crypturellus strigulosus*	350	750	350	1137	1298	737	898	750	737	1137	898	1298
Tinamidae	*Crypturellus undulatus*	250	800	250	1194	1363	644	813	800	644	1194	813	1363
Tinamidae	*Crypturellus variegatus*	250	500	250	894	1063	644	813	500	644	894	813	1063
Tinamidae	*Nothocercus nigrocapillus*	1600	3200	1600	3572	3723	1972	2123	3200	1972	3572	2123	3723
Tinamidae	*Tinamus guttatus*	250	1100	250	1486	1653	636	803	1100	636	1486	803	1653
Tinamidae	*Tinamus major*	250	700	250	1094	1263	644	813	700	644	1094	813	1263
Tinamidae	*Tinamus tao*	250	1300	250	1686	1853	636	803	1300	636	1686	803	1853
Trogonidae	*Pharomachrus antisianus*	1000	2338	1000	2710	2860	1372	1522	2338	1372	2710	1522	2860
Trogonidae	*Pharomachrus auriceps*	1300	3049	1300	3421	3571	1672	1822	3049	1672	3421	1822	3571
Trogonidae	*Pharomachrus pavoninus*	250	1200	250	1586	1753	636	803	1200	636	1586	803	1753
Trogonidae	*Trogon collaris*	250	1489	250	1875	2042	636	803	1489	636	1875	803	2042
Trogonidae	*Trogon curucui*	250	1500	250	1883	2044	633	794	1500	633	1883	794	2044
Trogonidae	*Trogon melanurus*	250	1000	250	1386	1553	636	803	1000	636	1386	803	1553
Trogonidae	*Trogon personatus*	1100	3500	1100	3869	4021	1469	1621	3500	1469	3869	1621	4021
Trogonidae	*Trogon violaceus*	250	876	250	1270	1439	644	813	876	644	1270	813	1439
Trogonidae	*Trogon viridis*	250	878	250	1272	1441	644	813	878	644	1272	813	1441
Turdidae	*Catharus fuscater*	1500	2850	1500	3222	3368	1872	2018	2850	1872	3222	2018	3368
Turdidae	*Entomodestes leucotis*	1139	2900	1139	3272	3418	1511	1657	2900	1511	3272	1657	3418
Turdidae	*Myadestes ralloides*	750	2900	750	3272	3421	1122	1271	2900	1122	3272	1271	3421
Turdidae	*Turdus albicollis*	250	850	250	1244	1413	644	813	850	644	1244	813	1413
Turdidae	*Turdus chiguanco*	1300	3520	1300	3889	4041	1669	1821	3520	1669	3889	1821	4041
Turdidae	*Turdus fuscater*	2500	3500	2500	3867	4023	2867	3023	3500	2867	3867	3023	4023
Turdidae	*Turdus hauxwelli*	250	1496	250	1882	2049	636	803	1496	636	1882	803	2049
Turdidae	*Turdus ignobilis*	250	1640	250	2023	2184	633	794	1640	633	2023	794	2184
Turdidae	*Turdus leucops*	850	2600	850	2972	3121	1222	1371	2600	1222	2972	1371	3121
Turdidae	*Turdus nigriceps*	250	1851	250	2234	2395	633	794	1851	633	2234	794	2395
Turdidae	*Turdus serranus*	1400	3342	1400	3714	3864	1772	1922	3342	1772	3714	1922	3864
Tyrannidae	*Elaenia albiceps*	500	3250	500	3624	3778	874	1028	3250	874	3624	1028	3778
Tyrannidae	*Elaenia flavogaster*	450	900	450	1287	1448	837	998	900	837	1287	998	1448
Tyrannidae	*Elaenia gigas*	250	1550	250	1933	2094	633	794	1550	633	1933	794	2094
Tyrannidae	*Elaenia obscura*	1700	3000	1700	3372	3523	2072	2223	3000	2072	3372	2223	3523
Tyrannidae	*Elaenia pallatangae*	1100	3250	1100	3622	3772	1472	1622	3250	1472	3622	1622	3772
Tyrannidae	*Lophotriccus pileatus*	800	1821	800	2192	2346	1171	1325	1821	1171	2192	1325	2346
Tyrannidae	*Mionectes macconnelli*	250	1200	250	1586	1753	636	803	1200	636	1586	803	1753
Tyrannidae	*Mionectes oleagineus*	250	1350	250	1736	1903	636	803	1350	636	1736	803	1903
Tyrannidae	*Mionectes olivaceus*	250	1712	250	2095	2256	633	794	1712	633	2095	794	2256
Tyrannidae	*Mionectes striaticollis*	550	3377	550	3749	3901	922	1074	3377	922	3749	1074	3901
Tyrannidae	*Zimmerius bolivianus*	1000	2901	1000	3273	3422	1372	1521	2901	1372	3273	1521	3422
Tyrannidae	*Zimmerius gracilipes*	250	850	250	1244	1413	644	813	850	644	1244	813	1413

Table S2. Current and future probabilities of occurrence derived from species distribution models (SDMs) of all 232 frugivorous bird species of the Manú Biosphere Reserve (Peru) based on the MIROC5 climate model. SDMs were based on current and future local temperature and precipitation values.

		500 m			1000 m			1500 m			2000 m			2500 m			3000 m			3500 m		
Family	Bird species	Current	RCP 6.0	RCP 8.5	Current	RCP 6.0	RCP 8.5	Current	RCP 6.0	RCP 8.5	Current	RCP 6.0	RCP 8.5	Current	RCP 6.0	RCP 8.5	Current	RCP 6.0	RCP 8.5	Current	RCP 6.0	RCP 8.5
Cardinalidae	*Cyanocompsa cyanoides*	0.354	0.936	0.980	0.965	0.975	0.990	0.268	0.968	0.898	0.819	0.758	0.981	0.012	0.093	0.577	0.011	0.049	0.006	0.003	0.004	0.003
Cardinalidae	*Pheucticus aureoventris*	0.398	0.008	0.008	0.302	0.027	0.597	0.008	0.183	0.163	0.028	0.321	0.232	0.007	0.013	0.008	0.043	0.007	0.063	0.006	0.003	0.004
Cardinalidae	*Pheucticus chrysogaster*	0.003	0.003	0.003	0.063	0.011	0.215	0.476	0.179	0.005	0.520	0.054	0.036	0.004	0.052	0.244	0.004	0.064	0.605	0.010	0.004	0.004
Cardinalidae	*Saltator aurantiirostris*	0.003	0.002	0.002	0.005	0.002	0.002	0.003	0.022	0.002	0.003	0.007	0.003	0.002	0.002	0.002	0.002	0.002	0.002	0.003	0.004	0.004
Cardinalidae	*Saltator coerulescens*	0.023	0.013	0.072	0.323	0.848	0.566	0.090	0.683	0.938	0.059	0.582	0.137	0.004	0.586	0.166	0.004	0.004	0.004	0.004	0.004	0.004
Cardinalidae	*Saltator grossus*	0.195	0.553	0.886	0.988	0.841	0.976	0.059	0.505	0.963	0.083	0.830	0.621	0.011	0.030	0.016	0.002	0.003	0.065	0.002	0.003	0.003
Cardinalidae	*Saltator maximus*	0.514	0.452	0.729	0.903	0.424	0.962	0.136	0.958	0.955	0.941	0.634	0.756	0.007	0.168	0.705	0.006	0.006	0.039	0.005	0.005	0.007
Columbidae	*Geotrygon montana*	0.731	0.286	0.893	0.270	0.567	0.880	0.633	0.911	0.819	0.727	0.517	0.831	0.007	0.041	0.267	0.005	0.125	0.002	0.001	0.001	0.005
Columbidae	*Patagioenas cayennensis*	0.067	0.013	0.730	0.753	0.466	0.045	0.029	0.956	0.989	0.737	0.045	0.717	0.005	0.008	0.737	0.005	0.085	0.100	0.005	0.005	0.004
Columbidae	*Patagioenas fasciata*	0.016	0.010	0.007	0.962	0.432	0.003	0.790	0.496	0.167	0.993	0.899	0.326	0.969	0.796	0.899	0.090	0.296	0.457	0.005	0.170	0.004
Columbidae	*Patagioenas plumbea*	0.075	0.188	0.088	0.344	0.272	0.768	0.030	0.978	0.991	0.679	0.923	0.899	0.228	0.102	0.838	0.003	0.225	0.408	0.003	0.003	0.031
Columbidae	*Patagioenas subvinacea*	0.008	0.020	0.136	0.593	0.955	0.980	0.698	0.792	0.967	0.930	0.719	0.758	0.326	0.868	0.964	0.015	0.023	0.100	0.007	0.008	0.007
Corvidae	*Cyanocorax cyanomelas*	0.338	0.004	0.004	0.496	0.701	0.972	0.010	0.254	0.761	0.003	0.044	0.260	0.003	0.003	0.003	0.004	0.003	0.003	0.003	0.003	0.003
Corvidae	*Cyanocorax violaceus*	0.544	0.005	0.278	0.207	0.971	0.991	0.132	0.484	0.955	0.004	0.089	0.599	0.004	0.039	0.067	0.003	0.003	0.003	0.009	0.003	0.003
Corvidae	*Cyanocorax yncas*	0.005	0.009	0.008	0.974	0.660	0.007	0.453	0.402	0.035	0.994	0.992	0.776	0.885	0.994	0.996	0.101	0.547	0.552	0.004	0.044	0.004
Cotingidae	*Ampelioides tschudii*	0.517	0.080	0.080	0.765	0.977	0.790	0.994	0.951	0.954	0.993	0.918	0.854	0.028	0.255	0.457	0.026	0.048	0.444	0.022	0.010	0.013
Cotingidae	*Ampelion rubrocristatus*	0.007	0.005	0.005	0.072	0.004	0.014	0.142	0.014	0.176	0.453	0.090	0.085	0.903	0.939	0.255	0.015	0.291	0.453	0.721	0.068	0.119
Cotingidae	*Ampelion rufaxilla*	0.027	0.024	0.024	0.918	0.221	0.036	0.958	0.941	0.352	0.953	0.890	0.554	0.856	0.884	0.933	0.863	0.940	0.912	0.070	0.071	0.082
Cotingidae	*Cephalopterus ornatus*	0.371	0.175	0.659	0.044	0.842	0.935	0.534	0.792	0.681	0.538	0.047	0.681	0.018	0.084	0.215	0.005	0.004	0.004	0.004	0.004	0.005
Cotingidae	*Conioptilon mcilhennyi*	0.222	0.014	0.001	0.057	0.211	0.431	0.073	0.063	0.094	0.078	0.070	0.069	0.084	0.078	0.077	0.049	0.062	0.063	0.089	0.089	0.088
Cotingidae	*Cotinga cayana*	0.070	0.064	0.024	0.128	0.667	0.589	0.050	0.163	0.218	0.045	0.064	0.166	0.036	0.033	0.037	0.003	0.005	0.005	0.004	0.007	0.008
Cotingidae	*Cotinga maynana*	0.023	0.003	0.000	0.271	0.962	0.994	0.000	0.057	0.338	0.000	0.003	0.000	0.000	0.000	0.000	0.000	0.000	0.000	0.000	0.000	0.000
Cotingidae	*Gymnoderus foetidus*	0.010	0.010	0.230	0.003	0.006	0.963	0.002	0.002	0.002	0.002	0.003	0.002	0.003	0.002	0.002	0.002	0.010	0.002	0.003	0.002	0.002
Cotingidae	*Iodopleura isabellae*	0.842	0.116	0.375	0.010	0.920	0.990	0.015	0.014	0.498	0.002	0.003	0.005	0.006	0.002	0.002	0.001	0.002	0.002	0.003	0.001	0.001
Cotingidae	*Laniocera hypopyrra*	0.016	0.015	0.015	0.016	0.691	0.890	0.015	0.019	0.126	0.015	0.015	0.023	0.015	0.015	0.015	0.015	0.015	0.015	0.015	0.015	0.015
Cotingidae	*Lipaugus vociferans*	0.041	0.009	0.012	0.002	0.045	0.984	0.002	0.002	0.006	0.002	0.002	0.018	0.002	0.002	0.002	0.002	0.002	0.002	0.002	0.006	0.003
Cotingidae	*Oxyruncus cristatus*	0.172	0.155	0.059	0.729	0.618	0.171	0.781	0.547	0.267	0.717	0.809	0.641	0.065	0.538	0.754	0.109	0.520	0.886	0.023	0.019	0.019
Cotingidae	*Pipreola arcuata*	0.001	0.001	0.001	0.053	0.029	0.006	0.499	0.114	0.010	0.906	0.043	0.022	0.612	0.915	0.999	0.356	0.932	0.965	0.189	0.992	0.193
Cotingidae	*Pipreola frontalis*	0.601	0.036	0.025	0.926	0.865	0.712	0.913	0.921	0.906	0.888	0.922	0.916	0.034	0.508	0.770	0.024	0.072	0.255	0.023	0.023	0.023
Cotingidae	*Pipreola intermedia*	0.003	0.003	0.003	0.010	0.009	0.003	0.036	0.010	0.010	0.054	0.030	0.007	0.095	0.037	0.051	0.302	0.969	0.988	0.029	0.055	0.124
Cotingidae	*Porphyrolaema porphyrolaema*	0.086	0.049	0.022	0.120	0.491	0.664	0.012	0.120	0.231	0.005	0.047	0.098	0.000	0.001	0.002	0.000	0.000	0.000	0.000	0.000	0.000
Cotingidae	*Querula purpurata*	0.021	0.030	0.011	0.436	0.932	0.944	0.015	0.950	0.950	0.024	0.573	0.882	0.001	0.001	0.004	0.001	0.003	0.003	0.001	0.001	0.001
Cotingidae	*Rupicola peruvianus*	0.029	0.003	0.003	0.996	0.766	0.311	0.995	0.998	0.930	0.909	0.988	0.995	0.733	0.946	0.998	0.004	0.006	0.087	0.006	0.213	0.003
Cotingidae	*Snowornis subalaris*	0.010	0.010	0.010	0.976	0.956	0.970	0.027	0.969	0.945	0.025	0.920	0.921	0.010	0.010	0.864	0.010	0.010	0.010	0.010	0.010	0.010
Cotingidae	*Tityra cayana*	0.009	0.027	0.077	0.024	0.063	0.874	0.004	0.036	0.332	0.004	0.004	0.220	0.048	0.006	0.033	0.002	0.008	0.011	0.002	0.002	0.002
Cotingidae	*Tityra inquisitor*	0.856	0.061	0.462	0.757	0.669	0.706	0.783	0.951	0.966	0.928	0.508	0.986	0.008	0.014	0.101	0.003	0.013	0.114	0.004	0.003	0.003
Cotingidae	*Tityra semifasciata*	0.424	0.600	0.867	0.967	0.968	0.976	0.984	0.913	0.990	0.982	0.669	0.708	0.016	0.806	0.835	0.005	0.006	0.066	0.006	0.007	0.007
Cracidae	*Aburria aburri*	0.838	0.017	0.016	0.985	0.988	0.996	0.995	0.999	0.931	0.956	0.988	0.998	0.043	0.733	0.979	0.033	0.084	0.125	0.009	0.009	0.009
Cracidae	*Mitu tuberosum*	0.903	0.034	0.336	0.044	0.972	0.993	0.001	0.028	0.192	0.001	0.002	0.010	0.001	0.001	0.001	0.001	0.001	0.001	0.001	0.001	0.001
Cracidae	*Ortalis guttata*	0.577	0.006	0.020	0.978	0.769	0.649	0.136	0.968	0.990	0.672	0.743	0.974	0.014	0.232	0.772	0.003	0.089	0.006	0.003	0.005	0.003

Cracidae	*Penelope jacquacu*	0.078	0.006	0.022	0.139	0.130	0.988	0.004	0.337	0.821	0.003	0.048	0.189	0.003	0.003	0.003	0.002	0.003	0.004	0.002	0.003	0.003
Cracidae	*Penelope montagnii*	0.007	0.007	0.007	0.224	0.014	0.008	0.109	0.123	0.588	0.491	0.686	0.036	0.624	0.877	0.982	0.151	0.705	0.906	0.458	0.033	0.007
Cracidae	*Pipile cumanensis*	0.113	0.012	0.024	0.020	0.948	0.983	0.005	0.006	0.493	0.006	0.005	0.006	0.028	0.005	0.005	0.005	0.005	0.005	0.005	0.007	0.006
Icteridae	*Cacicus cela*	0.789	0.023	0.071	0.008	0.700	0.868	0.006	0.014	0.189	0.010	0.013	0.711	0.005	0.299	0.178	0.005	0.005	0.006	0.005	0.005	0.005
Icteridae	*Cacicus chrysonotus*	0.001	0.002	0.002	0.683	0.077	0.005	0.133	0.700	0.628	0.632	0.119	0.287	0.874	0.957	0.996	0.295	0.058	0.084	0.040	0.163	0.004
Icteridae	*Cacicus haemorrhous*	0.006	0.206	0.040	0.007	0.022	0.041	0.005	0.028	0.067	0.003	0.003	0.114	0.031	0.014	0.016	0.004	0.013	0.035	0.003	0.003	0.003
Icteridae	*Clypicterus oseryi*	0.988	0.058	0.026	0.169	0.195	0.996	0.023	0.101	0.192	0.030	0.036	0.066	0.005	0.018	0.148	0.002	0.002	0.002	0.010	0.006	0.005
Icteridae	*Icterus icterus*	0.074	0.028	0.484	0.054	0.040	0.080	0.035	0.042	0.040	0.035	0.066	0.049	0.099	0.030	0.031	0.021	0.025	0.025	0.022	0.024	0.024
Icteridae	*Molothrus oryzivorus*	0.216	0.115	0.059	0.673	0.896	0.778	0.095	0.400	0.258	0.637	0.885	0.905	0.009	0.246	0.607	0.007	0.016	0.144	0.006	0.045	0.008
Icteridae	*Psarocolius angustifrons*	0.244	0.009	0.005	0.493	0.813	0.981	0.073	0.784	0.943	0.707	0.954	0.954	0.379	0.868	0.920	0.018	0.008	0.015	0.005	0.005	0.007
Icteridae	*Psarocolius atrovirens*	0.016	0.002	0.002	0.699	0.707	0.012	0.775	0.981	0.546	0.551	0.922	0.982	0.009	0.208	0.222	0.005	0.015	0.099	0.001	0.001	0.001
Icteridae	*Psarocolius bifasciatus*	0.932	0.066	0.483	0.241	0.346	0.804	0.002	0.807	0.518	0.002	0.016	0.862	0.002	0.002	0.006	0.002	0.002	0.002	0.003	0.002	0.002
Icteridae	*Psarocolius decumanus*	0.892	0.499	0.262	0.978	0.653	0.786	0.404	0.943	0.408	0.005	0.914	0.933	0.014	0.574	0.050	0.004	0.026	0.006	0.004	0.004	0.004
Odontophoridae	*Odontophorus balliviani*	0.043	0.040	0.040	0.148	0.030	0.005	0.956	0.065	0.090	0.955	0.420	0.062	0.475	0.631	0.406	0.411	0.568	0.658	0.003	0.004	0.014
Odontophoridae	*Odontophorus speciosus*	0.040	0.007	0.007	0.981	0.064	0.031	0.828	0.986	0.981	0.418	0.968	0.988	0.110	0.359	0.537	0.103	0.136	0.074	0.007	0.009	0.009
Odontophoridae	*Odontophorus stellatus*	0.016	0.008	0.003	0.253	0.431	0.998	0.102	0.253	0.253	0.100	0.170	0.134	0.008	0.039	0.039	0.003	0.001	0.001	0.002	0.001	0.001
Picidae	*Celeus elegans*	0.109	0.031	0.058	0.005	0.008	0.981	0.001	0.003	0.003	0.001	0.003	0.003	0.002	0.001	0.001	0.001	0.001	0.001	0.001	0.001	0.001
Picidae	*Celeus flavus*	0.201	0.173	0.158	0.011	0.107	0.827	0.008	0.010	0.018	0.008	0.008	0.008	0.008	0.008	0.008	0.008	0.008	0.009	0.008	0.008	0.008
Picidae	*Celeus grammicus*	0.791	0.621	0.408	0.030	0.138	0.993	0.008	0.033	0.093	0.007	0.016	0.038	0.026	0.007	0.007	0.006	0.006	0.006	0.006	0.016	0.014
Picidae	*Celeus spectabilis*	0.201	0.000	0.000	0.303	0.570	1.000	0.174	0.303	0.315	0.183	0.187	0.257	0.000	0.206	0.211	0.002	0.000	0.000	0.000	0.001	0.001
Picidae	*Celeus torquatus*	0.190	0.152	0.226	0.010	0.016	0.334	0.011	0.010	0.012	0.012	0.011	0.011	0.011	0.012	0.012	0.010	0.009	0.009	0.010	0.011	0.011
Picidae	*Colaptes rubiginosus*	0.274	0.005	0.005	0.985	0.804	0.603	0.995	0.978	0.980	0.986	0.821	0.950	0.094	0.954	0.973	0.018	0.027	0.365	0.005	0.005	0.005
Pipridae	*Chiroxiphia boliviana*	0.117	0.007	0.007	0.142	0.121	0.135	0.123	0.883	0.905	0.028	0.198	0.881	0.009	0.012	0.005	0.004	0.008	0.153	0.004	0.006	0.005
Pipridae	*Chiroxiphia pareola*	0.463	0.236	0.150	0.104	0.771	0.923	0.025	0.085	0.314	0.029	0.043	0.041	0.030	0.029	0.029	0.033	0.032	0.032	0.033	0.027	0.027
Pipridae	*Lepidothrix coeruleocapilla*	0.724	0.007	0.007	0.972	0.448	0.556	0.044	0.993	0.984	0.004	0.573	0.951	0.003	0.004	0.004	0.004	0.004	0.004	0.009	0.003	0.003
Pipridae	*Lepidothrix coronata*	0.825	0.579	0.187	0.207	0.774	0.969	0.014	0.465	0.672	0.014	0.035	0.213	0.012	0.013	0.013	0.010	0.011	0.011	0.013	0.013	0.012
Pipridae	*Machaeropterus pyrocephalus*	0.074	0.005	0.004	0.240	0.984	0.986	0.006	0.064	0.064	0.004	0.023	0.121	0.004	0.004	0.004	0.004	0.004	0.004	0.004	0.004	0.004
Pipridae	*Manacus manacus*	0.895	0.495	0.951	0.830	0.908	0.958	0.193	0.930	0.994	0.984	0.835	0.442	0.014	0.963	0.905	0.001	0.002	0.013	0.002	0.002	0.001
Pipridae	*Neopelma sulphureiventer*	0.311	0.137	0.083	0.050	0.077	0.130	0.031	0.035	0.039	0.032	0.037	0.037	0.038	0.031	0.031	0.023	0.042	0.042	0.040	0.056	0.056
Pipridae	*Pipra chloromeros*	0.409	0.013	0.013	0.851	0.925	0.762	0.024	0.783	0.386	0.007	0.432	0.740	0.005	0.006	0.007	0.010	0.006	0.006	0.005	0.006	0.006
Pipridae	*Pipra fasciicauda*	0.005	0.003	0.003	0.413	0.804	0.985	0.003	0.641	0.679	0.003	0.006	0.318	0.003	0.003	0.003	0.003	0.003	0.003	0.003	0.003	0.003
Pipridae	*Xenopipo holochlora*	0.983	0.262	0.262	0.799	0.990	0.990	0.064	0.960	0.881	0.018	0.812	0.967	0.001	0.015	0.022	0.021	0.021	0.379	0.001	0.001	0.001
Pipridae	*Xenopipo unicolor*	0.007	0.002	0.002	0.500	0.133	0.056	0.916	0.837	0.810	0.137	0.475	0.875	0.047	0.317	0.247	0.002	0.012	0.075	0.003	0.003	0.003
Psittacidae	*Amazona farinosa*	0.033	0.071	0.573	0.034	0.302	0.987	0.011	0.344	0.971	0.227	0.009	0.055	0.007	0.011	0.366	0.004	0.010	0.009	0.004	0.004	0.010
Psittacidae	*Amazona mercenaria*	0.315	0.028	0.028	0.974	0.659	0.511	0.974	0.970	0.811	0.977	0.976	0.973	0.981	0.981	0.980	0.948	0.759	0.809	0.910	0.838	0.843
Psittacidae	*Amazona ochrocephala*	0.009	0.018	0.314	0.015	0.194	0.937	0.010	0.018	0.043	0.006	0.012	0.009	0.023	0.004	0.005	0.004	0.009	0.005	0.004	0.005	0.008
Psittacidae	*Ara ararauna*	0.506	0.034	0.070	0.010	0.098	0.939	0.005	0.070	0.012	0.005	0.022	0.027	0.004	0.005	0.005	0.005	0.005	0.004	0.005	0.005	0.005
Psittacidae	*Ara chloropterus*	0.079	0.033	0.194	0.004	0.063	0.847	0.008	0.007	0.016	0.003	0.019	0.006	0.003	0.003	0.003	0.003	0.005	0.004	0.004	0.005	0.003
Psittacidae	*Ara macao*	0.007	0.020	0.022	0.070	0.035	0.127	0.008	0.077	0.097	0.003	0.042	0.110	0.004	0.003	0.003	0.003	0.003	0.003	0.003	0.003	0.003
Psittacidae	*Ara militaris*	0.157	0.045	0.051	0.994	0.971	0.929	0.747	0.994	0.995	0.809	0.587	0.761	0.004	0.213	0.619	0.001	0.001	0.006	0.001	0.001	0.001
Psittacidae	*Ara severus*	0.114	0.712	0.945	0.120	0.102	0.990	0.012	0.862	0.991	0.007	0.015	0.061	0.012	0.044	0.020	0.004	0.006	0.005	0.004	0.004	0.010
Psittacidae	*Aratinga leucophthalma*	0.019	0.011	0.011	0.021	0.181	0.832	0.006	0.149	0.165	0.003	0.007	0.195	0.107	0.003	0.013	0.003	0.130	0.133	0.003	0.004	0.004
Psittacidae	*Aratinga mitrata*	0.004	0.001	0.001	0.003	0.001	0.001	0.001	0.004	0.016	0.001	0.008	0.003	0.002	0.002	0.001	0.002	0.002	0.005	0.002	0.002	0.001
Psittacidae	*Aratinga weddellii*	0.179	0.015	0.003	0.283	0.569	0.877	0.006	0.105	0.156	0.002	0.004	0.030	0.002	0.002	0.002	0.003	0.002	0.002	0.003	0.002	0.002
Psittacidae	*Bolborhynchus lineola*	0.176	0.042	0.022	0.475	0.118	0.061	0.766	0.609	0.179	0.800	0.632	0.414	0.715	0.752	0.803	0.195	0.566	0.564	0.133	0.157	0.167
Psittacidae	*Bolborhynchus orbygnesius*	0.011	0.011	0.011	0.056	0.014	0.011	0.344	0.050	0.049	0.234	0.067	0.049	0.049	0.107	0.112	0.029	0.051	0.058	0.012	0.015	0.016
Psittacidae	*Brotogeris cyanoptera*	0.052	0.007	0.147	0.002	0.154	0.983	0.002	0.010	0.004	0.002	0.002	0.085	0.002	0.007	0.002	0.002	0.002	0.002	0.002	0.002	0.002
Psittacidae	*Brotogeris sanctithomae*	0.041	0.184	0.086	0.059	0.059	0.287	0.031	0.059	0.059	0.028	0.031	0.055	0.028	0.028	0.031	0.002	0.002	0.002	0.009	0.028	0.028
Psittacidae	*Forpus modestus*	0.017	0.017	0.017	0.256	0.348	0.801	0.018	0.360	0.360	0.017	0.129	0.350	0.017	0.017	0.017	0.017	0.017	0.017	0.017	0.017	0.017
Psittacidae	*Nannopsittaca dachilleae*	0.263	0.992	0.992	0.009	0.010	0.249	0.001	0.016	0.019	0.001	0.002	0.002	0.000	0.001	0.001	0.000	0.000	0.000	0.000	0.000	0.000

Psittacidae	*Orthopsittaca manilata*	0.098	0.007	0.007	0.092	0.221	0.957	0.005	0.019	0.019	0.003	0.005	0.003	0.015	0.023	0.003	0.003	0.004	0.003	0.004	0.007	0.010
Psittacidae	*Pionites leucogaster*	0.004	0.000	0.000	0.000	0.000	0.241	0.000	0.000	0.000	0.000	0.000	0.000	0.001	0.001	0.000	0.000	0.000	0.000	0.000	0.000	0.000
Psittacidae	*Pionus menstruus*	0.137	0.161	0.542	0.489	0.518	0.981	0.078	0.546	0.762	0.653	0.039	0.050	0.004	0.092	0.867	0.004	0.004	0.004	0.004	0.004	0.004
Psittacidae	*Pionus tumultuosus*	0.073	0.041	0.040	0.203	0.048	0.009	0.959	0.029	0.418	0.989	0.076	0.024	0.893	0.990	0.993	0.297	0.990	0.991	0.042	0.029	0.212
Psittacidae	*Primolius couloni*	0.109	0.006	0.006	0.050	0.975	0.991	0.006	0.020	0.042	0.007	0.029	0.008	0.005	0.004	0.005	0.015	0.011	0.012	0.026	0.006	0.006
Psittacidae	*Pyrilia barrabandi*	0.006	0.006	0.006	0.006	0.007	0.030	0.006	0.006	0.642	0.006	0.006	0.006	0.006	0.006	0.006	0.006	0.006	0.006	0.006	0.006	0.006
Psittacidae	*Pyrrhura roseifrons*	0.005	0.005	0.005	0.005	0.646	0.941	0.005	0.005	0.104	0.005	0.005	0.005	0.005	0.005	0.005	0.005	0.005	0.005	0.005	0.005	0.005
Psittacidae	*Pyrrhura rupicola*	0.018	0.018	0.018	0.433	0.508	0.890	0.261	0.226	0.202	0.823	0.213	0.226	0.018	0.018	0.572	0.018	0.018	0.018	0.018	0.018	0.018
Psittacidae	*Touit huetii*	0.264	0.318	0.062	0.005	0.018	0.941	0.001	0.007	0.012	0.001	0.005	0.007	0.001	0.001	0.001	0.000	0.000	0.000	0.001	0.000	0.000
Psophiidae	*Psophia leucoptera*	0.995	0.274	0.159	0.057	0.563	0.996	0.023	0.061	0.313	0.023	0.028	0.146	0.012	0.022	0.021	0.002	0.002	0.002	0.007	0.006	0.012
Ramphastidae	*Andigena hypoglauca*	0.003	0.003	0.003	0.003	0.003	0.003	0.017	0.003	0.003	0.008	0.003	0.003	0.230	0.047	0.026	0.767	0.928	0.688	0.814	0.200	0.147
Ramphastidae	*Aulacorhynchus coeruleicinctis*	0.009	0.009	0.009	0.837	0.010	0.009	0.915	0.871	0.635	0.707	0.928	0.863	0.043	0.671	0.520	0.236	0.885	0.900	0.012	0.012	0.010
Ramphastidae	*Aulacorhynchus derbianus*	0.974	0.359	0.240	0.984	0.984	0.982	0.705	0.981	0.983	0.348	0.949	0.912	0.110	0.425	0.451	0.028	0.167	0.657	0.030	0.019	0.019
Ramphastidae	*Aulacorhynchus prasinus*	0.116	0.001	0.001	0.937	0.976	0.986	0.948	0.841	0.898	0.995	0.766	0.194	0.860	0.915	0.956	0.047	0.522	0.419	0.060	0.316	0.240
Ramphastidae	*Capito auratus*	0.079	0.008	0.012	0.018	0.143	0.880	0.002	0.014	0.568	0.001	0.010	0.003	0.001	0.019	0.008	0.001	0.001	0.001	0.001	0.002	0.002
Ramphastidae	*Eubucco richardsoni*	0.961	0.012	0.010	0.376	0.949	0.986	0.003	0.582	0.966	0.002	0.039	0.750	0.003	0.002	0.002	0.002	0.003	0.003	0.006	0.002	0.002
Ramphastidae	*Eubucco tucinkae*	0.602	0.073	0.073	0.310	0.997	0.998	0.100	0.112	0.112	0.086	0.086	0.086	0.007	0.086	0.098	0.004	0.004	0.004	0.005	0.005	0.005
Ramphastidae	*Eubucco versicolor*	0.742	0.246	0.246	0.909	0.167	0.003	0.063	0.849	0.924	0.004	0.753	0.570	0.002	0.007	0.010	0.001	0.050	0.628	0.002	0.002	0.002
Ramphastidae	*Pteroglossus azara*	0.887	0.075	0.093	0.034	0.976	0.971	0.008	0.013	0.340	0.001	0.012	0.009	0.002	0.001	0.002	0.001	0.001	0.001	0.001	0.002	0.002
Ramphastidae	*Pteroglossus beauharnaesii*	0.096	0.015	0.024	0.007	0.236	0.875	0.004	0.005	0.009	0.004	0.004	0.005	0.003	0.003	0.004	0.003	0.003	0.003	0.003	0.003	0.003
Ramphastidae	*Pteroglossus castanotis*	0.645	0.017	0.029	0.135	0.414	0.920	0.021	0.093	0.277	0.012	0.050	0.053	0.005	0.033	0.005	0.004	0.004	0.016	0.004	0.004	0.004
Ramphastidae	*Pteroglossus inscriptus*	0.384	0.037	0.023	0.026	0.829	0.901	0.015	0.027	0.046	0.014	0.021	0.024	0.013	0.014	0.014	0.015	0.014	0.014	0.014	0.013	0.013
Ramphastidae	*Ramphastos tucanus*	0.007	0.015	0.034	0.004	0.012	0.986	0.002	0.040	0.286	0.002	0.179	0.022	0.003	0.002	0.002	0.002	0.002	0.002	0.003	0.003	0.002
Ramphastidae	*Ramphastos vitellinus*	0.204	0.204	0.460	0.013	0.283	0.894	0.005	0.009	0.419	0.004	0.122	0.032	0.232	0.009	0.038	0.003	0.003	0.003	0.003	0.004	0.003
Ramphastidae	*Selenidera reinwardtii*	0.909	0.084	0.005	0.154	0.686	0.849	0.001	0.064	0.835	0.001	0.007	0.019	0.001	0.003	0.001	0.000	0.000	0.000	0.000	0.000	0.000
Steatornithidae	*Steatornis caripensis*	0.992	0.056	0.264	0.988	0.966	0.991	0.957	0.818	0.964	0.994	0.983	0.956	0.854	0.989	0.987	0.954	0.798	0.795	0.011	0.130	0.795
Thraupidae	*Anisognathus igniventris*	0.003	0.003	0.003	0.176	0.203	0.044	0.392	0.185	0.375	0.529	0.018	0.008	0.090	0.222	0.966	0.551	0.940	0.993	0.878	0.767	0.005
Thraupidae	*Anisognathus somptuosus*	0.008	0.004	0.004	0.998	0.052	0.057	0.684	0.871	0.045	0.998	0.414	0.543	0.130	0.831	0.894	0.051	0.009	0.400	0.023	0.258	0.004
Thraupidae	*Buthraupis montana*	0.010	0.006	0.006	0.059	0.232	0.083	0.819	0.154	0.263	0.862	0.006	0.005	0.370	0.864	0.990	0.040	0.232	0.807	0.023	0.509	0.078
Thraupidae	*Catamblyrhynchus diadema*	0.010	0.010	0.010	0.191	0.031	0.010	0.389	0.129	0.083	0.862	0.119	0.066	0.921	0.940	0.942	0.864	0.945	0.953	0.239	0.612	0.571
Thraupidae	*Chlorochrysa calliparaea*	0.593	0.007	0.007	0.876	0.745	0.967	0.585	0.925	0.819	0.988	0.119	0.614	0.065	0.717	0.996	0.005	0.063	0.015	0.005	0.005	0.005
Thraupidae	*Chlorophanes spiza*	0.924	0.501	0.319	0.960	0.930	0.966	0.829	0.514	0.964	0.712	0.935	0.988	0.020	0.089	0.186	0.004	0.005	0.004	0.003	0.006	0.005
Thraupidae	*Chlorophonia cyanea*	0.051	0.004	0.004	0.913	0.339	0.199	0.993	0.904	0.978	0.335	0.900	0.890	0.637	0.823	0.599	0.004	0.045	0.811	0.004	0.007	0.006
Thraupidae	*Chlorornis riefferii*	0.007	0.003	0.003	0.782	0.323	0.367	0.331	0.798	0.505	0.924	0.658	0.522	0.813	0.884	0.995	0.561	0.991	0.993	0.039	0.015	0.009
Thraupidae	*Chlorospingus canigularis*	0.008	0.006	0.006	0.970	0.444	0.006	0.881	0.812	0.610	0.922	0.916	0.826	0.371	0.960	0.995	0.098	0.773	0.469	0.006	0.005	0.006
Thraupidae	*Chlorospingus flavigularis*	0.215	0.010	0.010	0.985	0.662	0.666	0.995	0.992	0.996	0.985	0.994	0.994	0.059	0.076	0.995	0.007	0.059	0.015	0.003	0.003	0.003
Thraupidae	*Chlorospingus ophthalmicus*	0.216	0.002	0.002	0.974	0.358	0.212	0.129	0.289	0.122	0.166	0.209	0.206	0.776	0.919	0.993	0.005	0.971	0.406	0.002	0.127	0.009
Thraupidae	*Chlorospingus parvirostris*	0.055	0.007	0.007	0.885	0.148	0.056	0.966	0.929	0.859	0.951	0.710	0.778	0.414	0.934	0.971	0.132	0.499	0.762	0.007	0.008	0.143
Thraupidae	*Cissopis leverianus*	0.982	0.020	0.009	0.971	0.965	0.919	0.010	0.856	0.898	0.032	0.030	0.789	0.032	0.225	0.829	0.007	0.011	0.075	0.008	0.006	0.007
Thraupidae	*Conirostrum cinereum*	0.004	0.003	0.003	0.013	0.056	0.003	0.008	0.078	0.005	0.016	0.013	0.068	0.006	0.012	0.008	0.003	0.012	0.337	0.014	0.003	0.003
Thraupidae	*Conirostrum speciosum*	0.016	0.010	0.015	0.182	0.105	0.083	0.084	0.164	0.565	0.193	0.136	0.260	0.006	0.018	0.247	0.007	0.024	0.093	0.006	0.008	0.009
Thraupidae	*Cyanerpes caeruleus*	0.868	0.111	0.693	0.665	0.579	0.928	0.734	0.835	0.987	0.818	0.865	0.856	0.006	0.260	0.233	0.002	0.002	0.002	0.002	0.006	0.006
Thraupidae	*Cyanerpes cyaneus*	0.018	0.107	0.648	0.005	0.083	0.034	0.069	0.007	0.417	0.371	0.013	0.009	0.033	0.389	0.358	0.002	0.000	0.001	0.002	0.000	0.000
Thraupidae	*Dacnis cayana*	0.952	0.736	0.347	0.840	0.838	0.179	0.093	0.216	0.969	0.075	0.492	0.822	0.431	0.030	0.622	0.005	0.015	0.070	0.005	0.005	0.005
Thraupidae	*Dacnis flaviventer*	0.894	0.263	0.035	0.209	0.950	0.993	0.006	0.099	0.374	0.053	0.020	0.018	0.005	0.190	0.086	0.001	0.002	0.005	0.002	0.004	0.003
Thraupidae	*Dacnis lineata*	0.344	0.088	0.033	0.893	0.990	0.402	0.748	0.543	0.979	0.594	0.352	0.969	0.005	0.487	0.731	0.003	0.004	0.135	0.003	0.004	0.004
Thraupidae	*Delothraupis castaneoventris*	0.047	0.004	0.004	0.098	0.032	0.009	0.065	0.240	0.080	0.065	0.130	0.219	0.000	0.001	0.004	0.070	0.080	0.039	0.000	0.007	0.003
Thraupidae	*Diglossa brunneiventris*	0.001	0.001	0.001	0.001	0.001	0.001	0.001	0.001	0.001	0.001	0.001	0.001	0.581	0.002	0.002	0.003	0.006	0.001	0.007	0.063	0.044
Thraupidae	*Diglossa caerulescens*	0.010	0.004	0.005	0.954	0.006	0.004	0.041	0.992	0.132	0.021	0.860	0.622	0.977	0.997	0.998	0.145	0.346	0.813	0.042	0.078	0.127
Thraupidae	*Diglossa cyanea*	0.003	0.002	0.002	0.025	0.090	0.020	0.075	0.015	0.096	0.982	0.147	0.072	0.969	0.280	0.956	0.044	0.653	0.303	0.125	0.042	0.003

Thraupidae	*Diglossa glauca*	0.641	0.049	0.049	0.986	0.882	0.627	0.983	0.986	0.980	0.950	0.985	0.983	0.082	0.673	0.969	0.010	0.073	0.077	0.013	0.018	0.023
Thraupidae	*Diglossa mystacalis*	0.001	0.001	0.001	0.019	0.324	0.001	0.010	0.019	0.047	0.002	0.008	0.013	0.002	0.005	0.002	0.185	0.091	0.097	0.033	0.011	0.003
Thraupidae	*Diglossa sittoides*	0.003	0.002	0.002	0.740	0.018	0.005	0.974	0.659	0.491	0.967	0.921	0.700	0.216	0.711	0.375	0.062	0.682	0.779	0.004	0.005	0.004
Thraupidae	*Dubusia taeniata*	0.001	0.001	0.001	0.003	0.001	0.001	0.182	0.009	0.002	0.106	0.008	0.006	0.863	0.657	0.868	0.374	0.156	0.731	0.239	0.971	0.919
Thraupidae	*Euphonia chlorotica*	0.005	0.019	0.074	0.012	0.070	0.008	0.014	0.011	0.009	0.009	0.024	0.115	0.004	0.009	0.095	0.004	0.011	0.010	0.004	0.004	0.004
Thraupidae	*Euphonia chrysopasta*	0.882	0.062	0.074	0.496	0.918	0.961	0.051	0.459	0.867	0.049	0.318	0.393	0.022	0.042	0.051	0.017	0.017	0.017	0.026	0.028	0.017
Thraupidae	*Euphonia laniirostris*	0.020	0.152	0.188	0.794	0.922	0.863	0.909	0.840	0.824	0.980	0.901	0.782	0.005	0.511	0.307	0.003	0.005	0.135	0.003	0.119	0.003
Thraupidae	*Euphonia mesochrysa*	0.156	0.075	0.075	0.968	0.048	0.034	0.935	0.981	0.970	0.452	0.805	0.960	0.008	0.797	0.860	0.004	0.017	0.066	0.005	0.005	0.005
Thraupidae	*Euphonia minuta*	0.517	0.343	0.121	0.394	0.623	0.743	0.251	0.402	0.544	0.206	0.343	0.402	0.069	0.100	0.166	0.013	0.020	0.051	0.023	0.031	0.037
Thraupidae	*Euphonia rufiventris*	0.157	0.236	0.192	0.027	0.855	0.991	0.010	0.064	0.098	0.010	0.014	0.619	0.011	0.019	0.047	0.009	0.026	0.073	0.010	0.012	0.028
Thraupidae	*Euphonia xanthogaster*	0.950	0.151	0.305	0.988	0.510	0.272	0.655	0.862	0.989	0.914	0.973	0.958	0.005	0.219	0.164	0.004	0.080	0.021	0.004	0.011	0.003
Thraupidae	*Iridophanes pulcherrimus*	0.010	0.006	0.006	0.067	0.380	0.000	0.984	0.232	0.396	0.997	0.672	0.874	0.919	0.998	0.999	0.326	0.820	0.967	0.001	0.001	0.001
Thraupidae	*Iridosornis analis*	0.014	0.011	0.011	0.983	0.323	0.050	0.864	0.973	0.793	0.886	0.988	0.991	0.178	0.513	0.872	0.010	0.023	0.132	0.024	0.022	0.023
Thraupidae	*Iridosornis jelskii*	0.025	0.018	0.018	0.822	0.739	0.551	0.449	0.718	0.743	0.364	0.464	0.171	0.003	0.056	0.052	0.195	0.144	0.195	0.003	0.005	0.005
Thraupidae	*Lamprospiza melanoleuca*	0.004	0.004	0.004	0.004	0.008	0.637	0.004	0.004	0.004	0.004	0.004	0.004	0.004	0.004	0.004	0.004	0.004	0.004	0.004	0.004	0.004
Thraupidae	*Pipraeidea melanonota*	0.441	0.003	0.003	0.510	0.073	0.629	0.310	0.625	0.325	0.480	0.681	0.784	0.273	0.790	0.991	0.007	0.677	0.929	0.003	0.036	0.003
Thraupidae	*Piranga leucoptera*	0.066	0.085	0.060	0.963	0.522	0.013	0.860	0.965	0.992	0.937	0.294	0.771	0.005	0.455	0.725	0.005	0.014	0.872	0.002	0.041	0.002
Thraupidae	*Ramphocelus carbo*	0.188	0.017	0.132	0.366	0.343	0.636	0.103	0.053	0.398	0.015	0.134	0.907	0.002	0.027	0.608	0.002	0.003	0.002	0.002	0.003	0.002
Thraupidae	*Ramphocelus nigrogularis*	0.027	0.010	0.002	0.070	0.861	0.728	0.002	0.015	0.292	0.002	0.014	0.004	0.002	0.005	0.003	0.002	0.002	0.002	0.002	0.003	0.003
Thraupidae	*Schistochlamys melanopis*	0.485	0.699	0.901	0.931	0.978	0.838	0.239	0.958	0.993	0.044	0.061	0.330	0.010	0.011	0.040	0.011	0.099	0.083	0.009	0.009	0.010
Thraupidae	*Tachyphonus cristatus*	0.413	0.340	0.053	0.005	0.329	0.853	0.004	0.195	0.542	0.005	0.009	0.473	0.004	0.005	0.017	0.004	0.004	0.005	0.003	0.003	0.003
Thraupidae	*Tachyphonus rufiventer*	0.676	0.052	0.028	0.982	0.993	0.993	0.054	0.974	0.983	0.024	0.847	0.646	0.003	0.003	0.024	0.003	0.005	0.005	0.003	0.004	0.003
Thraupidae	*Tangara arthus*	0.083	0.003	0.003	0.985	0.884	0.388	0.636	0.832	0.972	0.200	0.955	0.962	0.067	0.127	0.598	0.004	0.003	0.014	0.003	0.298	0.003
Thraupidae	*Tangara callophrys*	0.830	0.011	0.011	0.013	0.896	0.978	0.007	0.019	0.680	0.006	0.008	0.053	0.013	0.006	0.006	0.005	0.007	0.007	0.006	0.006	0.006
Thraupidae	*Tangara chilensis*	0.278	0.036	0.005	0.976	0.935	0.898	0.243	0.847	0.847	0.006	0.205	0.945	0.003	0.855	0.580	0.002	0.003	0.004	0.002	0.003	0.004
Thraupidae	*Tangara chrysotis*	0.246	0.046	0.046	0.986	0.984	0.232	0.779	0.441	0.993	0.154	0.928	0.295	0.464	0.899	0.844	0.003	0.025	0.025	0.003	0.006	0.006
Thraupidae	*Tangara cyanicollis*	0.548	0.006	0.248	0.988	0.731	0.762	0.749	0.782	0.953	0.724	0.993	0.977	0.401	0.966	0.991	0.007	0.034	0.021	0.003	0.009	0.003
Thraupidae	*Tangara cyanotis*	0.010	0.010	0.010	0.954	0.080	0.283	0.932	0.943	0.738	0.948	0.926	0.903	0.010	0.379	0.889	0.010	0.224	0.058	0.010	0.010	0.010
Thraupidae	*Tangara gyrola*	0.419	0.006	0.020	0.992	0.827	0.236	0.898	0.991	0.972	0.996	0.995	0.994	0.169	0.951	0.996	0.004	0.008	0.150	0.004	0.063	0.005
Thraupidae	*Tangara mexicana*	0.389	0.154	0.027	0.243	0.971	0.991	0.112	0.071	0.263	0.003	0.027	0.012	0.011	0.004	0.008	0.003	0.003	0.003	0.003	0.003	0.003
Thraupidae	*Tangara nigrocincta*	0.978	0.066	0.069	0.564	0.950	0.998	0.071	0.892	0.784	0.113	0.672	0.753	0.180	0.474	0.137	0.003	0.095	0.074	0.009	0.004	0.008
Thraupidae	*Tangara nigroviridis*	0.009	0.004	0.004	0.948	0.717	0.009	0.962	0.745	0.186	0.992	0.165	0.885	0.973	0.317	0.995	0.012	0.018	0.059	0.004	0.030	0.004
Thraupidae	*Tangara parzudakii*	0.003	0.003	0.003	0.977	0.556	0.007	0.995	0.625	0.139	0.991	0.732	0.318	0.045	0.254	0.996	0.075	0.034	0.432	0.010	0.003	0.003
Thraupidae	*Tangara punctata*	0.097	0.043	0.033	0.989	0.955	0.952	0.427	0.952	0.869	0.277	0.761	0.855	0.158	0.246	0.586	0.006	0.011	0.029	0.007	0.008	0.014
Thraupidae	*Tangara ruficervix*	0.005	0.005	0.005	0.978	0.084	0.013	0.987	0.636	0.421	0.991	0.136	0.197	0.133	0.579	0.990	0.022	0.451	0.660	0.005	0.005	0.005
Thraupidae	*Tangara schrankii*	0.203	0.025	0.003	0.231	0.742	0.969	0.013	0.980	0.962	0.002	0.064	0.679	0.002	0.002	0.038	0.002	0.002	0.002	0.002	0.002	0.002
Thraupidae	*Tangara vassorii*	0.010	0.009	0.008	0.309	0.167	0.012	0.159	0.337	0.182	0.545	0.119	0.263	0.550	0.940	0.993	0.972	0.021	0.123	0.301	0.325	0.006
Thraupidae	*Tangara velia*	0.926	0.487	0.126	0.010	0.365	0.993	0.006	0.008	0.094	0.003	0.038	0.006	0.007	0.003	0.003	0.003	0.003	0.003	0.004	0.003	0.003
Thraupidae	*Tangara viridicollis*	0.033	0.014	0.014	0.066	0.002	0.002	0.023	0.196	0.004	0.133	0.077	0.297	0.005	0.002	0.002	0.002	0.009	0.005	0.001	0.002	0.002
Thraupidae	*Tangara xanthocephala*	0.005	0.002	0.002	0.871	0.797	0.003	0.459	0.958	0.285	0.974	0.167	0.790	0.908	0.987	0.998	0.012	0.125	0.988	0.003	0.018	0.003
Thraupidae	*Tangara xanthogastra*	0.583	0.055	0.045	0.926	0.979	0.992	0.021	0.989	0.971	0.004	0.287	0.322	0.067	0.006	0.003	0.060	0.115	0.011	0.005	0.004	0.004
Thraupidae	*Tersina viridis*	0.590	0.065	0.285	0.994	0.427	0.570	0.245	0.679	0.887	0.957	0.971	0.866	0.500	0.258	0.199	0.007	0.048	0.447	0.005	0.005	0.005
Thraupidae	*Thlypopsis sordida*	0.741	0.123	0.057	0.021	0.914	0.306	0.016	0.184	0.350	0.013	0.126	0.350	0.004	0.034	0.239	0.002	0.884	0.639	0.002	0.002	0.002
Thraupidae	*Thraupis bonariensis*	0.008	0.002	0.002	0.002	0.002	0.002	0.006	0.089	0.011	0.183	0.020	0.135	0.002	0.068	0.068	0.003	0.066	0.299	0.002	0.002	0.002
Thraupidae	*Thraupis cyanocephala*	0.002	0.002	0.002	0.867	0.304	0.007	0.794	0.263	0.005	0.938	0.743	0.149	0.934	0.885	0.674	0.142	0.107	0.023	0.434	0.012	0.002
Thraupidae	*Thraupis episcopus*	0.109	0.267	0.617	0.267	0.549	0.190	0.458	0.299	0.762	0.931	0.430	0.370	0.019	0.671	0.414	0.024	0.007	0.011	0.006	0.008	0.006
Thraupidae	*Thraupis palmarum*	0.092	0.296	0.076	0.307	0.705	0.152	0.321	0.348	0.978	0.981	0.448	0.407	0.031	0.367	0.981	0.005	0.005	0.093	0.003	0.003	0.003
Thraupidae	*Trichothraupis melanops*	0.041	0.003	0.003	0.010	0.003	0.004	0.010	0.726	0.336	0.027	0.003	0.751	0.051	0.019	0.028	0.002	0.026	0.006	0.002	0.001	0.001
Tinamidae	*Crypturellus atrocapillus*	0.449	0.012	0.012	0.963	0.992	0.959	0.012	0.986	0.984	0.007	0.454	0.891	0.007	0.007	0.007	0.015	0.008	0.007	0.008	0.008	0.008
Tinamidae	*Crypturellus bartletti*	0.007	0.037	0.010	0.010	0.020	0.037	0.008	0.009	0.010	0.006	0.005	0.005	0.005	0.005	0.005	0.004	0.004	0.004	0.004	0.005	0.004

Tinamidae	*Crypturellus cinereus*	0.006	0.004	0.059	0.001	0.132	0.972	0.001	0.002	0.500	0.001	0.001	0.007	0.001	0.001	0.001	0.001	0.001	0.001	0.001	0.001	0.001
Tinamidae	*Crypturellus obsoletus*	0.021	0.002	0.002	0.861	0.483	0.790	0.006	0.946	0.983	0.010	0.689	0.733	0.326	0.012	0.010	0.021	0.899	0.774	0.005	0.003	0.003
Tinamidae	*Crypturellus soui*	0.273	0.451	0.866	0.893	0.992	0.974	0.518	0.779	0.950	0.346	0.925	0.818	0.017	0.447	0.065	0.021	0.051	0.057	0.003	0.003	0.003
Tinamidae	*Crypturellus strigulosus*	0.081	0.013	0.004	0.001	0.003	0.705	0.001	0.001	0.003	0.000	0.000	0.001	0.001	0.000	0.000	0.000	0.000	0.000	0.000	0.001	0.001
Tinamidae	*Crypturellus undulatus*	0.015	0.004	0.004	0.012	0.193	0.681	0.004	0.004	0.024	0.005	0.004	0.003	0.004	0.065	0.005	0.003	0.004	0.013	0.004	0.003	0.003
Tinamidae	*Crypturellus variegatus*	0.017	0.008	0.025	0.007	0.455	0.627	0.001	0.003	0.007	0.001	0.004	0.002	0.002	0.001	0.001	0.001	0.001	0.001	0.001	0.002	0.001
Tinamidae	*Nothocercus nigrocapillus*	0.003	0.003	0.003	0.004	0.003	0.003	0.778	0.003	0.003	0.706	0.006	0.003	0.043	0.256	0.187	0.049	0.484	0.524	0.017	0.016	0.016
Tinamidae	*Tinamus guttatus*	0.092	0.379	0.005	0.002	0.865	0.996	0.003	0.030	0.031	0.002	0.006	0.024	0.001	0.002	0.001	0.001	0.001	0.001	0.001	0.001	0.001
Tinamidae	*Tinamus major*	0.668	0.223	0.572	0.357	0.079	0.305	0.003	0.516	0.391	0.003	0.035	0.037	0.005	0.003	0.003	0.003	0.004	0.004	0.003	0.003	0.003
Tinamidae	*Tinamus tao*	0.905	0.014	0.002	0.875	0.967	0.983	0.125	0.974	0.987	0.023	0.834	0.948	0.002	0.002	0.002	0.002	0.002	0.002	0.002	0.002	0.002
Trogonidae	*Pharomachrus antisianus*	0.430	0.053	0.050	0.993	0.517	0.022	0.803	0.988	0.513	0.936	0.969	0.934	0.100	0.633	0.981	0.034	0.105	0.464	0.012	0.010	0.010
Trogonidae	*Pharomachrus auriceps*	0.003	0.002	0.002	0.756	0.160	0.002	0.931	0.963	0.147	0.989	0.941	0.647	0.418	0.723	0.992	0.006	0.033	0.077	0.004	0.014	0.002
Trogonidae	*Pharomachrus pavoninus*	0.918	0.837	0.395	0.132	0.837	0.998	0.012	0.130	0.225	0.021	0.010	0.016	0.043	0.017	0.017	0.010	0.122	0.007	0.083	0.173	0.012
Trogonidae	*Trogon collaris*	0.004	0.023	0.138	0.965	0.681	0.616	0.604	0.600	0.993	0.826	0.874	0.982	0.091	0.325	0.892	0.005	0.021	0.271	0.004	0.314	0.007
Trogonidae	*Trogon curucui*	0.058	0.024	0.003	0.181	0.593	0.983	0.004	0.198	0.379	0.019	0.044	0.122	0.003	0.003	0.008	0.003	0.006	0.006	0.005	0.003	0.003
Trogonidae	*Trogon melanurus*	0.007	0.157	0.052	0.004	0.820	0.984	0.018	0.004	0.004	0.003	0.004	0.004	0.002	0.002	0.003	0.002	0.005	0.004	0.002	0.002	0.015
Trogonidae	*Trogon personatus*	0.013	0.011	0.011	0.990	0.936	0.221	0.712	0.737	0.166	0.908	0.975	0.433	0.514	0.606	0.967	0.153	0.335	0.148	0.048	0.072	0.005
Trogonidae	*Trogon violaceus*	0.694	0.870	0.945	0.063	0.596	0.717	0.029	0.297	0.727	0.032	0.054	0.250	0.027	0.030	0.031	0.015	0.015	0.015	0.021	0.020	0.020
Trogonidae	*Trogon viridis*	0.243	0.024	0.106	0.007	0.165	0.957	0.061	0.117	0.961	0.045	0.028	0.256	0.006	0.006	0.043	0.002	0.005	0.009	0.002	0.004	0.005
Turdidae	*Catharus fuscater*	0.024	0.008	0.008	0.366	0.007	0.005	0.978	0.135	0.095	0.549	0.531	0.181	0.046	0.985	0.959	0.120	0.874	0.995	0.007	0.006	0.005
Turdidae	*Entomodestes leucotis*	0.071	0.012	0.012	0.488	0.215	0.039	0.473	0.473	0.645	0.444	0.478	0.463	0.009	0.066	0.105	0.005	0.022	0.013	0.011	0.006	0.002
Turdidae	*Myadestes ralloides*	0.030	0.002	0.002	0.990	0.272	0.004	0.940	0.978	0.922	0.961	0.771	0.928	0.788	0.996	0.972	0.229	0.319	0.563	0.001	0.002	0.002
Turdidae	*Turdus albicollis*	0.068	0.024	0.016	0.520	0.552	0.921	0.115	0.858	0.948	0.529	0.258	0.049	0.520	0.393	0.734	0.002	0.007	0.005	0.037	0.094	0.094
Turdidae	*Turdus chiguanco*	0.001	0.001	0.001	0.003	0.004	0.001	0.001	0.003	0.002	0.001	0.001	0.004	0.003	0.001	0.002	0.001	0.017	0.008	0.001	0.001	0.001
Turdidae	*Turdus fuscater*	0.004	0.004	0.004	0.122	0.398	0.051	0.543	0.188	0.185	0.961	0.074	0.065	0.917	0.366	0.991	0.014	0.013	0.522	0.629	0.753	0.005
Turdidae	*Turdus hauxwelli*	0.037	0.041	0.038	0.125	0.932	0.603	0.027	0.091	0.069	0.096	0.046	0.057	0.011	0.163	0.249	0.004	0.004	0.004	0.011	0.004	0.004
Turdidae	*Turdus ignobilis*	0.332	0.025	0.562	0.985	0.654	0.475	0.052	0.943	0.362	0.369	0.923	0.920	0.294	0.906	0.766	0.024	0.027	0.031	0.004	0.031	0.004
Turdidae	*Turdus leucops*	0.405	0.031	0.037	0.919	0.450	0.360	0.753	0.881	0.640	0.902	0.592	0.632	0.247	0.995	0.994	0.041	0.955	0.979	0.022	0.009	0.010
Turdidae	*Turdus nigriceps*	0.592	0.002	0.002	0.873	0.711	0.700	0.116	0.678	0.969	0.580	0.819	0.652	0.739	0.552	0.667	0.926	0.962	0.892	0.001	0.001	0.002
Turdidae	*Turdus serranus*	0.006	0.005	0.005	0.944	0.394	0.070	0.173	0.752	0.242	0.986	0.687	0.303	0.739	0.975	0.955	0.065	0.698	0.638	0.022	0.098	0.010
Tyrannidae	*Elaenia albiceps*	0.042	0.003	0.003	0.005	0.003	0.041	0.004	0.082	0.007	0.722	0.005	0.078	0.322	0.144	0.292	0.128	0.020	0.285	0.783	0.303	0.877
Tyrannidae	*Elaenia flavogaster*	0.010	0.096	0.483	0.722	0.975	0.234	0.084	0.960	0.988	0.957	0.917	0.805	0.104	0.865	0.178	0.003	0.019	0.836	0.003	0.004	0.003
Tyrannidae	*Elaenia gigas*	0.992	0.314	0.300	0.991	0.995	0.993	0.364	0.980	0.989	0.017	0.580	0.936	0.027	0.049	0.019	0.015	0.047	0.054	0.033	0.021	0.018
Tyrannidae	*Elaenia obscura*	0.017	0.017	0.017	0.186	0.023	0.017	0.233	0.093	0.039	0.298	0.194	0.067	0.254	0.347	0.351	0.567	0.916	0.934	0.044	0.069	0.053
Tyrannidae	*Elaenia pallatangae*	0.004	0.013	0.041	0.161	0.979	0.005	0.951	0.421	0.358	0.994	0.248	0.534	0.322	0.913	0.595	0.015	0.880	0.816	0.043	0.005	0.004
Tyrannidae	*Lophotriccus pileatus*	0.032	0.006	0.006	0.948	0.956	0.289	0.988	0.989	0.978	0.855	0.986	0.934	0.040	0.720	0.315	0.008	0.008	0.625	0.004	0.005	0.004
Tyrannidae	*Mionectes macconnelli*	0.003	0.013	0.013	0.499	0.984	0.992	0.014	0.478	0.938	0.003	0.251	0.228	0.003	0.002	0.003	0.002	0.002	0.002	0.002	0.003	0.003
Tyrannidae	*Mionectes oleagineus*	0.335	0.192	0.770	0.958	0.995	0.994	0.276	0.989	0.970	0.964	0.567	0.598	0.002	0.886	0.822	0.002	0.005	0.003	0.002	0.026	0.002
Tyrannidae	*Mionectes olivaceus*	0.547	0.043	0.041	0.996	0.507	0.859	0.743	0.997	0.997	0.929	0.816	0.893	0.246	0.164	0.197	0.004	0.893	0.599	0.005	0.004	0.003
Tyrannidae	*Mionectes striaticollis*	0.537	0.004	0.004	0.325	0.724	0.520	0.263	0.847	0.337	0.994	0.562	0.919	0.894	0.175	0.993	0.139	0.310	0.325	0.007	0.093	0.014
Tyrannidae	*Zimmerius bolivianus*	0.006	0.003	0.003	0.956	0.209	0.005	0.737	0.926	0.972	0.385	0.912	0.733	0.009	0.082	0.092	0.005	0.236	0.524	0.004	0.001	0.001
Tyrannidae	*Zimmerius gracilipes*	0.036	0.030	0.087	0.270	0.448	0.969	0.014	0.042	0.222	0.010	0.339	0.059	0.011	0.010	0.010	0.011	0.011	0.017	0.010	0.013	0.013

Table S3. Current and future community composition at all seven elevational levels, including probabilities of occurrence derived from species distribution models (SDMs) of all 232 frugivorous bird species of the Manú Biosphere Reserve, Peru. Based on the MIROC5 climate model, representative concentration pathway (RCP) 6.0. Species' elevational ranges were projected according to four potential dispersal scenarios: no range change, range contraction (lower range limit moves upwards), range expansion (upper range limit moves upwards), and range shift (both lower and upper range limit move upwards). SDMs were based on current and future local temperature and precipitation values.

		500 m					1000 m					1500 m					2000 m				
Family	Bird species	current	no range change	contraction	expansion	shift	current	no range change	contraction	expansion	shift	current	no range change	contraction	expansion	shift	current	no range change	contraction	expansion	shift
Cardinalidae	*Cyanocompsa cyanoides*	0.354	0.936	0	0.936	0	0.965	0.975	0.975	0.975	0.975	0	0.968	0	0.968	0.968	0	0.758	0	0	0
Cardinalidae	*Pheucticus aureoventris*	0	0.008	0	0	0	0.302	0.027	0.027	0.027	0.027	0.008	0.183	0.183	0.183	0.183	0.028	0.321	0.321	0.321	0.321
Cardinalidae	*Pheucticus chrysogaster*	0	0.003	0	0	0	0.063	0.011	0	0.011	0	0.476	0.179	0.179	0.179	0.179	0.520	0.054	0.054	0.054	0.054
Cardinalidae	*Saltator aurantiirostris*	0	0.002	0	0	0	0	0.002	0	0	0	0	0.022	0	0	0	0	0.007	0	0	0
Cardinalidae	*Saltator coerulescens*	0.023	0.013	0	0.013	0	0	0.848	0	0.848	0.848	0	0.683	0	0	0	0	0.582	0	0	0
Cardinalidae	*Saltator grossus*	0.195	0.553	0	0.553	0	0.988	0.841	0.841	0.841	0.841	0	0.505	0	0	0	0	0.830	0	0	0
Cardinalidae	*Saltator maximus*	0.514	0.452	0	0.452	0	0.903	0.424	0.424	0.424	0.424	0.136	0.958	0.958	0.958	0.958	0.941	0.634	0.634	0.634	0.634
Columbidae	*Geotrygon montana*	0.731	0.286	0	0.286	0	0.270	0.567	0.567	0.567	0.567	0	0.911	0	0.911	0.911	0	0.517	0	0	0
Columbidae	*Patagioenas cayennensis*	0.067	0.013	0	0.013	0	0	0.466	0	0.466	0.466	0	0.956	0	0	0	0	0.045	0	0	0
Columbidae	*Patagioenas fasciata*	0	0.010	0	0	0	0	0.432	0	0	0	0.790	0.496	0	0.496	0	0.993	0.899	0.899	0.899	0.899
Columbidae	*Patagioenas plumbea*	0.075	0.188	0	0.188	0	0.344	0.272	0.272	0.272	0.272	0.030	0.978	0.978	0.978	0.978	0	0.923	0	0.923	0.923
Columbidae	*Patagioenas subvinacea*	0.008	0.020	0	0.020	0	0.593	0.955	0.955	0.955	0.955	0.698	0.792	0.792	0.792	0.792	0	0.719	0	0	0
Corvidae	*Cyanocorax cyanomelas*	0.338	0.004	0	0.004	0	0.496	0.701	0.701	0.701	0.701	0	0.254	0	0	0	0	0.044	0	0	0
Corvidae	*Cyanocorax violaceus*	0.544	0.005	0	0.005	0	0.207	0.971	0.971	0.971	0.971	0	0.484	0	0.484	0.484	0	0.089	0	0	0
Corvidae	*Cyanocorax yncas*	0	0.009	0	0	0	0	0.660	0	0	0	0.453	0.402	0	0.402	0	0.994	0.992	0.992	0.992	0.992
Cotingidae	*Ampelioides tschudii*	0	0.080	0	0	0	0.765	0.977	0	0.977	0	0	0.951	0	0.951	0.951	0	0.918	0	0	0
Cotingidae	*Ampelion rubrocristatus*	0	0.005	0	0	0	0	0.004	0	0	0	0	0.014	0	0	0	0	0.090	0	0	0
Cotingidae	*Ampelion rufaxilla*	0	0.024	0	0	0	0	0.221	0	0	0	0	0.941	0	0	0	0.953	0.890	0.890	0.890	0.890
Cotingidae	*Cephalopterus ornatus*	0.371	0.175	0	0.175	0	0.044	0.842	0.842	0.842	0.842	0.534	0.792	0.792	0.792	0.792	0	0.047	0	0.047	0.047
Cotingidae	*Conioptilon mcilhennyi*	0	0.014	0	0.014	0	0	0.211	0	0	0	0	0.063	0	0	0	0	0.070	0	0	0
Cotingidae	*Cotinga cayana*	0.070	0.064	0	0.064	0	0	0.667	0	0.667	0.667	0	0.163	0	0	0	0	0.064	0	0	0
Cotingidae	*Cotinga maynana*	0.023	0.003	0	0.003	0	0.271	0.962	0.962	0.962	0.962	0	0.057	0	0	0	0	0.003	0	0	0
Cotingidae	*Gymnoderus foetidus*	0.010	0.010	0	0.010	0	0	0.006	0	0	0	0	0.002	0	0	0	0	0.003	0	0	0
Cotingidae	*Iodopleura isabellae*	0.842	0.116	0	0.116	0	0	0.920	0	0.920	0.920	0	0.014	0	0	0	0	0.003	0	0	0
Cotingidae	*Laniocera hypopyrra*	0.016	0.015	0	0.015	0	0	0.691	0	0.691	0.691	0	0.019	0	0	0	0	0.015	0	0	0
Cotingidae	*Lipaugus vociferans*	0.041	0.009	0	0.009	0	0	0.045	0	0.045	0.045	0	0.002	0	0	0	0	0.002	0	0	0
Cotingidae	*Oxyruncus cristatus*	0	0.155	0	0	0	0.729	0.618	0	0.618	0	0	0.547	0	0.547	0.547	0	0.809	0	0	0
Cotingidae	*Pipreola arcuata*	0	0.001	0	0	0	0	0.029	0	0	0	0	0.114	0	0	0	0.906	0.043	0	0.043	0
Cotingidae	*Pipreola frontalis*	0	0.036	0	0	0	0.926	0.865	0	0.865	0	0.913	0.921	0.921	0.921	0.921	0	0.922	0	0.922	0.922
Cotingidae	*Pipreola intermedia*	0	0.003	0	0	0	0	0.009	0	0	0	0.036	0.010	0	0.010	0	0.054	0.030	0.030	0.030	0.030
Cotingidae	*Porphyrolaema porphyrolaema*	0	0.049	0	0.049	0	0	0.491	0	0	0	0	0.120	0	0	0	0	0.047	0	0	0
Cotingidae	*Querula purpurata*	0.021	0.030	0	0.030	0	0.436	0.932	0.932	0.932	0.932	0	0.950	0	0	0	0	0.573	0	0	0
Cotingidae	*Rupicola peruvianus*	0	0.003	0	0	0	0.996	0.766	0	0.766	0	0.995	0.998	0.998	0.998	0.998	0.909	0.988	0.988	0.988	0.988
Cotingidae	*Snowornis subalaris*	0	0.010	0	0	0	0.976	0.956	0	0.956	0	0	0.969	0	0.969	0.969	0	0.920	0	0	0
Cotingidae	*Tityra cayana*	0	0.027	0	0.027	0	0	0.063	0	0	0	0	0.036	0	0	0	0	0.004	0	0	0
Cotingidae	*Tityra inquisitor*	0.856	0.061	0	0.061	0	0	0.669	0	0.669	0.669	0	0.951	0	0	0	0	0.508	0	0	0

Cotingidae	*Tityra semifasciata*	0.424	0.600	0	0.600	0	0.967	0.968	0.968	0.968	0.968	0.984	0.913	0.913	0.913	0.913	0	0.669	0	0	0
Cracidae	*Aburria aburri*	0	0.017	0	0	0	0.985	0.988	0	0.988	0	0.995	0.999	0.999	0.999	0.999	0	0.988	0	0.988	0.988
Cracidae	*Mitu tuberosum*	0.903	0.034	0	0.034	0	0.044	0.972	0.972	0.972	0.972	0	0.028	0	0	0	0	0.002	0	0	0
Cracidae	*Ortalis guttata*	0.577	0.006	0	0.006	0	0.978	0.769	0.769	0.769	0.769	0.136	0.968	0.968	0.968	0.968	0	0.743	0	0	0
Cracidae	*Penelope jacquacu*	0.078	0.006	0	0.006	0	0.139	0.130	0.130	0.130	0.130	0.004	0.337	0.337	0.337	0.337	0	0.048	0	0	0
Cracidae	*Penelope montagnii*	0	0.007	0	0	0	0.224	0.014	0	0.014	0	0.109	0.123	0.123	0.123	0.123	0.491	0.686	0.686	0.686	0.686
Cracidae	*Pipile cumanensis*	0.113	0.012	0	0.012	0	0	0.948	0	0	0	0	0.006	0	0	0	0	0.005	0	0	0
Icteridae	*Cacicus cela*	0.789	0.023	0	0.023	0	0.008	0.700	0.700	0.700	0.700	0	0.014	0	0	0	0	0.013	0	0	0
Icteridae	*Cacicus chrysonotus*	0	0.002	0	0	0	0	0.077	0	0	0	0	0.700	0	0	0	0	0.119	0	0	0
Icteridae	*Cacicus haemorrhous*	0.006	0.206	0	0.206	0	0.007	0.022	0.022	0.022	0.022	0	0.028	0	0	0	0	0.003	0	0	0
Icteridae	*Clypicterus oseryi*	0.988	0.058	0	0.058	0	0	0.195	0	0.195	0.195	0	0.101	0	0	0	0	0.036	0	0	0
Icteridae	*Icterus icterus*	0.074	0.028	0	0.028	0	0	0.040	0	0.040	0.040	0	0.042	0	0	0	0	0.066	0	0	0
Icteridae	*Molothrus oryzivorus*	0.216	0.115	0	0.115	0	0	0.896	0	0.896	0.896	0	0.400	0	0	0	0	0.885	0	0	0
Icteridae	*Psarocolius angustifrons*	0.244	0.009	0	0.009	0	0.493	0.813	0.813	0.813	0.813	0.073	0.784	0.784	0.784	0.784	0.707	0.954	0.954	0.954	0.954
Icteridae	*Psarocolius atrovirens*	0	0.002	0	0	0	0.699	0.707	0	0.707	0	0.775	0.981	0.981	0.981	0.981	0.551	0.922	0.922	0.922	0.922
Icteridae	*Psarocolius bifasciatus*	0.932	0.066	0	0.066	0	0	0.346	0	0.346	0.346	0	0.807	0	0	0	0	0.016	0	0	0
Icteridae	*Psarocolius decumanus*	0.892	0.499	0	0.499	0	0.978	0.653	0.653	0.653	0.653	0	0.943	0	0.943	0.943	0	0.914	0	0	0
Odontophoridae	*Odontophorus balliviani*	0	0.040	0	0	0	0.148	0.030	0	0.030	0	0.956	0.065	0.065	0.065	0.065	0.955	0.420	0.420	0.420	0.420
Odontophoridae	*Odontophorus speciosus*	0	0.007	0	0	0	0.981	0.064	0	0.064	0	0.828	0.986	0.986	0.986	0.986	0.418	0.968	0.968	0.968	0.968
Odontophoridae	*Odontophorus stellatus*	0.016	0.008	0	0.008	0	0.253	0.431	0.431	0.431	0.431	0	0.253	0	0	0	0	0.170	0	0	0
Picidae	*Celeus elegans*	0.109	0.031	0	0.031	0	0	0.008	0	0	0	0	0.003	0	0	0	0	0.003	0	0	0
Picidae	*Celeus flavus*	0.201	0.173	0	0.173	0	0	0.107	0	0	0	0	0.010	0	0	0	0	0.008	0	0	0
Picidae	*Celeus grammicus*	0.791	0.621	0	0.621	0	0.030	0.138	0.138	0.138	0.138	0	0.033	0	0.033	0.033	0	0.016	0	0	0
Picidae	*Celeus spectabilis*	0.201	0.000	0	0	0	0	0.570	0	0	0	0	0.303	0	0	0	0	0.187	0	0	0
Picidae	*Celeus torquatus*	0.190	0.152	0	0.152	0	0	0.016	0	0	0	0	0.010	0	0	0	0	0.011	0	0	0
Picidae	*Colaptes rubiginosus*	0	0.005	0	0	0	0.985	0.804	0	0.804	0	0.995	0.978	0.978	0.978	0.978	0.986	0.821	0.821	0.821	0.821
Pipridae	*Chiroxiphia boliviana*	0	0.007	0	0	0	0.142	0.121	0	0.121	0	0.123	0.883	0.883	0.883	0.883	0.028	0.198	0.198	0.198	0.198
Pipridae	*Chiroxiphia pareola*	0.463	0.236	0	0.236	0	0	0.771	0	0.771	0.771	0	0.085	0	0	0	0	0.043	0	0	0
Pipridae	*Lepidothrix coeruleocapilla*	0	0.007	0	0	0	0.972	0.448	0	0.448	0	0.044	0.993	0.993	0.993	0.993	0	0.573	0	0.573	0.573
Pipridae	*Lepidothrix coronata*	0.825	0.579	0	0.579	0	0	0.774	0	0.774	0.774	0	0.465	0	0	0	0	0.035	0	0	0
Pipridae	*Machaeropterus pyrocephalus*	0.074	0.005	0	0.005	0	0.240	0.984	0.984	0.984	0.984	0	0.064	0	0.064	0.064	0	0.023	0	0	0
Pipridae	*Manacus manacus*	0.895	0.495	0	0.495	0	0	0.908	0	0.908	0.908	0	0.930	0	0	0	0	0.835	0	0	0
Pipridae	*Neopelma sulphureiventer*	0.311	0.137	0	0.137	0	0	0.077	0	0	0	0	0.035	0	0	0	0	0.037	0	0	0
Pipridae	*Pipra chloromeros*	0.409	0.013	0	0.013	0	0.851	0.925	0.925	0.925	0.925	0	0.783	0	0.783	0.783	0	0.432	0	0	0
Pipridae	*Pipra fasciicauda*	0.005	0.003	0	0.003	0	0.413	0.804	0.804	0.804	0.804	0	0.641	0	0	0	0	0.006	0	0	0
Pipridae	*Xenopipo holochlora*	0.983	0.262	0	0.262	0	0.799	0.990	0.990	0.990	0.990	0	0.960	0	0	0	0	0.812	0	0	0
Pipridae	*Xenopipo unicolor*	0	0.002	0	0	0	0.500	0.133	0	0.133	0	0.916	0.837	0.837	0.837	0.837	0	0.475	0	0.475	0.475
Psittacidae	*Amazona farinosa*	0.033	0.071	0	0.071	0	0.034	0.302	0.302	0.302	0.302	0	0.344	0	0.344	0.344	0	0.009	0	0	0
Psittacidae	*Amazona mercenaria*	0	0.028	0	0	0	0.974	0.659	0	0.659	0	0.974	0.970	0.970	0.970	0.970	0.977	0.976	0.976	0.976	0.976
Psittacidae	*Amazona ochrocephala*	0.009	0.018	0	0.018	0	0	0.194	0	0.194	0.194	0	0.018	0	0	0	0	0.012	0	0	0
Psittacidae	*Ara ararauna*	0.506	0.034	0	0.034	0	0.010	0.098	0.098	0.098	0.098	0	0.070	0	0	0	0	0.022	0	0	0
Psittacidae	*Ara chloropterus*	0.079	0.033	0	0.033	0	0.004	0.063	0.063	0.063	0.063	0	0.007	0	0.007	0.007	0	0.019	0	0	0
Psittacidae	*Ara macao*	0.007	0.020	0	0.020	0	0.070	0.035	0.035	0.035	0.035	0	0.077	0	0	0	0	0.042	0	0	0
Psittacidae	*Ara militaris*	0	0.045	0	0	0	0.994	0.971	0.971	0.971	0.971	0.747	0.994	0.994	0.994	0.994	0	0.587	0	0.587	0.587
Psittacidae	*Ara severus*	0.114	0.712	0	0.712	0	0.120	0.102	0.102	0.102	0.102	0	0.862	0	0.862	0.862	0	0.015	0	0	0
Psittacidae	*Aratinga leucophthalma*	0.019	0.011	0	0.011	0	0.021	0.181	0.181	0.181	0.181	0.006	0.149	0.149	0.149	0.149	0	0.007	0	0.007	0.007
Psittacidae	*Aratinga mitrata*	0	0.001	0	0	0	0	0.001	0	0	0	0	0.004	0	0	0	0.001	0.008	0.008	0.008	0.008
Psittacidae	*Aratinga weddellii*	0.179	0.015	0	0.015	0	0	0.569	0	0.569	0.569	0	0.105	0	0	0	0	0.004	0	0	0
Psittacidae	*Bolborhynchus lineola*	0	0.042	0	0	0	0	0.118	0	0	0	0.766	0.609	0.609	0.609	0.609	0.800	0.632	0.632	0.632	0.632
Psittacidae	*Bolborhynchus orbygnesius*	0	0.011	0	0	0	0	0.014	0	0	0	0	0.050	0	0	0	0	0.067	0	0	0

Psittacidae	*Brotogeris cyanoptera*	0.052	0.007	0	0.007	0	0	0.154	0	0	0	0	0.010	0	0	0	0	0.002	0	0	0
Psittacidae	*Brotogeris sanctithomae*	0.041	0.184	0	0.184	0	0	0.059	0	0	0	0	0.059	0	0	0	0	0.031	0	0	0
Psittacidae	*Forpus modestus*	0.017	0.017	0	0.017	0	0	0.348	0	0	0	0	0.360	0	0	0	0	0.129	0	0	0
Psittacidae	*Nannopsittaca dachilleae*	0.263	0.992	0	0.992	0	0.009	0.010	0.010	0.010	0.010	0	0.016	0	0	0	0	0.002	0	0	0
Psittacidae	*Orthopsittaca manilata*	0.098	0.007	0	0.007	0	0	0.221	0	0.221	0.221	0	0.019	0	0	0	0	0.005	0	0	0
Psittacidae	*Pionites leucogaster*	0.004	0.000	0	0	0	0	0.000	0	0	0	0	0.000	0	0	0	0	0.000	0	0	0
Psittacidae	*Pionus menstruus*	0.137	0.161	0	0.161	0	0.489	0.518	0.518	0.518	0.518	0.078	0.546	0.546	0.546	0.546	0.653	0.039	0.039	0.039	0.039
Psittacidae	*Pionus tumultuosus*	0	0.041	0	0	0	0	0.048	0	0	0	0.959	0.029	0.029	0.029	0.029	0.989	0.076	0.076	0.076	0.076
Psittacidae	*Primolius couloni*	0.109	0.006	0	0.006	0	0.050	0.975	0.975	0.975	0.975	0	0.020	0	0.020	0.020	0	0.029	0	0	0
Psittacidae	*Pyrilia barrabandi*	0.006	0.006	0	0.006	0	0	0.007	0	0	0	0	0.006	0	0	0	0	0.006	0	0	0
Psittacidae	*Pyrrhura roseifrons*	0.005	0.005	0	0.005	0	0.005	0.646	0.646	0.646	0.646	0	0.005	0	0	0	0	0.005	0	0	0
Psittacidae	*Pyrrhura rupicola*	0.018	0.018	0	0.018	0	0.433	0.508	0.508	0.508	0.508	0	0.226	0	0	0	0	0.213	0	0	0
Psittacidae	*Touit huetii*	0.264	0.318	0	0.318	0	0.005	0.018	0.018	0.018	0.018	0	0.007	0	0.007	0.007	0	0.005	0	0	0
Psophiidae	*Psophia leucoptera*	0.995	0.274	0	0.274	0	0.057	0.563	0.563	0.563	0.563	0	0.061	0	0	0	0	0.028	0	0	0
Ramphastidae	*Andigena hypoglauca*	0	0.003	0	0	0	0	0.003	0	0	0	0.017	0.003	0	0.003	0	0.008	0.003	0.003	0.003	0.003
Ramphastidae	*Aulacorhynchus coeruleicinctis*	0	0.009	0	0	0	0	0.010	0	0	0	0.915	0.871	0.871	0.871	0.871	0.707	0.928	0.928	0.928	0.928
Ramphastidae	*Aulacorhynchus derbianus*	0	0.359	0	0	0	0.984	0.984	0	0.984	0	0.705	0.981	0.981	0.981	0.981	0	0.949	0	0.949	0.949
Ramphastidae	*Aulacorhynchus prasinus*	0.116	0.001	0	0.001	0	0.937	0.976	0.976	0.976	0.976	0.948	0.841	0.841	0.841	0.841	0	0.766	0	0	0
Ramphastidae	*Capito auratus*	0.079	0.008	0	0.008	0	0.018	0.143	0.143	0.143	0.143	0	0.014	0	0.014	0.014	0	0.010	0	0	0
Ramphastidae	*Eubucco richardsoni*	0.961	0.012	0	0.012	0	0.376	0.949	0.949	0.949	0.949	0	0.582	0	0	0	0	0.039	0	0	0
Ramphastidae	*Eubucco tucinkae*	0.602	0.073	0	0.073	0	0	0.997	0	0.997	0.997	0	0.112	0	0	0	0	0.086	0	0	0
Ramphastidae	*Eubucco versicolor*	0	0.246	0	0	0	0.909	0.167	0	0.167	0	0.063	0.849	0.849	0.849	0.849	0.004	0.753	0.753	0.753	0.753
Ramphastidae	*Pteroglossus azara*	0.887	0.075	0	0.075	0	0.034	0.976	0.976	0.976	0.976	0	0.013	0	0.013	0.013	0	0.012	0	0	0
Ramphastidae	*Pteroglossus beauharnaesii*	0.096	0.015	0	0.015	0	0	0.236	0	0.236	0.236	0	0.005	0	0	0	0	0.004	0	0	0
Ramphastidae	*Pteroglossus castanotis*	0.645	0.017	0	0.017	0	0.135	0.414	0.414	0.414	0.414	0	0.093	0	0	0	0	0.050	0	0	0
Ramphastidae	*Pteroglossus inscriptus*	0.384	0.037	0	0.037	0	0	0.829	0	0	0	0	0.027	0	0	0	0	0.021	0	0	0
Ramphastidae	*Ramphastos tucanus*	0.007	0.015	0	0.015	0	0	0.012	0	0.012	0.012	0	0.040	0	0	0	0	0.179	0	0	0
Ramphastidae	*Ramphastos vitellinus*	0.204	0.204	0	0.204	0	0	0.283	0	0	0	0	0.009	0	0	0	0	0.122	0	0	0
Ramphastidae	*Selenidera reinwardtii*	0.909	0.084	0	0.084	0	0.154	0.686	0.686	0.686	0.686	0.001	0.064	0.064	0.064	0.064	0	0.007	0	0.007	0.007
Steatornithidae	*Steatornis caripensis*	0.992	0.056	0	0.056	0	0	0.966	0	0.966	0.966	0	0.818	0	0	0	0	0.983	0	0	0
Thraupidae	*Anisognathus igniventris*	0	0.003	0	0	0	0	0.203	0	0	0	0	0.185	0	0	0	0	0.018	0	0	0
Thraupidae	*Anisognathus somptuosus*	0	0.004	0	0	0	0	0.052	0	0	0	0.684	0.871	0	0.871	0	0.998	0.414	0.414	0.414	0.414
Thraupidae	*Buthraupis montana*	0	0.006	0	0	0	0	0.232	0	0	0	0	0.154	0	0	0	0	0.006	0	0	0
Thraupidae	*Catamblyrhynchus diadema*	0	0.010	0	0	0	0	0.031	0	0	0	0	0.129	0	0	0	0.862	0.119	0	0.119	0
Thraupidae	*Chlorochrysa calliparaea*	0	0.007	0	0	0	0.876	0.745	0	0.745	0	0.585	0.925	0.925	0.925	0.925	0.988	0.119	0.119	0.119	0.119
Thraupidae	*Chlorophanes spiza*	0.924	0.501	0	0.501	0	0.960	0.930	0.930	0.930	0.930	0	0.514	0	0.514	0.514	0	0.935	0	0	0
Thraupidae	*Chlorophonia cyanea*	0.051	0.004	0	0.004	0	0.913	0.339	0.339	0.339	0.339	0.993	0.904	0.904	0.904	0.904	0.335	0.900	0.900	0.900	0.900
Thraupidae	*Chlorornis riefferii*	0	0.003	0	0	0	0	0.323	0	0	0	0	0.798	0	0	0	0	0.658	0	0	0
Thraupidae	*Chlorospingus canigularis*	0	0.006	0	0	0	0.970	0.444	0	0.444	0	0.881	0.812	0.812	0.812	0.812	0	0.916	0	0	0
Thraupidae	*Chlorospingus flavigularis*	0	0.010	0	0	0	0.985	0.662	0	0.662	0	0.995	0.992	0.992	0.992	0.992	0.985	0.994	0.994	0.994	0.994
Thraupidae	*Chlorospingus ophthalmicus*	0	0.002	0	0	0	0.974	0.358	0	0.358	0	0.129	0.289	0.289	0.289	0.289	0.166	0.209	0.209	0.209	0.209
Thraupidae	*Chlorospingus parvirostris*	0	0.007	0	0	0	0	0.148	0	0	0	0.966	0.929	0.929	0.929	0.929	0.951	0.710	0.710	0.710	0.710
Thraupidae	*Cissopis leverianus*	0.982	0.020	0	0.020	0	0.971	0.965	0.965	0.965	0.965	0.010	0.856	0.856	0.856	0.856	0	0.030	0	0	0
Thraupidae	*Conirostrum cinereum*	0	0.003	0	0	0	0	0.056	0	0	0	0	0.078	0	0	0	0	0.013	0	0	0
Thraupidae	*Conirostrum speciosum*	0.016	0.010	0	0.010	0	0	0.105	0	0.105	0.105	0	0.164	0	0	0	0	0.136	0	0	0
Thraupidae	*Cyanerpes caeruleus*	0.868	0.111	0	0.111	0	0.665	0.579	0.579	0.579	0.579	0	0.835	0	0.835	0.835	0	0.865	0	0	0
Thraupidae	*Cyanerpes cyaneus*	0.018	0.107	0	0.107	0	0.005	0.083	0.083	0.083	0.083	0	0.007	0	0	0	0	0.013	0	0	0
Thraupidae	*Dacnis cayana*	0.952	0.736	0	0.736	0	0.840	0.838	0.838	0.838	0.838	0.093	0.216	0.216	0.216	0.216	0	0.492	0	0	0
Thraupidae	*Dacnis flaviventer*	0.894	0.263	0	0.263	0	0.209	0.950	0.950	0.950	0.950	0	0.099	0	0	0	0	0.020	0	0	0
Thraupidae	*Dacnis lineata*	0.344	0.088	0	0.088	0	0.893	0.990	0.990	0.990	0.990	0	0.543	0	0.543	0.543	0	0.352	0	0	0

Thraupidae	*Delothraupis castaneoventris*	0	0.004	0	0	0	0	0.032	0	0	0	0	0.240	0	0	0	0	0.130	0	0	0
Thraupidae	*Diglossa brunneiventris*	0	0.001	0	0	0	0	0.001	0	0	0	0	0.001	0	0	0	0	0.001	0	0	0
Thraupidae	*Diglossa caerulescens*	0	0.004	0	0	0	0	0.006	0	0	0	0.041	0.992	0	0.992	0	0.021	0.860	0.860	0.860	0.860
Thraupidae	*Diglossa cyanea*	0	0.002	0	0	0	0	0.090	0	0	0	0.075	0.015	0	0.015	0	0.982	0.147	0.147	0.147	0.147
Thraupidae	*Diglossa glauca*	0	0.049	0	0	0	0.986	0.882	0	0.882	0	0.983	0.986	0.986	0.986	0.986	0.950	0.985	0.985	0.985	0.985
Thraupidae	*Diglossa mystacalis*	0	0.001	0	0	0	0	0.324	0	0	0	0	0.019	0	0	0	0	0.008	0	0	0
Thraupidae	*Diglossa sittoides*	0	0.002	0	0	0	0	0.018	0	0	0	0	0.659	0	0	0	0.967	0.921	0	0.921	0
Thraupidae	*Dubusia taeniata*	0	0.001	0	0	0	0	0.001	0	0	0	0	0.009	0	0	0	0.106	0.008	0	0.008	0
Thraupidae	*Euphonia chlorotica*	0	0.019	0	0.019	0	0	0.070	0	0	0	0	0.011	0	0	0	0	0.024	0	0	0
Thraupidae	*Euphonia chrysopasta*	0.882	0.062	0	0.062	0	0	0.918	0	0.918	0.918	0	0.459	0	0	0	0	0.318	0	0	0
Thraupidae	*Euphonia laniirostris*	0.020	0.152	0	0.152	0	0.794	0.922	0.922	0.922	0.922	0.909	0.840	0.840	0.840	0.840	0	0.901	0	0	0
Thraupidae	*Euphonia mesochrysa*	0	0.075	0	0	0	0.968	0.048	0.048	0.048	0.048	0.935	0.981	0.981	0.981	0.981	0	0.805	0	0.805	0.805
Thraupidae	*Euphonia minuta*	0.517	0.343	0	0.343	0	0.394	0.623	0.623	0.623	0.623	0	0.402	0	0	0	0	0.343	0	0	0
Thraupidae	*Euphonia rufiventris*	0.157	0.236	0	0.236	0	0	0.855	0	0.855	0.855	0	0.064	0	0	0	0	0.014	0	0	0
Thraupidae	*Euphonia xanthogaster*	0.950	0.151	0	0.151	0	0.988	0.510	0.510	0.510	0.510	0.655	0.862	0.862	0.862	0.862	0.914	0.973	0.973	0.973	0.973
Thraupidae	*Iridophanes pulcherrimus*	0	0.006	0	0	0	0	0.380	0	0	0	0.984	0.232	0.232	0.232	0.232	0	0.672	0	0.672	0.672
Thraupidae	*Iridosornis analis*	0	0.011	0	0	0	0.983	0.323	0	0.323	0	0.864	0.973	0.973	0.973	0.973	0.886	0.988	0.988	0.988	0.988
Thraupidae	*Iridosornis jelskii*	0	0.018	0	0	0	0	0.739	0	0	0	0	0.718	0	0	0	0	0.464	0	0	0
Thraupidae	*Lamprospiza melanoleuca*	0.004	0.004	0	0.004	0	0	0.008	0	0.008	0.008	0	0.004	0	0	0	0	0.004	0	0	0
Thraupidae	*Pipraeidea melanonota*	0.441	0.003	0	0.003	0	0.510	0.073	0.073	0.073	0.073	0.310	0.625	0.625	0.625	0.625	0.480	0.681	0.681	0.681	0.681
Thraupidae	*Piranga leucoptera*	0	0.085	0	0	0	0.963	0.522	0	0.522	0	0.860	0.965	0.965	0.965	0.965	0	0.294	0	0.294	0.294
Thraupidae	*Ramphocelus carbo*	0.188	0.017	0	0.017	0	0.366	0.343	0.343	0.343	0.343	0.103	0.053	0.053	0.053	0.053	0	0.134	0	0	0
Thraupidae	*Ramphocelus nigrogularis*	0.027	0.010	0	0.010	0	0.070	0.861	0.861	0.861	0.861	0	0.015	0	0	0	0	0.014	0	0	0
Thraupidae	*Schistochlamys melanopis*	0	0.699	0	0	0	0.931	0.978	0.978	0.978	0.978	0.239	0.958	0.958	0.958	0.958	0.044	0.061	0.061	0.061	0.061
Thraupidae	*Tachyphonus cristatus*	0	0.340	0	0.340	0	0	0.329	0	0	0	0	0.195	0	0	0	0	0.009	0	0	0
Thraupidae	*Tachyphonus rufiventer*	0.676	0.052	0	0.052	0	0.982	0.993	0.993	0.993	0.993	0	0.974	0	0.974	0.974	0	0.847	0	0	0
Thraupidae	*Tangara arthus*	0	0.003	0	0	0	0.985	0.884	0.884	0.884	0.884	0.636	0.832	0.832	0.832	0.832	0	0.955	0	0.955	0.955
Thraupidae	*Tangara callophrys*	0.830	0.011	0	0.011	0	0	0.896	0	0.896	0.896	0	0.019	0	0	0	0	0.008	0	0	0
Thraupidae	*Tangara chilensis*	0.278	0.036	0	0.036	0	0.976	0.935	0.935	0.935	0.935	0.243	0.847	0.847	0.847	0.847	0	0.205	0	0.205	0.205
Thraupidae	*Tangara chrysotis*	0	0.046	0	0	0	0.986	0.984	0	0.984	0	0.779	0.441	0.441	0.441	0.441	0.154	0.928	0.928	0.928	0.928
Thraupidae	*Tangara cyanicollis*	0.548	0.006	0	0.006	0	0.988	0.731	0.731	0.731	0.731	0.749	0.782	0.782	0.782	0.782	0.724	0.993	0.993	0.993	0.993
Thraupidae	*Tangara cyanotis*	0	0.010	0	0	0	0	0.080	0	0	0	0.932	0.943	0	0.943	0	0.948	0.926	0.926	0.926	0.926
Thraupidae	*Tangara gyrola*	0.419	0.006	0	0.006	0	0.992	0.827	0.827	0.827	0.827	0.898	0.991	0.991	0.991	0.991	0	0.995	0	0	0
Thraupidae	*Tangara mexicana*	0.389	0.154	0	0.154	0	0.243	0.971	0.971	0.971	0.971	0	0.071	0	0	0	0	0.027	0	0	0
Thraupidae	*Tangara nigrocincta*	0.978	0.066	0	0.066	0	0.564	0.950	0.950	0.950	0.950	0	0.892	0	0	0	0	0.672	0	0	0
Thraupidae	*Tangara nigroviridis*	0	0.004	0	0	0	0	0.717	0	0	0	0.962	0.745	0	0.745	0	0.992	0.165	0.165	0.165	0.165
Thraupidae	*Tangara parzudakii*	0	0.003	0	0	0	0	0.556	0	0	0	0.995	0.625	0	0.625	0	0.991	0.732	0.732	0.732	0.732
Thraupidae	*Tangara punctata*	0	0.043	0	0	0	0.989	0.955	0.955	0.955	0.955	0.427	0.952	0.952	0.952	0.952	0.277	0.761	0.761	0.761	0.761
Thraupidae	*Tangara ruficervix*	0	0.005	0	0	0	0.978	0.084	0	0.084	0	0.987	0.636	0.636	0.636	0.636	0.991	0.136	0.136	0.136	0.136
Thraupidae	*Tangara schrankii*	0.203	0.025	0	0.025	0	0.231	0.742	0.742	0.742	0.742	0	0.980	0	0	0	0	0.064	0	0	0
Thraupidae	*Tangara vassorii*	0	0.009	0	0	0	0	0.167	0	0	0	0	0.337	0	0	0	0.545	0.119	0	0.119	0
Thraupidae	*Tangara velia*	0.926	0.487	0	0.487	0	0	0.365	0	0	0	0	0.008	0	0	0	0	0.038	0	0	0
Thraupidae	*Tangara viridicollis*	0	0.014	0	0	0	0.066	0.002	0	0.002	0	0.023	0.196	0.196	0.196	0.196	0.133	0.077	0.077	0.077	0.077
Thraupidae	*Tangara xanthocephala*	0	0.002	0	0	0	0.871	0.797	0	0.797	0	0.459	0.958	0.958	0.958	0.958	0.974	0.167	0.167	0.167	0.167
Thraupidae	*Tangara xanthogastra*	0.583	0.055	0	0.055	0	0.926	0.979	0.979	0.979	0.979	0	0.989	0	0.989	0.989	0	0.287	0	0	0
Thraupidae	*Tersina viridis*	0.590	0.065	0	0.065	0	0.994	0.427	0.427	0.427	0.427	0	0.679	0	0.679	0.679	0	0.971	0	0	0
Thraupidae	*Thlypopsis sordida*	0.741	0.123	0	0.123	0	0	0.914	0	0	0	0	0.184	0	0	0	0	0.126	0	0	0
Thraupidae	*Thraupis bonariensis*	0	0.002	0	0	0	0.002	0.002	0	0.002	0	0.006	0.089	0.089	0.089	0.089	0.183	0.020	0.020	0.020	0.020
Thraupidae	*Thraupis cyanocephala*	0	0.002	0	0	0	0	0.304	0	0	0	0.794	0.263	0	0.263	0	0.938	0.743	0.743	0.743	0.743
Thraupidae	*Thraupis episcopus*	0.109	0.267	0	0.267	0	0.267	0.549	0.549	0.549	0.549	0.458	0.299	0.299	0.299	0.299	0	0.430	0	0	0

Thraupidae	*Thraupis palmarum*	0.092	0.296	0	0.296	0	0.307	0.705	0.705	0.705	0.705	0.321	0.348	0.348	0.348	0.348	0	0.448	0	0	0
Thraupidae	*Trichothraupis melanops*	0	0.003	0	0	0	0.010	0.003	0	0.003	0	0.010	0.726	0.726	0.726	0.726	0	0.003	0	0.003	0.003
Tinamidae	*Crypturellus atrocapillus*	0.449	0.012	0	0.012	0	0.963	0.992	0.992	0.992	0.992	0	0.986	0	0	0	0	0.454	0	0	0
Tinamidae	*Crypturellus bartletti*	0.007	0.037	0	0.037	0	0	0.020	0	0	0	0	0.009	0	0	0	0	0.005	0	0	0
Tinamidae	*Crypturellus cinereus*	0.006	0.004	0	0.004	0	0.001	0.132	0.132	0.132	0.132	0	0.002	0	0	0	0	0.001	0	0	0
Tinamidae	*Crypturellus obsoletus*	0.021	0.002	0	0.002	0	0.861	0.483	0.483	0.483	0.483	0.006	0.946	0.946	0.946	0.946	0.010	0.689	0.689	0.689	0.689
Tinamidae	*Crypturellus soui*	0.273	0.451	0	0.451	0	0.893	0.992	0.992	0.992	0.992	0.518	0.779	0.779	0.779	0.779	0	0.925	0	0	0
Tinamidae	*Crypturellus strigulosus*	0.081	0.013	0	0.013	0	0	0.003	0	0.003	0.003	0	0.001	0	0	0	0	0.000	0	0	0
Tinamidae	*Crypturellus undulatus*	0.015	0.004	0	0.004	0	0	0.193	0	0.193	0.193	0	0.004	0	0	0	0	0.004	0	0	0
Tinamidae	*Crypturellus variegatus*	0.017	0.008	0	0.008	0	0	0.455	0	0	0	0	0.003	0	0	0	0	0.004	0	0	0
Tinamidae	*Nothocercus nigrocapillus*	0	0.003	0	0	0	0	0.003	0	0	0	0	0.003	0	0	0	0.706	0.006	0.006	0.006	0.006
Tinamidae	*Tinamus guttatus*	0.092	0.379	0	0.379	0	0.002	0.865	0.865	0.865	0.865	0	0.030	0	0	0	0	0.006	0	0	0
Tinamidae	*Tinamus major*	0.668	0.223	0	0.223	0	0	0.079	0	0.079	0.079	0	0.516	0	0	0	0	0.035	0	0	0
Tinamidae	*Tinamus tao*	0.905	0.014	0	0.014	0	0.875	0.967	0.967	0.967	0.967	0	0.974	0	0.974	0.974	0	0.834	0	0	0
Trogonidae	*Pharomachrus antisianus*	0	0.053	0	0	0	0.993	0.517	0	0.517	0	0.803	0.988	0.988	0.988	0.988	0.936	0.969	0.969	0.969	0.969
Trogonidae	*Pharomachrus auriceps*	0	0.002	0	0	0	0	0.160	0	0	0	0.931	0.963	0	0.963	0	0.989	0.941	0.941	0.941	0.941
Trogonidae	*Pharomachrus pavoninus*	0.918	0.837	0	0.837	0	0.132	0.837	0.837	0.837	0.837	0	0.130	0	0.130	0.130	0	0.010	0	0	0
Trogonidae	*Trogon collaris*	0.004	0.023	0	0.023	0	0.965	0.681	0.681	0.681	0.681	0	0.600	0	0.600	0.600	0	0.874	0	0	0
Trogonidae	*Trogon curucui*	0.058	0.024	0	0.024	0	0.181	0.593	0.593	0.593	0.593	0.004	0.198	0.198	0.198	0.198	0	0.044	0	0	0
Trogonidae	*Trogon melanurus*	0.007	0.157	0	0.157	0	0.004	0.820	0.820	0.820	0.820	0	0.004	0	0	0	0	0.004	0	0	0
Trogonidae	*Trogon personatus*	0	0.011	0	0	0	0	0.936	0	0	0	0.712	0.737	0.737	0.737	0.737	0.908	0.975	0.975	0.975	0.975
Trogonidae	*Trogon violaceus*	0.694	0.870	0	0.870	0	0	0.596	0	0.596	0.596	0	0.297	0	0	0	0	0.054	0	0	0
Trogonidae	*Trogon viridis*	0.243	0.024	0	0.024	0	0	0.165	0	0.165	0.165	0	0.117	0	0	0	0	0.028	0	0	0
Turdidae	*Catharus fuscater*	0	0.008	0	0	0	0	0.007	0	0	0	0.978	0.135	0	0.135	0	0.549	0.531	0.531	0.531	0.531
Turdidae	*Entomodestes leucotis*	0	0.012	0	0	0	0	0.215	0	0	0	0.473	0.473	0	0.473	0	0.444	0.478	0.478	0.478	0.478
Turdidae	*Myadestes ralloides*	0	0.002	0	0	0	0.990	0.272	0	0.272	0	0.940	0.978	0.978	0.978	0.978	0.961	0.771	0.771	0.771	0.771
Turdidae	*Turdus albicollis*	0.068	0.024	0	0.024	0	0	0.552	0	0.552	0.552	0	0.858	0	0	0	0	0.258	0	0	0
Turdidae	*Turdus chiguanco*	0	0.001	0	0	0	0	0.004	0	0	0	0.001	0.003	0	0.003	0	0.001	0.001	0.001	0.001	0.001
Turdidae	*Turdus fuscater*	0	0.004	0	0	0	0	0.398	0	0	0	0	0.188	0	0	0	0	0.074	0	0	0
Turdidae	*Turdus hauxwelli*	0.037	0.041	0	0.041	0	0.125	0.932	0.932	0.932	0.932	0	0.091	0	0.091	0.091	0	0.046	0	0	0
Turdidae	*Turdus ignobilis*	0.332	0.025	0	0.025	0	0.985	0.654	0.654	0.654	0.654	0.052	0.943	0.943	0.943	0.943	0	0.923	0	0.923	0.923
Turdidae	*Turdus leucops*	0	0.031	0	0	0	0.919	0.450	0	0.450	0	0.753	0.881	0.881	0.881	0.881	0.902	0.592	0.592	0.592	0.592
Turdidae	*Turdus nigriceps*	0.592	0.002	0	0.002	0	0.873	0.711	0.711	0.711	0.711	0.116	0.678	0.678	0.678	0.678	0	0.819	0	0.819	0.819
Turdidae	*Turdus serranus*	0	0.005	0	0	0	0	0.394	0	0	0	0.173	0.752	0	0.752	0	0.986	0.687	0.687	0.687	0.687
Tyrannidae	*Elaenia albiceps*	0.042	0.003	0	0.003	0	0.005	0.003	0.003	0.003	0.003	0.004	0.082	0.082	0.082	0.082	0.722	0.005	0.005	0.005	0.005
Tyrannidae	*Elaenia flavogaster*	0.010	0.096	0	0.096	0	0	0.975	0	0.975	0.975	0	0.960	0	0	0	0	0.917	0	0	0
Tyrannidae	*Elaenia gigas*	0.992	0.314	0	0.314	0	0.991	0.995	0.995	0.995	0.995	0.364	0.980	0.980	0.980	0.980	0	0.580	0	0	0
Tyrannidae	*Elaenia obscura*	0	0.017	0	0	0	0	0.023	0	0	0	0	0.093	0	0	0	0.298	0.194	0	0.194	0
Tyrannidae	*Elaenia pallatangae*	0	0.013	0	0	0	0	0.979	0	0	0	0.951	0.421	0.421	0.421	0.421	0.994	0.248	0.248	0.248	0.248
Tyrannidae	*Lophotriccus pileatus*	0	0.006	0	0	0	0.948	0.956	0	0.956	0	0.988	0.989	0.989	0.989	0.989	0	0.986	0	0.986	0.986
Tyrannidae	*Mionectes macconnelli*	0.003	0.013	0	0.013	0	0.499	0.984	0.984	0.984	0.984	0	0.478	0	0.478	0.478	0	0.251	0	0	0
Tyrannidae	*Mionectes oleagineus*	0.335	0.192	0	0.192	0	0.958	0.995	0.995	0.995	0.995	0	0.989	0	0.989	0.989	0	0.567	0	0	0
Tyrannidae	*Mionectes olivaceus*	0.547	0.043	0	0.043	0	0.996	0.507	0.507	0.507	0.507	0.743	0.997	0.997	0.997	0.997	0	0.816	0	0.816	0.816
Tyrannidae	*Mionectes striaticollis*	0	0.004	0	0	0	0.325	0.724	0.724	0.724	0.724	0.263	0.847	0.847	0.847	0.847	0.994	0.562	0.562	0.562	0.562
Tyrannidae	*Zimmerius bolivianus*	0	0.003	0	0	0	0.956	0.209	0	0.209	0	0.737	0.926	0.926	0.926	0.926	0.385	0.912	0.912	0.912	0.912
Tyrannidae	*Zimmerius gracilipes*	0.036	0.030	0	0.030	0	0	0.448	0	0.448	0.448	0	0.042	0	0	0	0	0.339	0	0	0

Table S3. *Continued.*

		2500 m					3000 m					3500 m				
Family	Bird species	current	no range change	contraction	expansion	shift	current	no range change	contraction	expansion	shift	current	no range change	contraction	expansion	shift
Cardinalidae	*Cyanocompsa cyanoides*	0	0.093	0	0	0	0	0.049	0	0	0	0	0.004	0	0	0
Cardinalidae	*Pheucticus aureoventris*	0.007	0.013	0.013	0.013	0.013	0.043	0.007	0.007	0.007	0.007	0	0.003	0	0.003	0.003
Cardinalidae	*Pheucticus chrysogaster*	0.004	0.052	0.052	0.052	0.052	0.004	0.064	0.064	0.064	0.064	0.010	0.004	0.004	0.004	0.004
Cardinalidae	*Saltator aurantiirostris*	0	0.002	0	0	0	0.002	0.002	0.002	0.002	0.002	0.003	0.004	0.004	0.004	0.004
Cardinalidae	*Saltator coerulescens*	0	0.586	0	0	0	0	0.004	0	0	0	0	0.004	0	0	0
Cardinalidae	*Saltator grossus*	0	0.030	0	0	0	0	0.003	0	0	0	0	0.003	0	0	0
Cardinalidae	*Saltator maximus*	0.007	0.168	0.168	0.168	0.168	0	0.006	0	0	0	0	0.005	0	0	0
Columbidae	*Geotrygon montana*	0	0.041	0	0	0	0	0.125	0	0	0	0	0.001	0	0	0
Columbidae	*Patagioenas cayennensis*	0	0.008	0	0	0	0	0.085	0	0	0	0	0.005	0	0	0
Columbidae	*Patagioenas fasciata*	0.969	0.796	0.796	0.796	0.796	0.090	0.296	0.296	0.296	0.296	0.005	0.170	0.170	0.170	0.170
Columbidae	*Patagioenas plumbea*	0	0.102	0	0	0	0	0.225	0	0	0	0	0.003	0	0	0
Columbidae	*Patagioenas subvinacea*	0	0.868	0	0	0	0	0.023	0	0	0	0	0.008	0	0	0
Corvidae	*Cyanocorax cyanomelas*	0	0.003	0	0	0	0	0.003	0	0	0	0	0.003	0	0	0
Corvidae	*Cyanocorax violaceus*	0	0.039	0	0	0	0	0.003	0	0	0	0	0.003	0	0	0
Corvidae	*Cyanocorax yncas*	0	0.994	0	0.994	0.994	0	0.547	0	0	0	0	0.044	0	0	0
Cotingidae	*Ampelioides tschudii*	0	0.255	0	0	0	0	0.048	0	0	0	0	0.010	0	0	0
Cotingidae	*Ampelion rubrocristatus*	0.903	0.939	0	0.939	0	0.015	0.291	0.291	0.291	0.291	0	0.068	0	0.068	0.068
Cotingidae	*Ampelion rufaxilla*	0.856	0.884	0.884	0.884	0.884	0	0.940	0	0.940	0.940	0	0.071	0	0	0
Cotingidae	*Cephalopterus ornatus*	0	0.084	0	0	0	0	0.004	0	0	0	0	0.004	0	0	0
Cotingidae	*Conioptilon mcilhennyi*	0	0.078	0	0	0	0	0.062	0	0	0	0	0.089	0	0	0
Cotingidae	*Cotinga cayana*	0	0.033	0	0	0	0	0.005	0	0	0	0	0.007	0	0	0
Cotingidae	*Cotinga maynana*	0	0.000	0	0	0	0	0.000	0	0	0	0	0.000	0	0	0
Cotingidae	*Gymnoderus foetidus*	0	0.002	0	0	0	0	0.010	0	0	0	0	0.002	0	0	0
Cotingidae	*Iodopleura isabellae*	0	0.002	0	0	0	0	0.002	0	0	0	0	0.001	0	0	0
Cotingidae	*Laniocera hypopyrra*	0	0.015	0	0	0	0	0.015	0	0	0	0	0.015	0	0	0
Cotingidae	*Lipaugus vociferans*	0	0.002	0	0	0	0	0.002	0	0	0	0	0.006	0	0	0
Cotingidae	*Oxyruncus cristatus*	0	0.538	0	0	0	0	0.520	0	0	0	0	0.019	0	0	0
Cotingidae	*Pipreola arcuata*	0.612	0.915	0.915	0.915	0.915	0.356	0.932	0.932	0.932	0.932	0.189	0.992	0.992	0.992	0.992
Cotingidae	*Pipreola frontalis*	0	0.508	0	0	0	0	0.072	0	0	0	0	0.023	0	0	0
Cotingidae	*Pipreola intermedia*	0.095	0.037	0.037	0.037	0.037	0.302	0.969	0.969	0.969	0.969	0	0.055	0	0	0
Cotingidae	*Porphyrolaema porphyrolaema*	0	0.001	0	0	0	0	0.000	0	0	0	0	0.000	0	0	0
Cotingidae	*Querula purpurata*	0	0.001	0	0	0	0	0.003	0	0	0	0	0.001	0	0	0
Cotingidae	*Rupicola peruvianus*	0.733	0.946	0.946	0.946	0.946	0	0.006	0	0.006	0.006	0	0.213	0	0	0
Cotingidae	*Snowornis subalaris*	0	0.010	0	0	0	0	0.010	0	0	0	0	0.010	0	0	0
Cotingidae	*Tityra cayana*	0	0.006	0	0	0	0	0.008	0	0	0	0	0.002	0	0	0
Cotingidae	*Tityra inquisitor*	0	0.014	0	0	0	0	0.013	0	0	0	0	0.003	0	0	0
Cotingidae	*Tityra semifasciata*	0	0.806	0	0	0	0	0.006	0	0	0	0	0.007	0	0	0
Cracidae	*Aburria aburri*	0	0.733	0	0	0	0	0.084	0	0	0	0	0.009	0	0	0
Cracidae	*Mitu tuberosum*	0	0.001	0	0	0	0	0.001	0	0	0	0	0.001	0	0	0
Cracidae	*Ortalis guttata*	0	0.232	0	0	0	0	0.089	0	0	0	0	0.005	0	0	0
Cracidae	*Penelope jacquacu*	0	0.003	0	0	0	0	0.003	0	0	0	0	0.003	0	0	0

Cracidae	*Penelope montagnii*	0.624	0.877	0.877	0.877	0.877	0.151	0.705	0.705	0.705	0.705	0	0.033	0	0.033	0.033
Cracidae	*Pipile cumanensis*	0	0.005	0	0	0	0	0.005	0	0	0	0	0.007	0	0	0
Icteridae	*Cacicus cela*	0	0.299	0	0	0	0	0.005	0	0	0	0	0.005	0	0	0
Icteridae	*Cacicus chrysonotus*	0.874	0.957	0.957	0.957	0.957	0.295	0.058	0.058	0.058	0.058	0	0.163	0	0.163	0.163
Icteridae	*Cacicus haemorrhous*	0	0.014	0	0	0	0	0.013	0	0	0	0	0.003	0	0	0
Icteridae	*Clypicterus oseryi*	0	0.018	0	0	0	0	0.002	0	0	0	0	0.006	0	0	0
Icteridae	*Icterus icterus*	0	0.030	0	0	0	0	0.025	0	0	0	0	0.024	0	0	0
Icteridae	*Molothrus oryzivorus*	0	0.246	0	0	0	0	0.016	0	0	0	0	0.045	0	0	0
Icteridae	*Psarocolius angustifrons*	0	0.868	0	0	0	0	0.008	0	0	0	0	0.005	0	0	0
Icteridae	*Psarocolius atrovirens*	0.009	0.208	0.208	0.208	0.208	0	0.015	0	0.015	0.015	0	0.001	0	0	0
Icteridae	*Psarocolius bifasciatus*	0	0.002	0	0	0	0	0.002	0	0	0	0	0.002	0	0	0
Icteridae	*Psarocolius decumanus*	0	0.574	0	0	0	0	0.026	0	0	0	0	0.004	0	0	0
Odontophoridae	*Odontophorus balliviani*	0.475	0.631	0.631	0.631	0.631	0.411	0.568	0.568	0.568	0.568	0	0.004	0	0	0
Odontophoridae	*Odontophorus speciosus*	0	0.359	0	0	0	0	0.136	0	0	0	0	0.009	0	0	0
Odontophoridae	*Odontophorus stellatus*	0	0.039	0	0	0	0	0.001	0	0	0	0	0.001	0	0	0
Picidae	*Celeus elegans*	0	0.001	0	0	0	0	0.001	0	0	0	0	0.001	0	0	0
Picidae	*Celeus flavus*	0	0.008	0	0	0	0	0.008	0	0	0	0	0.008	0	0	0
Picidae	*Celeus grammicus*	0	0.007	0	0	0	0	0.006	0	0	0	0	0.016	0	0	0
Picidae	*Celeus spectabilis*	0	0.206	0	0	0	0	0.000	0	0	0	0	0.001	0	0	0
Picidae	*Celeus torquatus*	0	0.012	0	0	0	0	0.009	0	0	0	0	0.011	0	0	0
Picidae	*Colaptes rubiginosus*	0	0.954	0	0	0	0	0.027	0	0	0	0	0.005	0	0	0
Pipridae	*Chiroxiphia boliviana*	0	0.012	0	0	0	0	0.008	0	0	0	0	0.006	0	0	0
Pipridae	*Chiroxiphia pareola*	0	0.029	0	0	0	0	0.032	0	0	0	0	0.027	0	0	0
Pipridae	*Lepidothrix coeruleocapilla*	0	0.004	0	0	0	0	0.004	0	0	0	0	0.003	0	0	0
Pipridae	*Lepidothrix coronata*	0	0.013	0	0	0	0	0.011	0	0	0	0	0.013	0	0	0
Pipridae	*Machaeropterus pyrocephalus*	0	0.004	0	0	0	0	0.004	0	0	0	0	0.004	0	0	0
Pipridae	*Manacus manacus*	0	0.963	0	0	0	0	0.002	0	0	0	0	0.002	0	0	0
Pipridae	*Neopelma sulphureiventer*	0	0.031	0	0	0	0	0.042	0	0	0	0	0.056	0	0	0
Pipridae	*Pipra chloromeros*	0	0.006	0	0	0	0	0.006	0	0	0	0	0.006	0	0	0
Pipridae	*Pipra fasciicauda*	0	0.003	0	0	0	0	0.003	0	0	0	0	0.003	0	0	0
Pipridae	*Xenopipo holochlora*	0	0.015	0	0	0	0	0.021	0	0	0	0	0.001	0	0	0
Pipridae	*Xenopipo unicolor*	0	0.317	0	0	0	0	0.012	0	0	0	0	0.003	0	0	0
Psittacidae	*Amazona farinosa*	0	0.011	0	0	0	0	0.010	0	0	0	0	0.004	0	0	0
Psittacidae	*Amazona mercenaria*	0.981	0.981	0.981	0.981	0.981	0.948	0.759	0.759	0.759	0.759	0	0.838	0	0	0
Psittacidae	*Amazona ochrocephala*	0	0.004	0	0	0	0	0.009	0	0	0	0	0.005	0	0	0
Psittacidae	*Ara ararauna*	0	0.005	0	0	0	0	0.005	0	0	0	0	0.005	0	0	0
Psittacidae	*Ara chloropterus*	0	0.003	0	0	0	0	0.005	0	0	0	0	0.005	0	0	0
Psittacidae	*Ara macao*	0	0.003	0	0	0	0	0.003	0	0	0	0	0.003	0	0	0
Psittacidae	*Ara militaris*	0	0.213	0	0	0	0	0.001	0	0	0	0	0.001	0	0	0
Psittacidae	*Ara severus*	0	0.044	0	0	0	0	0.006	0	0	0	0	0.004	0	0	0
Psittacidae	*Aratinga leucophthalma*	0	0.003	0	0	0	0	0.130	0	0	0	0	0.004	0	0	0
Psittacidae	*Aratinga mitrata*	0.002	0.002	0.002	0.002	0.002	0.002	0.002	0.002	0.002	0.002	0	0.002	0	0.002	0.002
Psittacidae	*Aratinga weddellii*	0	0.002	0	0	0	0	0.002	0	0	0	0	0.002	0	0	0
Psittacidae	*Bolborhynchus lineola*	0.715	0.752	0.752	0.752	0.752	0.195	0.566	0.566	0.566	0.566	0	0.157	0	0.157	0.157
Psittacidae	*Bolborhynchus orbygnesius*	0.049	0.107	0	0.107	0	0.029	0.051	0.051	0.051	0.051	0.012	0.015	0.015	0.015	0.015
Psittacidae	*Brotogeris cyanoptera*	0	0.007	0	0	0	0	0.002	0	0	0	0	0.002	0	0	0
Psittacidae	*Brotogeris sanctithomae*	0	0.028	0	0	0	0	0.002	0	0	0	0	0.028	0	0	0
Psittacidae	*Forpus modestus*	0	0.017	0	0	0	0	0.017	0	0	0	0	0.017	0	0	0
Psittacidae	*Nannopsittaca dachilleae*	0	0.001	0	0	0	0	0.000	0	0	0	0	0.000	0	0	0
Psittacidae	*Orthopsittaca manilata*	0	0.023	0	0	0	0	0.004	0	0	0	0	0.007	0	0	0

Psittacidae	*Pionites leucogaster*	0	0.001	0	0	0	0	0.000	0	0	0	0	0.000	0	0	0
Psittacidae	*Pionus menstruus*	0	0.092	0	0.092	0.092	0	0.004	0	0	0	0	0.004	0	0	0
Psittacidae	*Pionus tumultuosus*	0.893	0.990	0.990	0.990	0.990	0.297	0.990	0.990	0.990	0.990	0	0.029	0	0	0
Psittacidae	*Primolius couloni*	0	0.004	0	0	0	0	0.011	0	0	0	0	0.006	0	0	0
Psittacidae	*Pyrilia barrabandi*	0	0.006	0	0	0	0	0.006	0	0	0	0	0.006	0	0	0
Psittacidae	*Pyrrhura roseifrons*	0	0.005	0	0	0	0	0.005	0	0	0	0	0.005	0	0	0
Psittacidae	*Pyrrhura rupicola*	0	0.018	0	0	0	0	0.018	0	0	0	0	0.018	0	0	0
Psittacidae	*Touit huetii*	0	0.001	0	0	0	0	0.000	0	0	0	0	0.000	0	0	0
Psophiidae	*Psophia leucoptera*	0	0.022	0	0	0	0	0.002	0	0	0	0	0.006	0	0	0
Ramphastidae	*Andigena hypoglauca*	0.230	0.047	0.047	0.047	0.047	0.767	0.928	0.928	0.928	0.928	0.814	0.200	0.200	0.200	0.200
Ramphastidae	*Aulacorhynchus coeruleicinctis*	0.043	0.671	0.671	0.671	0.671	0	0.885	0	0.885	0.885	0	0.012	0	0	0
Ramphastidae	*Aulacorhynchus derbianus*	0	0.425	0	0	0	0	0.167	0	0	0	0	0.019	0	0	0
Ramphastidae	*Aulacorhynchus prasinus*	0	0.915	0	0	0	0	0.522	0	0	0	0	0.316	0	0	0
Ramphastidae	*Capito auratus*	0	0.019	0	0	0	0	0.001	0	0	0	0	0.002	0	0	0
Ramphastidae	*Eubucco richardsoni*	0	0.002	0	0	0	0	0.003	0	0	0	0	0.002	0	0	0
Ramphastidae	*Eubucco tucinkae*	0	0.086	0	0	0	0	0.004	0	0	0	0	0.005	0	0	0
Ramphastidae	*Eubucco versicolor*	0	0.007	0	0.007	0.007	0	0.050	0	0	0	0	0.002	0	0	0
Ramphastidae	*Pteroglossus azara*	0	0.001	0	0	0	0	0.001	0	0	0	0	0.002	0	0	0
Ramphastidae	*Pteroglossus beauharnaesii*	0	0.003	0	0	0	0	0.003	0	0	0	0	0.003	0	0	0
Ramphastidae	*Pteroglossus castanotis*	0	0.033	0	0	0	0	0.004	0	0	0	0	0.004	0	0	0
Ramphastidae	*Pteroglossus inscriptus*	0	0.014	0	0	0	0	0.014	0	0	0	0	0.013	0	0	0
Ramphastidae	*Ramphastos tucanus*	0	0.002	0	0	0	0	0.002	0	0	0	0	0.003	0	0	0
Ramphastidae	*Ramphastos vitellinus*	0	0.009	0	0	0	0	0.003	0	0	0	0	0.004	0	0	0
Ramphastidae	*Selenidera reinwardtii*	0	0.003	0	0	0	0	0.000	0	0	0	0	0.000	0	0	0
Steatornithidae	*Steatornis caripensis*	0	0.989	0	0	0	0	0.798	0	0	0	0	0.130	0	0	0
Thraupidae	*Anisognathus igniventris*	0	0.222	0	0	0	0.551	0.940	0.940	0.940	0.940	0.878	0.767	0.767	0.767	0.767
Thraupidae	*Anisognathus somptuosus*	0.130	0.831	0.831	0.831	0.831	0.051	0.009	0.009	0.009	0.009	0	0.258	0	0	0
Thraupidae	*Buthraupis montana*	0.370	0.864	0	0.864	0	0.040	0.232	0.232	0.232	0.232	0	0.509	0	0.509	0.509
Thraupidae	*Catamblyrhynchus diadema*	0.921	0.940	0.940	0.940	0.940	0.864	0.945	0.945	0.945	0.945	0	0.612	0	0.612	0.612
Thraupidae	*Chlorochrysa calliparaea*	0	0.717	0	0.717	0.717	0	0.063	0	0	0	0	0.005	0	0	0
Thraupidae	*Chlorophanes spiza*	0	0.089	0	0	0	0	0.005	0	0	0	0	0.006	0	0	0
Thraupidae	*Chlorophonia cyanea*	0	0.823	0	0	0	0	0.045	0	0	0	0	0.007	0	0	0
Thraupidae	*Chlorornis riefferii*	0.813	0.884	0.884	0.884	0.884	0.561	0.991	0.991	0.991	0.991	0	0.015	0	0.015	0.015
Thraupidae	*Chlorospingus canigularis*	0	0.960	0	0	0	0	0.773	0	0	0	0	0.005	0	0	0
Thraupidae	*Chlorospingus flavigularis*	0	0.076	0	0.076	0.076	0	0.059	0	0	0	0	0.003	0	0	0
Thraupidae	*Chlorospingus ophthalmicus*	0.776	0.919	0.919	0.919	0.919	0	0.971	0	0	0	0	0.127	0	0	0
Thraupidae	*Chlorospingus parvirostris*	0.414	0.934	0.934	0.934	0.934	0	0.499	0	0	0	0	0.008	0	0	0
Thraupidae	*Cissopis leverianus*	0	0.225	0	0	0	0	0.011	0	0	0	0	0.006	0	0	0
Thraupidae	*Conirostrum cinereum*	0	0.012	0	0	0	0.003	0.012	0	0.012	0	0.014	0.003	0.003	0.003	0.003
Thraupidae	*Conirostrum speciosum*	0	0.018	0	0	0	0	0.024	0	0	0	0	0.008	0	0	0
Thraupidae	*Cyanerpes caeruleus*	0	0.260	0	0	0	0	0.002	0	0	0	0	0.006	0	0	0
Thraupidae	*Cyanerpes cyaneus*	0	0.389	0	0	0	0	0.000	0	0	0	0	0.000	0	0	0
Thraupidae	*Dacnis cayana*	0	0.030	0	0	0	0	0.015	0	0	0	0	0.005	0	0	0
Thraupidae	*Dacnis flaviventer*	0	0.190	0	0	0	0	0.002	0	0	0	0	0.004	0	0	0
Thraupidae	*Dacnis lineata*	0	0.487	0	0	0	0	0.004	0	0	0	0	0.004	0	0	0
Thraupidae	*Delothraupis castaneoventris*	0	0.001	0	0.001	0	0.070	0.080	0.080	0.080	0.080	0	0.007	0.007	0.007	0.007
Thraupidae	*Diglossa brunneiventris*	0	0.002	0	0	0	0.003	0.006	0.006	0.006	0.006	0.007	0.063	0.063	0.063	0.063
Thraupidae	*Diglossa caerulescens*	0.977	0.997	0.997	0.997	0.997	0	0.346	0	0.346	0.346	0	0.078	0	0	0
Thraupidae	*Diglossa cyanea*	0.969	0.280	0.280	0.280	0.280	0.044	0.653	0.653	0.653	0.653	0.125	0.042	0.042	0.042	0.042
Thraupidae	*Diglossa glauca*	0	0.673	0	0.673	0.673	0	0.073	0	0	0	0	0.018	0	0	0

Thraupidae	*Diglossa mystacalis*	0	0.005	0	0	0	0.185	0.091	0.091	0.091	0.091	0.033	0.011	0.011	0.011	0.011
Thraupidae	*Diglossa sittoides*	0.216	0.711	0.711	0.711	0.711	0.062	0.682	0.682	0.682	0.682	0.004	0.005	0.005	0.005	0.005
Thraupidae	*Dubusia taeniata*	0.863	0.657	0.657	0.657	0.657	0.374	0.156	0.156	0.156	0.156	0	0.971	0	0.971	0.971
Thraupidae	*Euphonia chlorotica*	0	0.009	0	0	0	0	0.011	0	0	0	0	0.004	0	0	0
Thraupidae	*Euphonia chrysopasta*	0	0.042	0	0	0	0	0.017	0	0	0	0	0.028	0	0	0
Thraupidae	*Euphonia laniirostris*	0	0.511	0	0	0	0	0.005	0	0	0	0	0.119	0	0	0
Thraupidae	*Euphonia mesochrysa*	0	0.797	0	0	0	0	0.017	0	0	0	0	0.005	0	0	0
Thraupidae	*Euphonia minuta*	0	0.100	0	0	0	0	0.020	0	0	0	0	0.031	0	0	0
Thraupidae	*Euphonia rufiventris*	0	0.019	0	0	0	0	0.026	0	0	0	0	0.012	0	0	0
Thraupidae	*Euphonia xanthogaster*	0	0.219	0	0.219	0.219	0	0.080	0	0	0	0	0.011	0	0	0
Thraupidae	*Iridophanes pulcherrimus*	0	0.998	0	0	0	0	0.820	0	0	0	0	0.001	0	0	0
Thraupidae	*Iridosornis analis*	0	0.513	0	0.513	0.513	0	0.023	0	0	0	0	0.022	0	0	0
Thraupidae	*Iridosornis jelskii*	0.003	0.056	0	0.056	0	0.195	0.144	0.144	0.144	0.144	0.003	0.005	0.005	0.005	0.005
Thraupidae	*Lamprospiza melanoleuca*	0	0.004	0	0	0	0	0.004	0	0	0	0	0.004	0	0	0
Thraupidae	*Pipraeidea melanonota*	0.273	0.790	0.790	0.790	0.790	0.007	0.677	0.677	0.677	0.677	0	0.036	0	0	0
Thraupidae	*Piranga leucoptera*	0	0.455	0	0	0	0	0.014	0	0	0	0	0.041	0	0	0
Thraupidae	*Ramphocelus carbo*	0	0.027	0	0	0	0	0.003	0	0	0	0	0.003	0	0	0
Thraupidae	*Ramphocelus nigrogularis*	0	0.005	0	0	0	0	0.002	0	0	0	0	0.003	0	0	0
Thraupidae	*Schistochlamys melanopis*	0.010	0.011	0.011	0.011	0.011	0	0.099	0	0	0	0	0.009	0	0	0
Thraupidae	*Tachyphonus cristatus*	0	0.005	0	0	0	0	0.004	0	0	0	0	0.003	0	0	0
Thraupidae	*Tachyphonus rufiventer*	0	0.003	0	0	0	0	0.005	0	0	0	0	0.004	0	0	0
Thraupidae	*Tangara arthus*	0	0.127	0	0	0	0	0.003	0	0	0	0	0.298	0	0	0
Thraupidae	*Tangara callophrys*	0	0.006	0	0	0	0	0.007	0	0	0	0	0.006	0	0	0
Thraupidae	*Tangara chilensis*	0	0.855	0	0	0	0	0.003	0	0	0	0	0.003	0	0	0
Thraupidae	*Tangara chrysotis*	0	0.899	0	0	0	0	0.025	0	0	0	0	0.006	0	0	0
Thraupidae	*Tangara cyanicollis*	0	0.966	0	0	0	0	0.034	0	0	0	0	0.009	0	0	0
Thraupidae	*Tangara cyanotis*	0	0.379	0	0	0	0	0.224	0	0	0	0	0.010	0	0	0
Thraupidae	*Tangara gyrola*	0	0.951	0	0	0	0	0.008	0	0	0	0	0.063	0	0	0
Thraupidae	*Tangara mexicana*	0	0.004	0	0	0	0	0.003	0	0	0	0	0.003	0	0	0
Thraupidae	*Tangara nigrocincta*	0	0.474	0	0	0	0	0.095	0	0	0	0	0.004	0	0	0
Thraupidae	*Tangara nigroviridis*	0.973	0.317	0.317	0.317	0.317	0	0.018	0	0	0	0	0.030	0	0	0
Thraupidae	*Tangara parzudakii*	0	0.254	0	0	0	0	0.034	0	0	0	0	0.003	0	0	0
Thraupidae	*Tangara punctata*	0	0.246	0	0	0	0	0.011	0	0	0	0	0.008	0	0	0
Thraupidae	*Tangara ruficervix*	0	0.579	0	0	0	0	0.451	0	0	0	0	0.005	0	0	0
Thraupidae	*Tangara schrankii*	0	0.002	0	0	0	0	0.002	0	0	0	0	0.002	0	0	0
Thraupidae	*Tangara vassorii*	0.550	0.940	0.940	0.940	0.940	0.972	0.021	0.021	0.021	0.021	0	0.325	0	0.325	0.325
Thraupidae	*Tangara velia*	0	0.003	0	0	0	0	0.003	0	0	0	0	0.003	0	0	0
Thraupidae	*Tangara viridicollis*	0.005	0.002	0.002	0.002	0.002	0	0.009	0	0	0	0	0.002	0	0	0
Thraupidae	*Tangara xanthocephala*	0.908	0.987	0.987	0.987	0.987	0	0.125	0	0	0	0	0.018	0	0	0
Thraupidae	*Tangara xanthogastra*	0	0.006	0	0	0	0	0.115	0	0	0	0	0.004	0	0	0
Thraupidae	*Tersina viridis*	0	0.258	0	0	0	0	0.048	0	0	0	0	0.005	0	0	0
Thraupidae	*Thlypopsis sordida*	0	0.034	0	0	0	0	0.884	0	0	0	0	0.002	0	0	0
Thraupidae	*Thraupis bonariensis*	0.002	0.068	0.068	0.068	0.068	0.003	0.066	0.066	0.066	0.066	0.002	0.002	0.002	0.002	0.002
Thraupidae	*Thraupis cyanocephala*	0.934	0.885	0.885	0.885	0.885	0.142	0.107	0.107	0.107	0.107	0	0.012	0	0	0
Thraupidae	*Thraupis episcopus*	0	0.671	0	0	0	0	0.007	0	0	0	0	0.008	0	0	0
Thraupidae	*Thraupis palmarum*	0	0.367	0	0	0	0	0.005	0	0	0	0	0.003	0	0	0
Thraupidae	*Trichothraupis melanops*	0	0.019	0	0	0	0	0.026	0	0	0	0	0.001	0	0	0
Tinamidae	*Crypturellus atrocapillus*	0	0.007	0	0	0	0	0.008	0	0	0	0	0.008	0	0	0
Tinamidae	*Crypturellus bartletti*	0	0.005	0	0	0	0	0.004	0	0	0	0	0.005	0	0	0
Tinamidae	*Crypturellus cinereus*	0	0.001	0	0	0	0	0.001	0	0	0	0	0.001	0	0	0

Tinamidae	*Crypturellus obsoletus*	0.326	0.012	0.012	0.012	0.012	0.021	0.899	0.899	0.899	0.899	0	0.003	0	0	0
Tinamidae	*Crypturellus soui*	0	0.447	0	0	0	0	0.051	0	0	0	0	0.003	0	0	0
Tinamidae	*Crypturellus strigulosus*	0	0.000	0	0	0	0	0.000	0	0	0	0	0.001	0	0	0
Tinamidae	*Crypturellus undulatus*	0	0.065	0	0	0	0	0.004	0	0	0	0	0.003	0	0	0
Tinamidae	*Crypturellus variegatus*	0	0.001	0	0	0	0	0.001	0	0	0	0	0.002	0	0	0
Tinamidae	*Nothocercus nigrocapillus*	0.043	0.256	0.256	0.256	0.256	0.049	0.484	0.484	0.484	0.484	0	0.016	0	0.016	0.016
Tinamidae	*Tinamus guttatus*	0	0.002	0	0	0	0	0.001	0	0	0	0	0.001	0	0	0
Tinamidae	*Tinamus major*	0	0.003	0	0	0	0	0.004	0	0	0	0	0.003	0	0	0
Tinamidae	*Tinamus tao*	0	0.002	0	0	0	0	0.002	0	0	0	0	0.002	0	0	0
Trogonidae	*Pharomachrus antisianus*	0	0.633	0	0.633	0.633	0	0.105	0	0	0	0	0.010	0	0	0
Trogonidae	*Pharomachrus auriceps*	0.418	0.723	0.723	0.723	0.723	0.006	0.033	0.033	0.033	0.033	0	0.014	0	0	0
Trogonidae	*Pharomachrus pavoninus*	0	0.017	0	0	0	0	0.122	0	0	0	0	0.173	0	0	0
Trogonidae	*Trogon collaris*	0	0.325	0	0	0	0	0.021	0	0	0	0	0.314	0	0	0
Trogonidae	*Trogon curucui*	0	0.003	0	0	0	0	0.006	0	0	0	0	0.003	0	0	0
Trogonidae	*Trogon melanurus*	0	0.002	0	0	0	0	0.005	0	0	0	0	0.002	0	0	0
Trogonidae	*Trogon personatus*	0.514	0.606	0.606	0.606	0.606	0.153	0.335	0.335	0.335	0.335	0.048	0.072	0.072	0.072	0.072
Trogonidae	*Trogon violaceus*	0	0.030	0	0	0	0	0.015	0	0	0	0	0.020	0	0	0
Trogonidae	*Trogon viridis*	0	0.006	0	0	0	0	0.005	0	0	0	0	0.004	0	0	0
Turdidae	*Catharus fuscater*	0.046	0.985	0.985	0.985	0.985	0	0.874	0	0.874	0.874	0	0.006	0	0	0
Turdidae	*Entomodestes leucotis*	0.009	0.066	0.066	0.066	0.066	0	0.022	0	0.022	0.022	0	0.006	0	0	0
Turdidae	*Myadestes ralloides*	0.788	0.996	0.996	0.996	0.996	0	0.319	0	0.319	0.319	0	0.002	0	0	0
Turdidae	*Turdus albicollis*	0	0.393	0	0	0	0	0.007	0	0	0	0	0.094	0	0	0
Turdidae	*Turdus chiguanco*	0.003	0.001	0.001	0.001	0.001	0.001	0.017	0.017	0.017	0.017	0.001	0.001	0.001	0.001	0.001
Turdidae	*Turdus fuscater*	0.917	0.366	0	0.366	0	0.014	0.013	0.013	0.013	0.013	0.629	0.753	0.753	0.753	0.753
Turdidae	*Turdus hauxwelli*	0	0.163	0	0	0	0	0.004	0	0	0	0	0.004	0	0	0
Turdidae	*Turdus ignobilis*	0	0.906	0	0	0	0	0.027	0	0	0	0	0.031	0	0	0
Turdidae	*Turdus leucops*	0.247	0.995	0.995	0.995	0.995	0	0.955	0	0	0	0	0.009	0	0	0
Turdidae	*Turdus nigriceps*	0	0.552	0	0	0	0	0.962	0	0	0	0	0.001	0	0	0
Turdidae	*Turdus serranus*	0.739	0.975	0.975	0.975	0.975	0.065	0.698	0.698	0.698	0.698	0	0.098	0	0.098	0.098
Tyrannidae	*Elaenia albiceps*	0.322	0.144	0.144	0.144	0.144	0.128	0.020	0.020	0.020	0.020	0	0.303	0	0.303	0.303
Tyrannidae	*Elaenia flavogaster*	0	0.865	0	0	0	0	0.019	0	0	0	0	0.004	0	0	0
Tyrannidae	*Elaenia gigas*	0	0.049	0	0	0	0	0.047	0	0	0	0	0.021	0	0	0
Tyrannidae	*Elaenia obscura*	0.254	0.347	0.347	0.347	0.347	0.567	0.916	0.916	0.916	0.916	0	0.069	0	0	0
Tyrannidae	*Elaenia pallatangae*	0.322	0.913	0.913	0.913	0.913	0.015	0.880	0.880	0.880	0.880	0	0.005	0	0.005	0.005
Tyrannidae	*Lophotriccus pileatus*	0	0.720	0	0	0	0	0.008	0	0	0	0	0.005	0	0	0
Tyrannidae	*Mionectes macconnelli*	0	0.002	0	0	0	0	0.002	0	0	0	0	0.003	0	0	0
Tyrannidae	*Mionectes oleagineus*	0	0.886	0	0	0	0	0.005	0	0	0	0	0.026	0	0	0
Tyrannidae	*Mionectes olivaceus*	0	0.164	0	0	0	0	0.893	0	0	0	0	0.004	0	0	0
Tyrannidae	*Mionectes striaticollis*	0.894	0.175	0.175	0.175	0.175	0.139	0.310	0.310	0.310	0.310	0	0.093	0	0.093	0.093
Tyrannidae	*Zimmerius bolivianus*	0.009	0.082	0.082	0.082	0.082	0	0.236	0	0.236	0.236	0	0.001	0	0	0
Tyrannidae	*Zimmerius gracilipes*	0	0.010	0	0	0	0	0.011	0	0	0	0	0.013	0	0	0

Table S4. Current and future community composition at all seven elevational levels, including probabilities of occurrence derived from species distribution models (SDMs) of all 232 frugivorous bird species of the Manú Biosphere Reserve (Peru). Based on the MIROC5 climate model, representative concentration pathway (RCP) 8.5. Species' elevational ranges were projected according to four potential dispersal scenarios: no range change, range contraction (lower range limit moves upwards), range expansion (upper range limit moves upwards), and range shift (both lower and upper range limit move upwards). SDMs were based on current and future local temperature and precipitation values.

		500 m					1000 m					1500 m					2000 m				
Family	Bird species	current	no range change	contraction	expansion	shift	current	no range change	contraction	expansion	shift	current	no range change	contraction	expansion	shift	current	no range change	contraction	expansion	shift
Cardinalidae	*Cyanocompsa cyanoides*	0.354	0.980	0	0.980	0	0.965	0.990	0.990	0.990	0.990	0	0.898	0	0.898	0.898	0	0.981	0	0	0
Cardinalidae	*Pheucticus aureoventris*	0	0.008	0	0	0	0.302	0.597	0	0.597	0	0.008	0.163	0.163	0.163	0.163	0.028	0.232	0.232	0.232	0.232
Cardinalidae	*Pheucticus chrysogaster*	0	0.003	0	0	0	0.063	0.215	0	0.215	0	0.476	0.005	0.005	0.005	0.005	0.520	0.036	0.036	0.036	0.036
Cardinalidae	*Saltator aurantiirostris*	0	0.002	0	0	0	0	0.002	0	0	0	0	0.002	0	0	0	0	0.003	0	0	0
Cardinalidae	*Saltator coerulescens*	0.023	0.072	0	0.072	0	0	0.566	0	0.566	0.566	0	0.938	0	0	0	0	0.137	0	0	0
Cardinalidae	*Saltator grossus*	0.195	0.886	0	0.886	0	0.988	0.976	0.976	0.976	0.976	0	0.963	0	0.963	0.963	0	0.621	0	0	0
Cardinalidae	*Saltator maximus*	0.514	0.729	0	0.729	0	0.903	0.962	0.962	0.962	0.962	0.136	0.955	0.955	0.955	0.955	0.941	0.756	0.756	0.756	0.756
Columbidae	*Geotrygon montana*	0.731	0.893	0	0.893	0	0.270	0.880	0.880	0.880	0.880	0	0.819	0	0.819	0.819	0	0.831	0	0.831	0.831
Columbidae	*Patagioenas cayennensis*	0.067	0.730	0	0.730	0	0	0.045	0	0.045	0.045	0	0.989	0	0	0	0	0.717	0	0	0
Columbidae	*Patagioenas fasciata*	0	0.007	0	0	0	0	0.003	0	0	0	0.790	0.167	0	0.167	0	0.993	0.326	0.326	0.326	0.326
Columbidae	*Patagioenas plumbea*	0.075	0.088	0	0.088	0	0.344	0.768	0.768	0.768	0.768	0.030	0.991	0.991	0.991	0.991	0	0.899	0	0.899	0.899
Columbidae	*Patagioenas subvinacea*	0.008	0.136	0	0.136	0	0.593	0.980	0.980	0.980	0.980	0.698	0.967	0.967	0.967	0.967	0	0.758	0	0.758	0.758
Corvidae	*Cyanocorax cyanomelas*	0.338	0.004	0	0.004	0	0.496	0.972	0.972	0.972	0.972	0	0.761	0	0.761	0.761	0	0.260	0	0	0
Corvidae	*Cyanocorax violaceus*	0.544	0.278	0	0.278	0	0.207	0.991	0.991	0.991	0.991	0	0.955	0	0.955	0.955	0	0.599	0	0	0
Corvidae	*Cyanocorax yncas*	0	0.008	0	0	0	0	0.007	0	0	0	0.453	0.035	0	0.035	0	0.994	0.776	0.776	0.776	0.776
Cotingidae	*Ampelioides tschudii*	0	0.080	0	0	0	0.765	0.790	0	0.790	0	0	0.954	0	0.954	0.954	0	0.854	0	0	0
Cotingidae	*Ampelion rubrocristatus*	0	0.005	0	0	0	0	0.014	0	0	0	0	0.176	0	0	0	0	0.085	0	0	0
Cotingidae	*Ampelion rufaxilla*	0	0.024	0	0	0	0	0.036	0	0	0	0	0.352	0	0	0	0.953	0.554	0	0.554	0
Cotingidae	*Cephalopterus ornatus*	0.371	0.659	0	0.659	0	0.044	0.935	0.935	0.935	0.935	0.534	0.681	0.681	0.681	0.681	0	0.681	0	0.681	0.681
Cotingidae	*Conioptilon mcilhennyi*	0	0.001	0	0.001	0	0	0.431	0	0.431	0.431	0	0.094	0	0	0	0	0.069	0	0	0
Cotingidae	*Cotinga cayana*	0.070	0.024	0	0.024	0	0	0.589	0	0.589	0.589	0	0.218	0	0	0	0	0.166	0	0	0
Cotingidae	*Cotinga maynana*	0.023	0.000	0	0	0	0.271	0.994	0.994	0.994	0.994	0	0.338	0	0.338	0.338	0	0.000	0	0	0
Cotingidae	*Gymnoderus foetidus*	0.010	0.230	0	0.230	0	0	0.963	0	0.963	0.963	0	0.002	0	0	0	0	0.002	0	0	0
Cotingidae	*Iodopleura isabellae*	0.842	0.375	0	0.375	0	0	0.990	0	0.990	0.990	0	0.498	0	0	0	0	0.005	0	0	0
Cotingidae	*Laniocera hypopyrra*	0.016	0.015	0	0.015	0	0	0.890	0	0.890	0.890	0	0.126	0	0	0	0	0.023	0	0	0
Cotingidae	*Lipaugus vociferans*	0.041	0.012	0	0.012	0	0	0.984	0	0.984	0.984	0	0.006	0	0	0	0	0.018	0	0	0
Cotingidae	*Oxyruncus cristatus*	0	0.059	0	0	0	0.729	0.171	0	0.171	0	0	0.267	0	0.267	0.267	0	0.641	0	0	0
Cotingidae	*Pipreola arcuata*	0	0.001	0	0	0	0	0.006	0	0	0	0	0.010	0	0	0	0.906	0.022	0	0.022	0
Cotingidae	*Pipreola frontalis*	0	0.025	0	0	0	0.926	0.712	0	0.712	0	0.913	0.906	0	0.906	0	0	0.916	0	0.916	0.916
Cotingidae	*Pipreola intermedia*	0	0.003	0	0	0	0	0.003	0	0	0	0.036	0.010	0	0.010	0	0.054	0.007	0.007	0.007	0.007
Cotingidae	*Porphyrolaema porphyrolaema*	0	0.022	0	0.022	0	0	0.664	0	0.664	0.664	0	0.231	0	0	0	0	0.098	0	0	0
Cotingidae	*Querula purpurata*	0.021	0.011	0	0.011	0	0.436	0.944	0.944	0.944	0.944	0	0.950	0	0.950	0.950	0	0.882	0	0	0
Cotingidae	*Rupicola peruvianus*	0	0.003	0	0	0	0.996	0.311	0	0.311	0	0.995	0.930	0.930	0.930	0.930	0.909	0.995	0.995	0.995	0.995
Cotingidae	*Snowornis subalaris*	0	0.010	0	0	0	0.976	0.970	0	0.970	0	0	0.945	0	0.945	0.945	0	0.921	0	0	0
Cotingidae	*Tityra cayana*	0	0.077	0	0.077	0	0	0.874	0	0.874	0.874	0	0.332	0	0	0	0	0.220	0	0	0
Cotingidae	*Tityra inquisitor*	0.856	0.462	0	0.462	0	0	0.706	0	0.706	0.706	0	0.966	0	0	0	0	0.986	0	0	0

Cotingidae	*Tityra semifasciata*	0.424	0.867	0	0.867	0	0.967	0.976	0.976	0.976	0.976	0.984	0.990	0.990	0.990	0.990	0	0.708	0	0.708	0.708
Cracidae	*Aburria aburri*	0	0.016	0	0	0	0.985	0.996	0	0.996	0	0.995	0.931	0.931	0.931	0.931	0	0.998	0	0.998	0.998
Cracidae	*Mitu tuberosum*	0.903	0.336	0	0.336	0	0.044	0.993	0.993	0.993	0.993	0	0.192	0	0.192	0.192	0	0.010	0	0	0
Cracidae	*Ortalis guttata*	0.577	0.020	0	0.020	0	0.978	0.649	0.649	0.649	0.649	0.136	0.990	0.990	0.990	0.990	0	0.974	0	0.974	0.974
Cracidae	*Penelope jacquacu*	0.078	0.022	0	0.022	0	0.139	0.988	0.988	0.988	0.988	0.004	0.821	0.821	0.821	0.821	0	0.189	0	0.189	0.189
Cracidae	*Penelope montagnii*	0	0.007	0	0	0	0.224	0.008	0	0.008	0	0.109	0.588	0.588	0.588	0.588	0.491	0.036	0.036	0.036	0.036
Cracidae	*Pipile cumanensis*	0.113	0.024	0	0.024	0	0	0.983	0	0.983	0.983	0	0.493	0	0	0	0	0.006	0	0	0
Icteridae	*Cacicus cela*	0.789	0.071	0	0.071	0	0.008	0.868	0.868	0.868	0.868	0	0.189	0	0.189	0.189	0	0.711	0	0	0
Icteridae	*Cacicus chrysonotus*	0	0.002	0	0	0	0	0.005	0	0	0	0	0.628	0	0	0	0	0.287	0	0	0
Icteridae	*Cacicus haemorrhous*	0.006	0.040	0	0.040	0	0.007	0.041	0.041	0.041	0.041	0	0.067	0	0.067	0.067	0	0.114	0	0	0
Icteridae	*Clypicterus oseryi*	0.988	0.026	0	0.026	0	0	0.996	0	0.996	0.996	0	0.192	0	0	0	0	0.066	0	0	0
Icteridae	*Icterus icterus*	0.074	0.484	0	0.484	0	0	0.080	0	0.080	0.080	0	0.040	0	0	0	0	0.049	0	0	0
Icteridae	*Molothrus oryzivorus*	0.216	0.059	0	0.059	0	0	0.778	0	0.778	0.778	0	0.258	0	0	0	0	0.905	0	0	0
Icteridae	*Psarocolius angustifrons*	0.244	0.005	0	0.005	0	0.493	0.981	0.981	0.981	0.981	0.073	0.943	0.943	0.943	0.943	0.707	0.954	0.954	0.954	0.954
Icteridae	*Psarocolius atrovirens*	0	0.002	0	0	0	0.699	0.012	0	0.012	0	0.775	0.546	0	0.546	0	0.551	0.982	0.982	0.982	0.982
Icteridae	*Psarocolius bifasciatus*	0.932	0.483	0	0.483	0	0	0.804	0	0.804	0.804	0	0.518	0	0.518	0.518	0	0.862	0	0	0
Icteridae	*Psarocolius decumanus*	0.892	0.262	0	0.262	0	0.978	0.786	0.786	0.786	0.786	0	0.408	0	0.408	0.408	0	0.933	0	0	0
Odontophoridae	*Odontophorus balliviani*	0	0.040	0	0	0	0.148	0.005	0	0.005	0	0.956	0.090	0.090	0.090	0.090	0.955	0.062	0.062	0.062	0.062
Odontophoridae	*Odontophorus speciosus*	0	0.007	0	0	0	0.981	0.031	0	0.031	0	0.828	0.981	0	0.981	0	0.418	0.988	0.988	0.988	0.988
Odontophoridae	*Odontophorus stellatus*	0.016	0.003	0	0.003	0	0.253	0.998	0.998	0.998	0.998	0	0.253	0	0.253	0.253	0	0.134	0	0	0
Picidae	*Celeus elegans*	0.109	0.058	0	0.058	0	0	0.981	0	0.981	0.981	0	0.003	0	0	0	0	0.003	0	0	0
Picidae	*Celeus flavus*	0.201	0.158	0	0.158	0	0	0.827	0	0.827	0.827	0	0.018	0	0	0	0	0.008	0	0	0
Picidae	*Celeus grammicus*	0.791	0.408	0	0.408	0	0.030	0.993	0.993	0.993	0.993	0	0.093	0	0.093	0.093	0	0.038	0	0	0
Picidae	*Celeus spectabilis*	0.201	0.000	0	0	0	0	1.000	0	1.000	1.000	0	0.315	0	0	0	0	0.257	0	0	0
Picidae	*Celeus torquatus*	0.190	0.226	0	0.226	0	0	0.334	0	0.334	0.334	0	0.012	0	0	0	0	0.011	0	0	0
Picidae	*Colaptes rubiginosus*	0	0.005	0	0	0	0.985	0.603	0	0.603	0	0.995	0.980	0.980	0.980	0.980	0.986	0.950	0.950	0.950	0.950
Pipridae	*Chiroxiphia boliviana*	0	0.007	0	0	0	0.142	0.135	0	0.135	0	0.123	0.905	0.905	0.905	0.905	0.028	0.881	0.881	0.881	0.881
Pipridae	*Chiroxiphia pareola*	0.463	0.150	0	0.150	0	0	0.923	0	0.923	0.923	0	0.314	0	0	0	0	0.041	0	0	0
Pipridae	*Lepidothrix coeruleocapilla*	0	0.007	0	0	0	0.972	0.556	0	0.556	0	0.044	0.984	0.984	0.984	0.984	0	0.951	0	0.951	0.951
Pipridae	*Lepidothrix coronata*	0.825	0.187	0	0.187	0	0	0.969	0	0.969	0.969	0	0.672	0	0	0	0	0.213	0	0	0
Pipridae	*Machaeropterus pyrocephalus*	0.074	0.004	0	0.004	0	0.240	0.986	0.986	0.986	0.986	0	0.064	0	0.064	0.064	0	0.121	0	0	0
Pipridae	*Manacus manacus*	0.895	0.951	0	0.951	0	0	0.958	0	0.958	0.958	0	0.994	0	0.994	0.994	0	0.442	0	0	0
Pipridae	*Neopelma sulphureiventer*	0.311	0.083	0	0.083	0	0	0.130	0	0.130	0.130	0	0.039	0	0	0	0	0.037	0	0	0
Pipridae	*Pipra chloromeros*	0.409	0.013	0	0.013	0	0.851	0.762	0.762	0.762	0.762	0	0.386	0	0.386	0.386	0	0.740	0	0	0
Pipridae	*Pipra fasciicauda*	0.005	0.003	0	0.003	0	0.413	0.985	0.985	0.985	0.985	0	0.679	0	0.679	0.679	0	0.318	0	0	0
Pipridae	*Xenopipo holochlora*	0.983	0.262	0	0.262	0	0.799	0.990	0.990	0.990	0.990	0	0.881	0	0.881	0.881	0	0.967	0	0	0
Pipridae	*Xenopipo unicolor*	0	0.002	0	0	0	0.500	0.056	0	0.056	0	0.916	0.810	0	0.810	0	0	0.875	0	0.875	0.875
Psittacidae	*Amazona farinosa*	0.033	0.573	0	0.573	0	0.034	0.987	0.987	0.987	0.987	0	0.971	0	0.971	0.971	0	0.055	0	0	0
Psittacidae	*Amazona mercenaria*	0	0.028	0	0	0	0.974	0.511	0	0.511	0	0.974	0.811	0.811	0.811	0.811	0.977	0.973	0.973	0.973	0.973
Psittacidae	*Amazona ochrocephala*	0.009	0.314	0	0.314	0	0	0.937	0	0.937	0.937	0	0.043	0	0	0	0	0.009	0	0	0
Psittacidae	*Ara ararauna*	0.506	0.070	0	0.070	0	0.010	0.939	0.939	0.939	0.939	0	0.012	0	0.012	0.012	0	0.027	0	0	0
Psittacidae	*Ara chloropterus*	0.079	0.194	0	0.194	0	0.004	0.847	0.847	0.847	0.847	0	0.016	0	0.016	0.016	0	0.006	0	0	0
Psittacidae	*Ara macao*	0.007	0.022	0	0.022	0	0.070	0.127	0.127	0.127	0.127	0	0.097	0	0.097	0.097	0	0.110	0	0	0
Psittacidae	*Ara militaris*	0	0.051	0	0	0	0.994	0.929	0	0.929	0	0.747	0.995	0.995	0.995	0.995	0	0.761	0	0.761	0.761
Psittacidae	*Ara severus*	0.114	0.945	0	0.945	0	0.120	0.990	0.990	0.990	0.990	0	0.991	0	0.991	0.991	0	0.061	0	0	0
Psittacidae	*Aratinga leucophthalma*	0.019	0.011	0	0.011	0	0.021	0.832	0.832	0.832	0.832	0.006	0.165	0.165	0.165	0.165	0	0.195	0	0.195	0.195
Psittacidae	*Aratinga mitrata*	0	0.001	0	0	0	0	0.001	0	0	0	0	0.016	0	0	0	0.001	0.003	0	0.003	0
Psittacidae	*Aratinga weddellii*	0.179	0.003	0	0.003	0	0	0.877	0	0.877	0.877	0	0.156	0	0	0	0	0.030	0	0	0
Psittacidae	*Bolborhynchus lineola*	0	0.022	0	0	0	0	0.061	0	0	0	0.766	0.179	0	0.179	0	0.800	0.414	0.414	0.414	0.414
Psittacidae	*Bolborhynchus orbygnesius*	0	0.011	0	0	0	0	0.011	0	0	0	0	0.049	0	0	0	0	0.049	0	0	0

Psittacidae	*Brotogeris cyanoptera*	0.052	0.147	0	0.147	0	0	0.983	0	0.983	0.983	0	0.004	0	0	0	0	0.085	0	0	0
Psittacidae	*Brotogeris sanctithomae*	0.041	0.086	0	0.086	0	0	0.287	0	0.287	0.287	0	0.059	0	0	0	0	0.055	0	0	0
Psittacidae	*Forpus modestus*	0.017	0.017	0	0.017	0	0	0.801	0	0.801	0.801	0	0.360	0	0	0	0	0.350	0	0	0
Psittacidae	*Nannopsittaca dachilleae*	0.263	0.992	0	0.992	0	0.009	0.249	0.249	0.249	0.249	0	0.019	0	0.019	0.019	0	0.002	0	0	0
Psittacidae	*Orthopsittaca manilata*	0.098	0.007	0	0.007	0	0	0.957	0	0.957	0.957	0	0.019	0	0	0	0	0.003	0	0	0
Psittacidae	*Pionites leucogaster*	0.004	0.000	0	0	0	0	0.241	0	0.241	0.241	0	0.000	0	0	0	0	0.000	0	0	0
Psittacidae	*Pionus menstruus*	0.137	0.542	0	0.542	0	0.489	0.981	0.981	0.981	0.981	0.078	0.762	0.762	0.762	0.762	0.653	0.050	0.050	0.050	0.050
Psittacidae	*Pionus tumultuosus*	0	0.040	0	0	0	0	0.009	0	0	0	0.959	0.418	0	0.418	0	0.989	0.024	0.024	0.024	0.024
Psittacidae	*Primolius couloni*	0.109	0.006	0	0.006	0	0.050	0.991	0.991	0.991	0.991	0	0.042	0	0.042	0.042	0	0.008	0	0	0
Psittacidae	*Pyrilia barrabandi*	0.006	0.006	0	0.006	0	0	0.030	0	0.030	0.030	0	0.642	0	0	0	0	0.006	0	0	0
Psittacidae	*Pyrrhura roseifrons*	0.005	0.005	0	0.005	0	0.005	0.941	0.941	0.941	0.941	0	0.104	0	0.104	0.104	0	0.005	0	0	0
Psittacidae	*Pyrrhura rupicola*	0.018	0.018	0	0.018	0	0.433	0.890	0.890	0.890	0.890	0	0.202	0	0.202	0.202	0	0.226	0	0	0
Psittacidae	*Touit huetii*	0.264	0.062	0	0.062	0	0.005	0.941	0.941	0.941	0.941	0	0.012	0	0.012	0.012	0	0.007	0	0	0
Psophiidae	*Psophia leucoptera*	0.995	0.159	0	0.159	0	0.057	0.996	0.996	0.996	0.996	0	0.313	0	0.313	0.313	0	0.146	0	0	0
Ramphastidae	*Andigena hypoglauca*	0	0.003	0	0	0	0	0.003	0	0	0	0.017	0.003	0	0.003	0	0.008	0.003	0.003	0.003	0.003
Ramphastidae	*Aulacorhynchus coeruleicinctis*	0	0.009	0	0	0	0	0.009	0	0	0	0.915	0.635	0	0.635	0	0.707	0.863	0.863	0.863	0.863
Ramphastidae	*Aulacorhynchus derbianus*	0	0.240	0	0	0	0.984	0.982	0	0.982	0	0.705	0.983	0.983	0.983	0.983	0	0.912	0	0.912	0.912
Ramphastidae	*Aulacorhynchus prasinus*	0.116	0.001	0	0.001	0	0.937	0.986	0.986	0.986	0.986	0.948	0.898	0.898	0.898	0.898	0	0.194	0	0.194	0.194
Ramphastidae	*Capito auratus*	0.079	0.012	0	0.012	0	0.018	0.880	0.880	0.880	0.880	0	0.568	0	0.568	0.568	0	0.003	0	0	0
Ramphastidae	*Eubucco richardsoni*	0.961	0.010	0	0.010	0	0.376	0.986	0.986	0.986	0.986	0	0.966	0	0.966	0.966	0	0.750	0	0	0
Ramphastidae	*Eubucco tucinkae*	0.602	0.073	0	0.073	0	0	0.998	0	0.998	0.998	0	0.112	0	0	0	0	0.086	0	0	0
Ramphastidae	*Eubucco versicolor*	0	0.246	0	0	0	0.909	0.003	0	0.003	0	0.063	0.924	0.924	0.924	0.924	0.004	0.570	0.570	0.570	0.570
Ramphastidae	*Pteroglossus azara*	0.887	0.093	0	0.093	0	0.034	0.971	0.971	0.971	0.971	0	0.340	0	0.340	0.340	0	0.009	0	0	0
Ramphastidae	*Pteroglossus beauharnaesii*	0.096	0.024	0	0.024	0	0	0.875	0	0.875	0.875	0	0.009	0	0.009	0.009	0	0.005	0	0	0
Ramphastidae	*Pteroglossus castanotis*	0.645	0.029	0	0.029	0	0.135	0.920	0.920	0.920	0.920	0	0.277	0	0.277	0.277	0	0.053	0	0	0
Ramphastidae	*Pteroglossus inscriptus*	0.384	0.023	0	0.023	0	0	0.901	0	0.901	0.901	0	0.046	0	0	0	0	0.024	0	0	0
Ramphastidae	*Ramphastos tucanus*	0.007	0.034	0	0.034	0	0	0.986	0	0.986	0.986	0	0.286	0	0	0	0	0.022	0	0	0
Ramphastidae	*Ramphastos vitellinus*	0.204	0.460	0	0.460	0	0	0.894	0	0.894	0.894	0	0.419	0	0	0	0	0.032	0	0	0
Ramphastidae	*Selenidera reinwardtii*	0.909	0.005	0	0.005	0	0.154	0.849	0.849	0.849	0.849	0.001	0.835	0.835	0.835	0.835	0	0.019	0	0.019	0.019
Steatornithidae	*Steatornis caripensis*	0.992	0.264	0	0.264	0	0	0.991	0	0.991	0.991	0	0.964	0	0	0	0	0.956	0	0	0
Thraupidae	*Anisognathus igniventris*	0	0.003	0	0	0	0	0.044	0	0	0	0	0.375	0	0	0	0	0.008	0	0	0
Thraupidae	*Anisognathus somptuosus*	0	0.004	0	0	0	0	0.057	0	0	0	0.684	0.045	0	0.045	0	0.998	0.543	0.543	0.543	0.543
Thraupidae	*Buthraupis montana*	0	0.006	0	0	0	0	0.083	0	0	0	0	0.263	0	0	0	0	0.005	0	0	0
Thraupidae	*Catamblyrhynchus diadema*	0	0.010	0	0	0	0	0.010	0	0	0	0	0.083	0	0	0	0.862	0.066	0	0.066	0
Thraupidae	*Chlorochrysa calliparaea*	0	0.007	0	0	0	0.876	0.967	0	0.967	0	0.585	0.819	0	0.819	0	0.988	0.614	0.614	0.614	0.614
Thraupidae	*Chlorophanes spiza*	0.924	0.319	0	0.319	0	0.960	0.966	0.966	0.966	0.966	0	0.964	0	0.964	0.964	0	0.988	0	0	0
Thraupidae	*Chlorophonia cyanea*	0.051	0.004	0	0.004	0	0.913	0.199	0.199	0.199	0.199	0.993	0.978	0.978	0.978	0.978	0.335	0.890	0.890	0.890	0.890
Thraupidae	*Chlorornis riefferii*	0	0.003	0	0	0	0	0.367	0	0	0	0	0.505	0	0	0	0	0.522	0	0	0
Thraupidae	*Chlorospingus canigularis*	0	0.006	0	0	0	0.970	0.006	0	0.006	0	0.881	0.610	0	0.610	0	0	0.826	0	0.826	0.826
Thraupidae	*Chlorospingus flavigularis*	0	0.010	0	0	0	0.985	0.666	0	0.666	0	0.995	0.996	0.996	0.996	0.996	0.985	0.994	0.994	0.994	0.994
Thraupidae	*Chlorospingus ophthalmicus*	0	0.002	0	0	0	0.974	0.212	0	0.212	0	0.129	0.122	0	0.122	0	0.166	0.206	0.206	0.206	0.206
Thraupidae	*Chlorospingus parvirostris*	0	0.007	0	0	0	0	0.056	0	0	0	0.966	0.859	0	0.859	0	0.951	0.778	0.778	0.778	0.778
Thraupidae	*Cissopis leverianus*	0.982	0.009	0	0.009	0	0.971	0.919	0.919	0.919	0.919	0.010	0.898	0.898	0.898	0.898	0	0.789	0	0.789	0.789
Thraupidae	*Conirostrum cinereum*	0	0.003	0	0	0	0	0.003	0	0	0	0	0.005	0	0	0	0	0.068	0	0	0
Thraupidae	*Conirostrum speciosum*	0.016	0.015	0	0.015	0	0	0.083	0	0.083	0.083	0	0.565	0	0	0	0	0.260	0	0	0
Thraupidae	*Cyanerpes caeruleus*	0.868	0.693	0	0.693	0	0.665	0.928	0.928	0.928	0.928	0	0.987	0	0.987	0.987	0	0.856	0	0	0
Thraupidae	*Cyanerpes cyaneus*	0.018	0.648	0	0.648	0	0.005	0.034	0.034	0.034	0.034	0	0.417	0	0.417	0.417	0	0.009	0	0	0
Thraupidae	*Dacnis cayana*	0.952	0.347	0	0.347	0	0.840	0.179	0.179	0.179	0.179	0.093	0.969	0.969	0.969	0.969	0	0.822	0	0.822	0.822
Thraupidae	*Dacnis flaviventer*	0.894	0.035	0	0.035	0	0.209	0.993	0.993	0.993	0.993	0	0.374	0	0.374	0.374	0	0.018	0	0	0
Thraupidae	*Dacnis lineata*	0.344	0.033	0	0.033	0	0.893	0.402	0.402	0.402	0.402	0	0.979	0	0.979	0.979	0	0.969	0	0	0

Thraupidae	*Delothraupis castaneoventris*	0	0.004	0	0	0	0	0.009	0	0	0	0	0.080	0	0	0	0	0.219	0	0	0
Thraupidae	*Diglossa brunneiventris*	0	0.001	0	0	0	0	0.001	0	0	0	0	0.001	0	0	0	0	0.001	0	0	0
Thraupidae	*Diglossa caerulescens*	0	0.005	0	0	0	0	0.004	0	0	0	0.041	0.132	0	0.132	0	0.021	0.622	0.622	0.622	0.622
Thraupidae	*Diglossa cyanea*	0	0.002	0	0	0	0	0.020	0	0	0	0.075	0.096	0	0.096	0	0.982	0.072	0.072	0.072	0.072
Thraupidae	*Diglossa glauca*	0	0.049	0	0	0	0.986	0.627	0	0.627	0	0.983	0.980	0	0.980	0	0.950	0.983	0.983	0.983	0.983
Thraupidae	*Diglossa mystacalis*	0	0.001	0	0	0	0	0.001	0	0	0	0	0.047	0	0	0	0	0.013	0	0	0
Thraupidae	*Diglossa sittoides*	0	0.002	0	0	0	0	0.005	0	0	0	0	0.491	0	0	0	0.967	0.700	0	0.700	0
Thraupidae	*Dubusia taeniata*	0	0.001	0	0	0	0	0.001	0	0	0	0	0.002	0	0	0	0.106	0.006	0	0.006	0
Thraupidae	*Euphonia chlorotica*	0	0.074	0	0.074	0	0	0.008	0	0.008	0.008	0	0.009	0	0	0	0	0.115	0	0	0
Thraupidae	*Euphonia chrysopasta*	0.882	0.074	0	0.074	0	0	0.961	0	0.961	0.961	0	0.867	0	0.867	0.867	0	0.393	0	0	0
Thraupidae	*Euphonia laniirostris*	0.020	0.188	0	0.188	0	0.794	0.863	0.863	0.863	0.863	0.909	0.824	0.824	0.824	0.824	0	0.782	0	0.782	0.782
Thraupidae	*Euphonia mesochrysa*	0	0.075	0	0	0	0.968	0.034	0	0.034	0	0.935	0.970	0.970	0.970	0.970	0	0.960	0	0.960	0.960
Thraupidae	*Euphonia minuta*	0.517	0.121	0	0.121	0	0.394	0.743	0.743	0.743	0.743	0	0.544	0	0.544	0.544	0	0.402	0	0	0
Thraupidae	*Euphonia rufiventris*	0.157	0.192	0	0.192	0	0	0.991	0	0.991	0.991	0	0.098	0	0.098	0.098	0	0.619	0	0	0
Thraupidae	*Euphonia xanthogaster*	0.950	0.305	0	0.305	0	0.988	0.272	0.272	0.272	0.272	0.655	0.989	0.989	0.989	0.989	0.914	0.958	0.958	0.958	0.958
Thraupidae	*Iridophanes pulcherrimus*	0	0.006	0	0	0	0	0.000	0	0	0	0.984	0.396	0	0.396	0	0	0.874	0	0.874	0.874
Thraupidae	*Iridosornis analis*	0	0.011	0	0	0	0.983	0.050	0	0.050	0	0.864	0.793	0	0.793	0	0.886	0.991	0.991	0.991	0.991
Thraupidae	*Iridosornis jelskii*	0	0.018	0	0	0	0	0.551	0	0	0	0	0.743	0	0	0	0	0.171	0	0	0
Thraupidae	*Lamprospiza melanoleuca*	0.004	0.004	0	0.004	0	0	0.637	0	0.637	0.637	0	0.004	0	0	0	0	0.004	0	0	0
Thraupidae	*Pipraeidea melanonota*	0.441	0.003	0	0.003	0	0.510	0.629	0.629	0.629	0.629	0.310	0.325	0.325	0.325	0.325	0.480	0.784	0.784	0.784	0.784
Thraupidae	*Piranga leucoptera*	0	0.060	0	0	0	0.963	0.013	0	0.013	0	0.860	0.992	0.992	0.992	0.992	0	0.771	0	0.771	0.771
Thraupidae	*Ramphocelus carbo*	0.188	0.132	0	0.132	0	0.366	0.636	0.636	0.636	0.636	0.103	0.398	0.398	0.398	0.398	0	0.907	0	0.907	0.907
Thraupidae	*Ramphocelus nigrogularis*	0.027	0.002	0	0.002	0	0.070	0.728	0.728	0.728	0.728	0	0.292	0	0.292	0.292	0	0.004	0	0	0
Thraupidae	*Schistochlamys melanopis*	0	0.901	0	0	0	0.931	0.838	0	0.838	0	0.239	0.993	0.993	0.993	0.993	0.044	0.330	0.330	0.330	0.330
Thraupidae	*Tachyphonus cristatus*	0	0.053	0	0.053	0	0	0.853	0	0.853	0.853	0	0.542	0	0	0	0	0.473	0	0	0
Thraupidae	*Tachyphonus rufiventer*	0.676	0.028	0	0.028	0	0.982	0.993	0.993	0.993	0.993	0	0.983	0	0.983	0.983	0	0.646	0	0	0
Thraupidae	*Tangara arthus*	0	0.003	0	0	0	0.985	0.388	0	0.388	0	0.636	0.972	0.972	0.972	0.972	0	0.962	0	0.962	0.962
Thraupidae	*Tangara callophrys*	0.830	0.011	0	0.011	0	0	0.978	0	0.978	0.978	0	0.680	0	0.680	0.680	0	0.053	0	0	0
Thraupidae	*Tangara chilensis*	0.278	0.005	0	0.005	0	0.976	0.898	0.898	0.898	0.898	0.243	0.847	0.847	0.847	0.847	0	0.945	0	0.945	0.945
Thraupidae	*Tangara chrysotis*	0	0.046	0	0	0	0.986	0.232	0	0.232	0	0.779	0.993	0.993	0.993	0.993	0.154	0.295	0.295	0.295	0.295
Thraupidae	*Tangara cyanicollis*	0.548	0.248	0	0.248	0	0.988	0.762	0	0.762	0	0.749	0.953	0.953	0.953	0.953	0.724	0.977	0.977	0.977	0.977
Thraupidae	*Tangara cyanotis*	0	0.010	0	0	0	0	0.283	0	0	0	0.932	0.738	0	0.738	0	0.948	0.903	0.903	0.903	0.903
Thraupidae	*Tangara gyrola*	0.419	0.020	0	0.020	0	0.992	0.236	0.236	0.236	0.236	0.898	0.972	0.972	0.972	0.972	0	0.994	0	0.994	0.994
Thraupidae	*Tangara mexicana*	0.389	0.027	0	0.027	0	0.243	0.991	0.991	0.991	0.991	0	0.263	0	0.263	0.263	0	0.012	0	0	0
Thraupidae	*Tangara nigrocincta*	0.978	0.069	0	0.069	0	0.564	0.998	0.998	0.998	0.998	0	0.784	0	0.784	0.784	0	0.753	0	0	0
Thraupidae	*Tangara nigroviridis*	0	0.004	0	0	0	0	0.009	0	0	0	0.962	0.186	0	0.186	0	0.992	0.885	0.885	0.885	0.885
Thraupidae	*Tangara parzudakii*	0	0.003	0	0	0	0	0.007	0	0	0	0.995	0.139	0	0.139	0	0.991	0.318	0.318	0.318	0.318
Thraupidae	*Tangara punctata*	0	0.033	0	0	0	0.989	0.952	0	0.952	0	0.427	0.869	0.869	0.869	0.869	0.277	0.855	0.855	0.855	0.855
Thraupidae	*Tangara ruficervix*	0	0.005	0	0	0	0.978	0.013	0	0.013	0	0.987	0.421	0	0.421	0	0.991	0.197	0.197	0.197	0.197
Thraupidae	*Tangara schrankii*	0.203	0.003	0	0.003	0	0.231	0.969	0.969	0.969	0.969	0	0.962	0	0.962	0.962	0	0.679	0	0	0
Thraupidae	*Tangara vassorii*	0	0.008	0	0	0	0	0.012	0	0	0	0	0.182	0	0	0	0.545	0.263	0	0.263	0
Thraupidae	*Tangara velia*	0.926	0.126	0	0.126	0	0	0.993	0	0.993	0.993	0	0.094	0	0	0	0	0.006	0	0	0
Thraupidae	*Tangara viridicollis*	0	0.014	0	0	0	0.066	0.002	0	0.002	0	0.023	0.004	0.004	0.004	0.004	0.133	0.297	0.297	0.297	0.297
Thraupidae	*Tangara xanthocephala*	0	0.002	0	0	0	0.871	0.003	0	0.003	0	0.459	0.285	0	0.285	0	0.974	0.790	0.790	0.790	0.790
Thraupidae	*Tangara xanthogastra*	0.583	0.045	0	0.045	0	0.926	0.992	0.992	0.992	0.992	0	0.971	0	0.971	0.971	0	0.322	0	0	0
Thraupidae	*Tersina viridis*	0.590	0.285	0	0.285	0	0.994	0.570	0.570	0.570	0.570	0	0.887	0	0.887	0.887	0	0.866	0	0.866	0.866
Thraupidae	*Thlypopsis sordida*	0.741	0.057	0	0.057	0	0	0.306	0	0.306	0.306	0	0.350	0	0	0	0	0.350	0	0	0
Thraupidae	*Thraupis bonariensis*	0	0.002	0	0	0	0.002	0.002	0	0.002	0	0.006	0.011	0	0.011	0	0.183	0.135	0.135	0.135	0.135
Thraupidae	*Thraupis cyanocephala*	0	0.002	0	0	0	0	0.007	0	0	0	0.794	0.005	0	0.005	0	0.938	0.149	0.149	0.149	0.149
Thraupidae	*Thraupis episcopus*	0.109	0.617	0	0.617	0	0.267	0.190	0.190	0.190	0.190	0.458	0.762	0.762	0.762	0.762	0	0.370	0	0.370	0.370

Thraupidae	*Thraupis palmarum*	0.092	0.076	0	0.076	0	0.307	0.152	0.152	0.152	0.152	0.321	0.978	0.978	0.978	0.978	0	0.407	0	0.407	0.407
Thraupidae	*Trichothraupis melanops*	0	0.003	0	0	0	0.010	0.004	0	0.004	0	0.010	0.336	0	0.336	0	0	0.751	0	0.751	0.751
Tinamidae	*Crypturellus atrocapillus*	0.449	0.012	0	0.012	0	0.963	0.959	0.959	0.959	0.959	0	0.984	0	0.984	0.984	0	0.891	0	0	0
Tinamidae	*Crypturellus bartletti*	0.007	0.010	0	0.010	0	0	0.037	0	0.037	0.037	0	0.010	0	0	0	0	0.005	0	0	0
Tinamidae	*Crypturellus cinereus*	0.006	0.059	0	0.059	0	0.001	0.972	0.972	0.972	0.972	0	0.500	0	0.500	0.500	0	0.007	0	0	0
Tinamidae	*Crypturellus obsoletus*	0.021	0.002	0	0.002	0	0.861	0.790	0.790	0.790	0.790	0.006	0.983	0.983	0.983	0.983	0.010	0.733	0.733	0.733	0.733
Tinamidae	*Crypturellus soui*	0.273	0.866	0	0.866	0	0.893	0.974	0.974	0.974	0.974	0.518	0.950	0.950	0.950	0.950	0	0.818	0	0.818	0.818
Tinamidae	*Crypturellus strigulosus*	0.081	0.004	0	0.004	0	0	0.705	0	0.705	0.705	0	0.003	0	0	0	0	0.001	0	0	0
Tinamidae	*Crypturellus undulatus*	0.015	0.004	0	0.004	0	0	0.681	0	0.681	0.681	0	0.024	0	0	0	0	0.003	0	0	0
Tinamidae	*Crypturellus variegatus*	0.017	0.025	0	0.025	0	0	0.627	0	0.627	0.627	0	0.007	0	0	0	0	0.002	0	0	0
Tinamidae	*Nothocercus nigrocapillus*	0	0.003	0	0	0	0	0.003	0	0	0	0	0.003	0	0	0	0.706	0.003	0	0.003	0
Tinamidae	*Tinamus guttatus*	0.092	0.005	0	0.005	0	0.002	0.996	0.996	0.996	0.996	0	0.031	0	0.031	0.031	0	0.024	0	0	0
Tinamidae	*Tinamus major*	0.668	0.572	0	0.572	0	0	0.305	0	0.305	0.305	0	0.391	0	0	0	0	0.037	0	0	0
Tinamidae	*Tinamus tao*	0.905	0.002	0	0.002	0	0.875	0.983	0.983	0.983	0.983	0	0.987	0	0.987	0.987	0	0.948	0	0	0
Trogonidae	*Pharomachrus antisianus*	0	0.050	0	0	0	0.993	0.022	0	0.022	0	0.803	0.513	0	0.513	0	0.936	0.934	0.934	0.934	0.934
Trogonidae	*Pharomachrus auriceps*	0	0.002	0	0	0	0	0.002	0	0	0	0.931	0.147	0	0.147	0	0.989	0.647	0.647	0.647	0.647
Trogonidae	*Pharomachrus pavoninus*	0.918	0.395	0	0.395	0	0.132	0.998	0.998	0.998	0.998	0	0.225	0	0.225	0.225	0	0.016	0	0	0
Trogonidae	*Trogon collaris*	0.004	0.138	0	0.138	0	0.965	0.616	0.616	0.616	0.616	0	0.993	0	0.993	0.993	0	0.982	0	0.982	0.982
Trogonidae	*Trogon curucui*	0.058	0.003	0	0.003	0	0.181	0.983	0.983	0.983	0.983	0.004	0.379	0.379	0.379	0.379	0	0.122	0	0.122	0.122
Trogonidae	*Trogon melanurus*	0.007	0.052	0	0.052	0	0.004	0.984	0.984	0.984	0.984	0	0.004	0	0.004	0.004	0	0.004	0	0	0
Trogonidae	*Trogon personatus*	0	0.011	0	0	0	0	0.221	0	0	0	0.712	0.166	0	0.166	0	0.908	0.433	0.433	0.433	0.433
Trogonidae	*Trogon violaceus*	0.694	0.945	0	0.945	0	0	0.717	0	0.717	0.717	0	0.727	0	0	0	0	0.250	0	0	0
Trogonidae	*Trogon viridis*	0.243	0.106	0	0.106	0	0	0.957	0	0.957	0.957	0	0.961	0	0	0	0	0.256	0	0	0
Turdidae	*Catharus fuscater*	0	0.008	0	0	0	0	0.005	0	0	0	0.978	0.095	0	0.095	0	0.549	0.181	0	0.181	0
Turdidae	*Entomodestes leucotis*	0	0.012	0	0	0	0	0.039	0	0	0	0.473	0.645	0	0.645	0	0.444	0.463	0.463	0.463	0.463
Turdidae	*Myadestes ralloides*	0	0.002	0	0	0	0.990	0.004	0	0.004	0	0.940	0.922	0.922	0.922	0.922	0.961	0.928	0.928	0.928	0.928
Turdidae	*Turdus albicollis*	0.068	0.016	0	0.016	0	0	0.921	0	0.921	0.921	0	0.948	0	0	0	0	0.049	0	0	0
Turdidae	*Turdus chiguanco*	0	0.001	0	0	0	0	0.001	0	0	0	0.001	0.002	0	0.002	0	0.001	0.004	0.004	0.004	0.004
Turdidae	*Turdus fuscater*	0	0.004	0	0	0	0	0.051	0	0	0	0	0.185	0	0	0	0	0.065	0	0	0
Turdidae	*Turdus hauxwelli*	0.037	0.038	0	0.038	0	0.125	0.603	0.603	0.603	0.603	0	0.069	0	0.069	0.069	0	0.057	0	0.057	0.057
Turdidae	*Turdus ignobilis*	0.332	0.562	0	0.562	0	0.985	0.475	0.475	0.475	0.475	0.052	0.362	0.362	0.362	0.362	0	0.920	0	0.920	0.920
Turdidae	*Turdus leucops*	0	0.037	0	0	0	0.919	0.360	0	0.360	0	0.753	0.640	0.640	0.640	0.640	0.902	0.632	0.632	0.632	0.632
Turdidae	*Turdus nigriceps*	0.592	0.002	0	0.002	0	0.873	0.700	0.700	0.700	0.700	0.116	0.969	0.969	0.969	0.969	0	0.652	0	0.652	0.652
Turdidae	*Turdus serranus*	0	0.005	0	0	0	0	0.070	0	0	0	0.173	0.242	0	0.242	0	0.986	0.303	0.303	0.303	0.303
Tyrannidae	*Elaenia albiceps*	0.042	0.003	0	0.003	0	0.005	0.041	0	0.041	0	0.004	0.007	0.007	0.007	0.007	0.722	0.078	0.078	0.078	0.078
Tyrannidae	*Elaenia flavogaster*	0.010	0.483	0	0.483	0	0	0.234	0	0.234	0.234	0	0.988	0	0	0	0	0.805	0	0	0
Tyrannidae	*Elaenia gigas*	0.992	0.300	0	0.300	0	0.991	0.993	0.993	0.993	0.993	0.364	0.989	0.989	0.989	0.989	0	0.936	0	0.936	0.936
Tyrannidae	*Elaenia obscura*	0	0.017	0	0	0	0	0.017	0	0	0	0	0.039	0	0	0	0.298	0.067	0	0.067	0
Tyrannidae	*Elaenia pallatangae*	0	0.041	0	0	0	0	0.005	0	0	0	0.951	0.358	0	0.358	0	0.994	0.534	0.534	0.534	0.534
Tyrannidae	*Lophotriccus pileatus*	0	0.006	0	0	0	0.948	0.289	0	0.289	0	0.988	0.978	0.978	0.978	0.978	0	0.934	0	0.934	0.934
Tyrannidae	*Mionectes macconnelli*	0.003	0.013	0	0.013	0	0.499	0.992	0.992	0.992	0.992	0	0.938	0	0.938	0.938	0	0.228	0	0	0
Tyrannidae	*Mionectes oleagineus*	0.335	0.770	0	0.770	0	0.958	0.994	0.994	0.994	0.994	0	0.970	0	0.970	0.970	0	0.598	0	0	0
Tyrannidae	*Mionectes olivaceus*	0.547	0.041	0	0.041	0	0.996	0.859	0.859	0.859	0.859	0.743	0.997	0.997	0.997	0.997	0	0.893	0	0.893	0.893
Tyrannidae	*Mionectes striaticollis*	0	0.004	0	0	0	0.325	0.520	0	0.520	0	0.263	0.337	0.337	0.337	0.337	0.994	0.919	0.919	0.919	0.919
Tyrannidae	*Zimmerius bolivianus*	0	0.003	0	0	0	0.956	0.005	0	0.005	0	0.737	0.972	0	0.972	0	0.385	0.733	0.733	0.733	0.733
Tyrannidae	*Zimmerius gracilipes*	0.036	0.087	0	0.087	0	0	0.969	0	0.969	0.969	0	0.222	0	0	0	0	0.059	0	0	0

Table S4. *Continued.*

		2500 m					3000 m					3500 m				
Family	Bird species	current	no range change	contraction	expansion	shift	current	no range change	contraction	expansion	shift	current	no range change	contraction	expansion	shift
Cardinalidae	*Cyanocompsa cyanoides*	0	0.577	0	0	0	0	0.006	0	0	0	0	0.003	0	0	0
Cardinalidae	*Pheucticus aureoventris*	0.007	0.008	0.008	0.008	0.008	0.043	0.063	0.063	0.063	0.063	0	0.004	0	0.004	0.004
Cardinalidae	*Pheucticus chrysogaster*	0.004	0.244	0.244	0.244	0.244	0.004	0.605	0.605	0.605	0.605	0.010	0.004	0.004	0.004	0.004
Cardinalidae	*Saltator aurantiirostris*	0	0.002	0	0	0	0.002	0.002	0	0.002	0	0.003	0.004	0.004	0.004	0.004
Cardinalidae	*Saltator coerulescens*	0	0.166	0	0	0	0	0.004	0	0	0	0	0.004	0	0	0
Cardinalidae	*Saltator grossus*	0	0.016	0	0	0	0	0.065	0	0	0	0	0.003	0	0	0
Cardinalidae	*Saltator maximus*	0.007	0.705	0.705	0.705	0.705	0	0.039	0	0.039	0.039	0	0.007	0	0	0
Columbidae	*Geotrygon montana*	0	0.267	0	0	0	0	0.002	0	0	0	0	0.005	0	0	0
Columbidae	*Patagioenas cayennensis*	0	0.737	0	0	0	0	0.100	0	0	0	0	0.004	0	0	0
Columbidae	*Patagioenas fasciata*	0.969	0.899	0.899	0.899	0.899	0.090	0.457	0.457	0.457	0.457	0.005	0.004	0.004	0.004	0.004
Columbidae	*Patagioenas plumbea*	0	0.838	0	0.838	0.838	0	0.408	0	0	0	0	0.031	0	0	0
Columbidae	*Patagioenas subvinacea*	0	0.964	0	0	0	0	0.100	0	0	0	0	0.007	0	0	0
Corvidae	*Cyanocorax cyanomelas*	0	0.003	0	0	0	0	0.003	0	0	0	0	0.003	0	0	0
Corvidae	*Cyanocorax violaceus*	0	0.067	0	0	0	0	0.003	0	0	0	0	0.003	0	0	0
Corvidae	*Cyanocorax yncas*	0	0.996	0	0.996	0.996	0	0.552	0	0	0	0	0.004	0	0	0
Cotingidae	*Ampelioides tschudii*	0	0.457	0	0	0	0	0.444	0	0	0	0	0.013	0	0	0
Cotingidae	*Ampelion rubrocristatus*	0.903	0.255	0	0.255	0	0.015	0.453	0	0.453	0	0	0.119	0	0.119	0.119
Cotingidae	*Ampelion rufaxilla*	0.856	0.933	0.933	0.933	0.933	0	0.912	0	0.912	0.912	0	0.082	0	0	0
Cotingidae	*Cephalopterus ornatus*	0	0.215	0	0	0	0	0.004	0	0	0	0	0.005	0	0	0
Cotingidae	*Conioptilon mcilhennyi*	0	0.077	0	0	0	0	0.063	0	0	0	0	0.088	0	0	0
Cotingidae	*Cotinga cayana*	0	0.037	0	0	0	0	0.005	0	0	0	0	0.008	0	0	0
Cotingidae	*Cotinga maynana*	0	0.000	0	0	0	0	0.000	0	0	0	0	0.000	0	0	0
Cotingidae	*Gymnoderus foetidus*	0	0.002	0	0	0	0	0.002	0	0	0	0	0.002	0	0	0
Cotingidae	*Iodopleura isabellae*	0	0.002	0	0	0	0	0.002	0	0	0	0	0.001	0	0	0
Cotingidae	*Laniocera hypopyrra*	0	0.015	0	0	0	0	0.015	0	0	0	0	0.015	0	0	0
Cotingidae	*Lipaugus vociferans*	0	0.002	0	0	0	0	0.002	0	0	0	0	0.003	0	0	0
Cotingidae	*Oxyruncus cristatus*	0	0.754	0	0	0	0	0.886	0	0	0	0	0.019	0	0	0
Cotingidae	*Pipreola arcuata*	0.612	0.999	0.999	0.999	0.999	0.356	0.965	0.965	0.965	0.965	0.189	0.193	0.193	0.193	0.193
Cotingidae	*Pipreola frontalis*	0	0.770	0	0	0	0	0.255	0	0	0	0	0.023	0	0	0
Cotingidae	*Pipreola intermedia*	0.095	0.051	0.051	0.051	0.051	0.302	0.988	0.988	0.988	0.988	0	0.124	0	0.124	0.124
Cotingidae	*Porphyrolaema porphyrolaema*	0	0.002	0	0	0	0	0.000	0	0	0	0	0.000	0	0	0
Cotingidae	*Querula purpurata*	0	0.004	0	0	0	0	0.003	0	0	0	0	0.001	0	0	0
Cotingidae	*Rupicola peruvianus*	0.733	0.998	0.998	0.998	0.998	0	0.087	0	0.087	0.087	0	0.003	0	0	0
Cotingidae	*Snowornis subalaris*	0	0.864	0	0	0	0	0.010	0	0	0	0	0.010	0	0	0
Cotingidae	*Tityra cayana*	0	0.033	0	0	0	0	0.011	0	0	0	0	0.002	0	0	0
Cotingidae	*Tityra inquisitor*	0	0.101	0	0	0	0	0.114	0	0	0	0	0.003	0	0	0
Cotingidae	*Tityra semifasciata*	0	0.835	0	0	0	0	0.066	0	0	0	0	0.007	0	0	0
Cracidae	*Aburria aburri*	0	0.979	0	0	0	0	0.125	0	0	0	0	0.009	0	0	0
Cracidae	*Mitu tuberosum*	0	0.001	0	0	0	0	0.001	0	0	0	0	0.001	0	0	0
Cracidae	*Ortalis guttata*	0	0.772	0	0	0	0	0.006	0	0	0	0	0.003	0	0	0
Cracidae	*Penelope jacquacu*	0	0.003	0	0	0	0	0.004	0	0	0	0	0.003	0	0	0

Cracidae	*Penelope montagnii*	0.624	0.982	0.982	0.982	0.982	0.151	0.906	0.906	0.906	0.906	0	0.007	0	0.007	0.007
Cracidae	*Pipile cumanensis*	0	0.005	0	0	0	0	0.005	0	0	0	0	0.006	0	0	0
Icteridae	*Cacicus cela*	0	0.178	0	0	0	0	0.006	0	0	0	0	0.005	0	0	0
Icteridae	*Cacicus chrysonotus*	0.874	0.996	0	0.996	0	0.295	0.084	0.084	0.084	0.084	0	0.004	0	0.004	0.004
Icteridae	*Cacicus haemorrhous*	0	0.016	0	0	0	0	0.035	0	0	0	0	0.003	0	0	0
Icteridae	*Clypicterus oseryi*	0	0.148	0	0	0	0	0.002	0	0	0	0	0.005	0	0	0
Icteridae	*Icterus icterus*	0	0.031	0	0	0	0	0.025	0	0	0	0	0.024	0	0	0
Icteridae	*Molothrus oryzivorus*	0	0.607	0	0	0	0	0.144	0	0	0	0	0.008	0	0	0
Icteridae	*Psarocolius angustifrons*	0	0.920	0	0.920	0.920	0	0.015	0	0	0	0	0.007	0	0	0
Icteridae	*Psarocolius atrovirens*	0.009	0.222	0.222	0.222	0.222	0	0.099	0	0.099	0.099	0	0.001	0	0	0
Icteridae	*Psarocolius bifasciatus*	0	0.006	0	0	0	0	0.002	0	0	0	0	0.002	0	0	0
Icteridae	*Psarocolius decumanus*	0	0.050	0	0	0	0	0.006	0	0	0	0	0.004	0	0	0
Odontophoridae	*Odontophorus balliviani*	0.475	0.406	0.406	0.406	0.406	0.411	0.658	0.658	0.658	0.658	0	0.014	0	0.014	0.014
Odontophoridae	*Odontophorus speciosus*	0	0.537	0	0.537	0.537	0	0.074	0	0	0	0	0.009	0	0	0
Odontophoridae	*Odontophorus stellatus*	0	0.039	0	0	0	0	0.001	0	0	0	0	0.001	0	0	0
Picidae	*Celeus elegans*	0	0.001	0	0	0	0	0.001	0	0	0	0	0.001	0	0	0
Picidae	*Celeus flavus*	0	0.008	0	0	0	0	0.009	0	0	0	0	0.008	0	0	0
Picidae	*Celeus grammicus*	0	0.007	0	0	0	0	0.006	0	0	0	0	0.014	0	0	0
Picidae	*Celeus spectabilis*	0	0.211	0	0	0	0	0.000	0	0	0	0	0.001	0	0	0
Picidae	*Celeus torquatus*	0	0.012	0	0	0	0	0.009	0	0	0	0	0.011	0	0	0
Picidae	*Colaptes rubiginosus*	0	0.973	0	0.973	0.973	0	0.365	0	0	0	0	0.005	0	0	0
Pipridae	*Chiroxiphia boliviana*	0	0.005	0	0.005	0.005	0	0.153	0	0	0	0	0.005	0	0	0
Pipridae	*Chiroxiphia pareola*	0	0.029	0	0	0	0	0.032	0	0	0	0	0.027	0	0	0
Pipridae	*Lepidothrix coeruleocapilla*	0	0.004	0	0	0	0	0.004	0	0	0	0	0.003	0	0	0
Pipridae	*Lepidothrix coronata*	0	0.013	0	0	0	0	0.011	0	0	0	0	0.012	0	0	0
Pipridae	*Machaeropterus pyrocephalus*	0	0.004	0	0	0	0	0.004	0	0	0	0	0.004	0	0	0
Pipridae	*Manacus manacus*	0	0.905	0	0	0	0	0.013	0	0	0	0	0.001	0	0	0
Pipridae	*Neopelma sulphureiventer*	0	0.031	0	0	0	0	0.042	0	0	0	0	0.056	0	0	0
Pipridae	*Pipra chloromeros*	0	0.007	0	0	0	0	0.006	0	0	0	0	0.006	0	0	0
Pipridae	*Pipra fasciicauda*	0	0.003	0	0	0	0	0.003	0	0	0	0	0.003	0	0	0
Pipridae	*Xenopipo holochlora*	0	0.022	0	0	0	0	0.379	0	0	0	0	0.001	0	0	0
Pipridae	*Xenopipo unicolor*	0	0.247	0	0	0	0	0.075	0	0	0	0	0.003	0	0	0
Psittacidae	*Amazona farinosa*	0	0.366	0	0	0	0	0.009	0	0	0	0	0.010	0	0	0
Psittacidae	*Amazona mercenaria*	0.981	0.980	0.980	0.980	0.980	0.948	0.809	0.809	0.809	0.809	0	0.843	0	0.843	0.843
Psittacidae	*Amazona ochrocephala*	0	0.005	0	0	0	0	0.005	0	0	0	0	0.008	0	0	0
Psittacidae	*Ara ararauna*	0	0.005	0	0	0	0	0.004	0	0	0	0	0.005	0	0	0
Psittacidae	*Ara chloropterus*	0	0.003	0	0	0	0	0.004	0	0	0	0	0.003	0	0	0
Psittacidae	*Ara macao*	0	0.003	0	0	0	0	0.003	0	0	0	0	0.003	0	0	0
Psittacidae	*Ara militaris*	0	0.619	0	0	0	0	0.006	0	0	0	0	0.001	0	0	0
Psittacidae	*Ara severus*	0	0.020	0	0	0	0	0.005	0	0	0	0	0.010	0	0	0
Psittacidae	*Aratinga leucophthalma*	0	0.013	0	0	0	0	0.133	0	0	0	0	0.004	0	0	0
Psittacidae	*Aratinga mitrata*	0.002	0.001	0.001	0.001	0.001	0.002	0.005	0.005	0.005	0.005	0	0.001	0	0.001	0.001
Psittacidae	*Aratinga weddellii*	0	0.002	0	0	0	0	0.002	0	0	0	0	0.002	0	0	0
Psittacidae	*Bolborhynchus lineola*	0.715	0.803	0.803	0.803	0.803	0.195	0.564	0.564	0.564	0.564	0	0.167	0	0.167	0.167
Psittacidae	*Bolborhynchus orbygnesius*	0.049	0.112	0	0.112	0	0.029	0.058	0	0.058	0	0.012	0.016	0.016	0.016	0.016
Psittacidae	*Brotogeris cyanoptera*	0	0.002	0	0	0	0	0.002	0	0	0	0	0.002	0	0	0
Psittacidae	*Brotogeris sanctithomae*	0	0.031	0	0	0	0	0.002	0	0	0	0	0.028	0	0	0
Psittacidae	*Forpus modestus*	0	0.017	0	0	0	0	0.017	0	0	0	0	0.017	0	0	0
Psittacidae	*Nannopsittaca dachilleae*	0	0.001	0	0	0	0	0.000	0	0	0	0	0.000	0	0	0
Psittacidae	*Orthopsittaca manilata*	0	0.003	0	0	0	0	0.003	0	0	0	0	0.010	0	0	0

Psittacidae	*Pionites leucogaster*	0	0.000	0	0	0	0	0.000	0	0	0	0	0.000	0	0	0
Psittacidae	*Pionus menstruus*	0	0.867	0	0.867	0.867	0	0.004	0	0	0	0	0.004	0	0	0
Psittacidae	*Pionus tumultuosus*	0.893	0.993	0.993	0.993	0.993	0.297	0.991	0.991	0.991	0.991	0	0.212	0	0.212	0.212
Psittacidae	*Primolius couloni*	0	0.005	0	0	0	0	0.012	0	0	0	0	0.006	0	0	0
Psittacidae	*Pyrilia barrabandi*	0	0.006	0	0	0	0	0.006	0	0	0	0	0.006	0	0	0
Psittacidae	*Pyrrhura roseifrons*	0	0.005	0	0	0	0	0.005	0	0	0	0	0.005	0	0	0
Psittacidae	*Pyrrhura rupicola*	0	0.572	0	0	0	0	0.018	0	0	0	0	0.018	0	0	0
Psittacidae	*Touit huetii*	0	0.001	0	0	0	0	0.000	0	0	0	0	0.000	0	0	0
Psophiidae	*Psophia leucoptera*	0	0.021	0	0	0	0	0.002	0	0	0	0	0.012	0	0	0
Ramphastidae	*Andigena hypoglauca*	0.230	0.026	0.026	0.026	0.026	0.767	0.688	0.688	0.688	0.688	0.814	0.147	0.147	0.147	0.147
Ramphastidae	*Aulacorhynchus coeruleicinctis*	0.043	0.520	0.520	0.520	0.520	0	0.900	0	0.900	0.900	0	0.010	0	0	0
Ramphastidae	*Aulacorhynchus derbianus*	0	0.451	0	0	0	0	0.657	0	0	0	0	0.019	0	0	0
Ramphastidae	*Aulacorhynchus prasinus*	0	0.956	0	0	0	0	0.419	0	0	0	0	0.240	0	0	0
Ramphastidae	*Capito auratus*	0	0.008	0	0	0	0	0.001	0	0	0	0	0.002	0	0	0
Ramphastidae	*Eubucco richardsoni*	0	0.002	0	0	0	0	0.003	0	0	0	0	0.002	0	0	0
Ramphastidae	*Eubucco tucinkae*	0	0.098	0	0	0	0	0.004	0	0	0	0	0.005	0	0	0
Ramphastidae	*Eubucco versicolor*	0	0.010	0	0.010	0.010	0	0.628	0	0	0	0	0.002	0	0	0
Ramphastidae	*Pteroglossus azara*	0	0.002	0	0	0	0	0.001	0	0	0	0	0.002	0	0	0
Ramphastidae	*Pteroglossus beauharnaesii*	0	0.004	0	0	0	0	0.003	0	0	0	0	0.003	0	0	0
Ramphastidae	*Pteroglossus castanotis*	0	0.005	0	0	0	0	0.016	0	0	0	0	0.004	0	0	0
Ramphastidae	*Pteroglossus inscriptus*	0	0.014	0	0	0	0	0.014	0	0	0	0	0.013	0	0	0
Ramphastidae	*Ramphastos tucanus*	0	0.002	0	0	0	0	0.002	0	0	0	0	0.002	0	0	0
Ramphastidae	*Ramphastos vitellinus*	0	0.038	0	0	0	0	0.003	0	0	0	0	0.003	0	0	0
Ramphastidae	*Selenidera reinwardtii*	0	0.001	0	0	0	0	0.000	0	0	0	0	0.000	0	0	0
Steatornithidae	*Steatornis caripensis*	0	0.987	0	0	0	0	0.795	0	0	0	0	0.795	0	0	0
Thraupidae	*Anisognathus igniventris*	0	0.966	0	0	0	0.551	0.993	0	0.993	0	0.878	0.005	0.005	0.005	0.005
Thraupidae	*Anisognathus somptuosus*	0.130	0.894	0.894	0.894	0.894	0.051	0.400	0.400	0.400	0.400	0	0.004	0	0.004	0.004
Thraupidae	*Buthraupis montana*	0.370	0.990	0	0.990	0	0.040	0.807	0.807	0.807	0.807	0	0.078	0	0.078	0.078
Thraupidae	*Catamblyrhynchus diadema*	0.921	0.942	0	0.942	0	0.864	0.953	0.953	0.953	0.953	0	0.571	0	0.571	0.571
Thraupidae	*Chlorochrysa calliparaea*	0	0.996	0	0.996	0.996	0	0.015	0	0	0	0	0.005	0	0	0
Thraupidae	*Chlorophanes spiza*	0	0.186	0	0	0	0	0.004	0	0	0	0	0.005	0	0	0
Thraupidae	*Chlorophonia cyanea*	0	0.599	0	0.599	0.599	0	0.811	0	0	0	0	0.006	0	0	0
Thraupidae	*Chlorornis riefferii*	0.813	0.995	0	0.995	0	0.561	0.993	0.993	0.993	0.993	0	0.009	0	0.009	0.009
Thraupidae	*Chlorospingus canigularis*	0	0.995	0	0	0	0	0.469	0	0	0	0	0.006	0	0	0
Thraupidae	*Chlorospingus flavigularis*	0	0.995	0	0.995	0.995	0	0.015	0	0	0	0	0.003	0	0	0
Thraupidae	*Chlorospingus ophthalmicus*	0.776	0.993	0.993	0.993	0.993	0	0.406	0	0.406	0.406	0	0.009	0	0	0
Thraupidae	*Chlorospingus parvirostris*	0.414	0.971	0.971	0.971	0.971	0	0.762	0	0.762	0.762	0	0.143	0	0	0
Thraupidae	*Cissopis leverianus*	0	0.829	0	0	0	0	0.075	0	0	0	0	0.007	0	0	0
Thraupidae	*Conirostrum cinereum*	0	0.008	0	0	0	0.003	0.337	0	0.337	0	0.014	0.003	0.003	0.003	0.003
Thraupidae	*Conirostrum speciosum*	0	0.247	0	0	0	0	0.093	0	0	0	0	0.009	0	0	0
Thraupidae	*Cyanerpes caeruleus*	0	0.233	0	0	0	0	0.002	0	0	0	0	0.006	0	0	0
Thraupidae	*Cyanerpes cyaneus*	0	0.358	0	0	0	0	0.001	0	0	0	0	0.000	0	0	0
Thraupidae	*Dacnis cayana*	0	0.622	0	0	0	0	0.070	0	0	0	0	0.005	0	0	0
Thraupidae	*Dacnis flaviventer*	0	0.086	0	0	0	0	0.005	0	0	0	0	0.003	0	0	0
Thraupidae	*Dacnis lineata*	0	0.731	0	0	0	0	0.135	0	0	0	0	0.004	0	0	0
Thraupidae	*Delothraupis castaneoventris*	0	0.004	0	0.004	0	0.070	0.039	0.039	0.039	0.039	0	0.003	0.003	0.003	0.003
Thraupidae	*Diglossa brunneiventris*	0	0.002	0	0	0	0.003	0.001	0	0.001	0	0.007	0.044	0.044	0.044	0.044
Thraupidae	*Diglossa caerulescens*	0.977	0.998	0.998	0.998	0.998	0	0.813	0	0.813	0.813	0	0.127	0	0	0
Thraupidae	*Diglossa cyanea*	0.969	0.956	0.956	0.956	0.956	0.044	0.303	0.303	0.303	0.303	0.125	0.003	0.003	0.003	0.003
Thraupidae	*Diglossa glauca*	0	0.969	0	0.969	0.969	0	0.077	0	0	0	0	0.023	0	0	0

Thraupidae	*Diglossa mystacalis*	0	0.002	0	0	0	0.185	0.097	0	0.097	0	0.033	0.003	0.003	0.003	0.003
Thraupidae	*Diglossa sittoides*	0.216	0.375	0.375	0.375	0.375	0.062	0.779	0.779	0.779	0.779	0.004	0.004	0.004	0.004	0.004
Thraupidae	*Dubusia taeniata*	0.863	0.868	0.868	0.868	0.868	0.374	0.731	0.731	0.731	0.731	0	0.919	0	0.919	0.919
Thraupidae	*Euphonia chlorotica*	0	0.095	0	0	0	0	0.010	0	0	0	0	0.004	0	0	0
Thraupidae	*Euphonia chrysopasta*	0	0.051	0	0	0	0	0.017	0	0	0	0	0.017	0	0	0
Thraupidae	*Euphonia laniirostris*	0	0.307	0	0	0	0	0.135	0	0	0	0	0.003	0	0	0
Thraupidae	*Euphonia mesochrysa*	0	0.860	0	0	0	0	0.066	0	0	0	0	0.005	0	0	0
Thraupidae	*Euphonia minuta*	0	0.166	0	0	0	0	0.051	0	0	0	0	0.037	0	0	0
Thraupidae	*Euphonia rufiventris*	0	0.047	0	0	0	0	0.073	0	0	0	0	0.028	0	0	0
Thraupidae	*Euphonia xanthogaster*	0	0.164	0	0.164	0.164	0	0.021	0	0	0	0	0.003	0	0	0
Thraupidae	*Iridophanes pulcherrimus*	0	0.999	0	0	0	0	0.967	0	0	0	0	0.001	0	0	0
Thraupidae	*Iridosornis analis*	0	0.872	0	0.872	0.872	0	0.132	0	0	0	0	0.023	0	0	0
Thraupidae	*Iridosornis jelskii*	0.003	0.052	0	0.052	0	0.195	0.195	0.195	0.195	0.195	0.003	0.005	0.005	0.005	0.005
Thraupidae	*Lamprospiza melanoleuca*	0	0.004	0	0	0	0	0.004	0	0	0	0	0.004	0	0	0
Thraupidae	*Pipraeidea melanonota*	0.273	0.991	0.991	0.991	0.991	0.007	0.929	0.929	0.929	0.929	0	0.003	0	0.003	0.003
Thraupidae	*Piranga leucoptera*	0	0.725	0	0	0	0	0.872	0	0	0	0	0.002	0	0	0
Thraupidae	*Ramphocelus carbo*	0	0.608	0	0	0	0	0.002	0	0	0	0	0.002	0	0	0
Thraupidae	*Ramphocelus nigrogularis*	0	0.003	0	0	0	0	0.002	0	0	0	0	0.003	0	0	0
Thraupidae	*Schistochlamys melanopis*	0.010	0.040	0.040	0.040	0.040	0	0.083	0	0.083	0.083	0	0.010	0	0	0
Thraupidae	*Tachyphonus cristatus*	0	0.017	0	0	0	0	0.005	0	0	0	0	0.003	0	0	0
Thraupidae	*Tachyphonus rufiventer*	0	0.024	0	0	0	0	0.005	0	0	0	0	0.003	0	0	0
Thraupidae	*Tangara arthus*	0	0.598	0	0	0	0	0.014	0	0	0	0	0.003	0	0	0
Thraupidae	*Tangara callophrys*	0	0.006	0	0	0	0	0.007	0	0	0	0	0.006	0	0	0
Thraupidae	*Tangara chilensis*	0	0.580	0	0	0	0	0.004	0	0	0	0	0.004	0	0	0
Thraupidae	*Tangara chrysotis*	0	0.844	0	0.844	0.844	0	0.025	0	0	0	0	0.006	0	0	0
Thraupidae	*Tangara cyanicollis*	0	0.991	0	0.991	0.991	0	0.021	0	0	0	0	0.003	0	0	0
Thraupidae	*Tangara cyanotis*	0	0.889	0	0.889	0.889	0	0.058	0	0	0	0	0.010	0	0	0
Thraupidae	*Tangara gyrola*	0	0.996	0	0	0	0	0.150	0	0	0	0	0.005	0	0	0
Thraupidae	*Tangara mexicana*	0	0.008	0	0	0	0	0.003	0	0	0	0	0.003	0	0	0
Thraupidae	*Tangara nigrocincta*	0	0.137	0	0	0	0	0.074	0	0	0	0	0.008	0	0	0
Thraupidae	*Tangara nigroviridis*	0.973	0.995	0.995	0.995	0.995	0	0.059	0	0.059	0.059	0	0.004	0	0	0
Thraupidae	*Tangara parzudakii*	0	0.996	0	0.996	0.996	0	0.432	0	0	0	0	0.003	0	0	0
Thraupidae	*Tangara punctata*	0	0.586	0	0.586	0.586	0	0.029	0	0	0	0	0.014	0	0	0
Thraupidae	*Tangara ruficervix*	0	0.990	0	0.990	0.990	0	0.660	0	0	0	0	0.005	0	0	0
Thraupidae	*Tangara schrankii*	0	0.038	0	0	0	0	0.002	0	0	0	0	0.002	0	0	0
Thraupidae	*Tangara vassorii*	0.550	0.993	0.993	0.993	0.993	0.972	0.123	0.123	0.123	0.123	0	0.006	0	0.006	0.006
Thraupidae	*Tangara velia*	0	0.003	0	0	0	0	0.003	0	0	0	0	0.003	0	0	0
Thraupidae	*Tangara viridicollis*	0.005	0.002	0.002	0.002	0.002	0	0.005	0	0.005	0.005	0	0.002	0	0	0
Thraupidae	*Tangara xanthocephala*	0.908	0.998	0.998	0.998	0.998	0	0.988	0	0.988	0.988	0	0.003	0	0	0
Thraupidae	*Tangara xanthogastra*	0	0.003	0	0	0	0	0.011	0	0	0	0	0.004	0	0	0
Thraupidae	*Tersina viridis*	0	0.199	0	0	0	0	0.447	0	0	0	0	0.005	0	0	0
Thraupidae	*Thlypopsis sordida*	0	0.239	0	0	0	0	0.639	0	0	0	0	0.002	0	0	0
Thraupidae	*Thraupis bonariensis*	0.002	0.068	0.068	0.068	0.068	0.003	0.299	0.299	0.299	0.299	0.002	0.002	0.002	0.002	0.002
Thraupidae	*Thraupis cyanocephala*	0.934	0.674	0.674	0.674	0.674	0.142	0.023	0.023	0.023	0.023	0	0.002	0	0.002	0.002
Thraupidae	*Thraupis episcopus*	0	0.414	0	0	0	0	0.011	0	0	0	0	0.006	0	0	0
Thraupidae	*Thraupis palmarum*	0	0.981	0	0	0	0	0.093	0	0	0	0	0.003	0	0	0
Thraupidae	*Trichothraupis melanops*	0	0.028	0	0	0	0	0.006	0	0	0	0	0.001	0	0	0
Tinamidae	*Crypturellus atrocapillus*	0	0.007	0	0	0	0	0.007	0	0	0	0	0.008	0	0	0
Tinamidae	*Crypturellus bartletti*	0	0.005	0	0	0	0	0.004	0	0	0	0	0.004	0	0	0
Tinamidae	*Crypturellus cinereus*	0	0.001	0	0	0	0	0.001	0	0	0	0	0.001	0	0	0

Tinamidae	*Crypturellus obsoletus*	0.326	0.010	0.010	0.010	0.010	0.021	0.774	0.774	0.774	0.774	0	0.003	0	0.003	0.003
Tinamidae	*Crypturellus soui*	0	0.065	0	0	0	0	0.057	0	0	0	0	0.003	0	0	0
Tinamidae	*Crypturellus strigulosus*	0	0.000	0	0	0	0	0.000	0	0	0	0	0.001	0	0	0
Tinamidae	*Crypturellus undulatus*	0	0.005	0	0	0	0	0.013	0	0	0	0	0.003	0	0	0
Tinamidae	*Crypturellus variegatus*	0	0.001	0	0	0	0	0.001	0	0	0	0	0.001	0	0	0
Tinamidae	*Nothocercus nigrocapillus*	0.043	0.187	0.187	0.187	0.187	0.049	0.524	0.524	0.524	0.524	0	0.016	0	0.016	0.016
Tinamidae	*Tinamus guttatus*	0	0.001	0	0	0	0	0.001	0	0	0	0	0.001	0	0	0
Tinamidae	*Tinamus major*	0	0.003	0	0	0	0	0.004	0	0	0	0	0.003	0	0	0
Tinamidae	*Tinamus tao*	0	0.002	0	0	0	0	0.002	0	0	0	0	0.002	0	0	0
Trogonidae	*Pharomachrus antisianus*	0	0.981	0	0.981	0.981	0	0.464	0	0	0	0	0.010	0	0	0
Trogonidae	*Pharomachrus auriceps*	0.418	0.992	0.992	0.992	0.992	0.006	0.077	0.077	0.077	0.077	0	0.002	0	0.002	0.002
Trogonidae	*Pharomachrus pavoninus*	0	0.017	0	0	0	0	0.007	0	0	0	0	0.012	0	0	0
Trogonidae	*Trogon collaris*	0	0.892	0	0	0	0	0.271	0	0	0	0	0.007	0	0	0
Trogonidae	*Trogon curucui*	0	0.008	0	0	0	0	0.006	0	0	0	0	0.003	0	0	0
Trogonidae	*Trogon melanurus*	0	0.003	0	0	0	0	0.004	0	0	0	0	0.015	0	0	0
Trogonidae	*Trogon personatus*	0.514	0.967	0.967	0.967	0.967	0.153	0.148	0.148	0.148	0.148	0.048	0.005	0.005	0.005	0.005
Trogonidae	*Trogon violaceus*	0	0.031	0	0	0	0	0.015	0	0	0	0	0.020	0	0	0
Trogonidae	*Trogon viridis*	0	0.043	0	0	0	0	0.009	0	0	0	0	0.005	0	0	0
Turdidae	*Catharus fuscater*	0.046	0.959	0.959	0.959	0.959	0	0.995	0	0.995	0.995	0	0.005	0	0	0
Turdidae	*Entomodestes leucotis*	0.009	0.105	0.105	0.105	0.105	0	0.013	0	0.013	0.013	0	0.002	0	0	0
Turdidae	*Myadestes ralloides*	0.788	0.972	0.972	0.972	0.972	0	0.563	0	0.563	0.563	0	0.002	0	0	0
Turdidae	*Turdus albicollis*	0	0.734	0	0	0	0	0.005	0	0	0	0	0.094	0	0	0
Turdidae	*Turdus chiguanco*	0.003	0.002	0.002	0.002	0.002	0.001	0.008	0.008	0.008	0.008	0.001	0.001	0.001	0.001	0.001
Turdidae	*Turdus fuscater*	0.917	0.991	0	0.991	0	0.014	0.522	0	0.522	0	0.629	0.005	0.005	0.005	0.005
Turdidae	*Turdus hauxwelli*	0	0.249	0	0	0	0	0.004	0	0	0	0	0.004	0	0	0
Turdidae	*Turdus ignobilis*	0	0.766	0	0	0	0	0.031	0	0	0	0	0.004	0	0	0
Turdidae	*Turdus leucops*	0.247	0.994	0.994	0.994	0.994	0	0.979	0	0.979	0.979	0	0.010	0	0	0
Turdidae	*Turdus nigriceps*	0	0.667	0	0	0	0	0.892	0	0	0	0	0.002	0	0	0
Turdidae	*Turdus serranus*	0.739	0.955	0.955	0.955	0.955	0.065	0.638	0.638	0.638	0.638	0	0.010	0	0.010	0.010
Tyrannidae	*Elaenia albiceps*	0.322	0.292	0.292	0.292	0.292	0.128	0.285	0.285	0.285	0.285	0	0.877	0	0.877	0.877
Tyrannidae	*Elaenia flavogaster*	0	0.178	0	0	0	0	0.836	0	0	0	0	0.003	0	0	0
Tyrannidae	*Elaenia gigas*	0	0.019	0	0	0	0	0.054	0	0	0	0	0.018	0	0	0
Tyrannidae	*Elaenia obscura*	0.254	0.351	0.351	0.351	0.351	0.567	0.934	0.934	0.934	0.934	0	0.053	0	0.053	0.053
Tyrannidae	*Elaenia pallatangae*	0.322	0.595	0.595	0.595	0.595	0.015	0.816	0.816	0.816	0.816	0	0.004	0	0.004	0.004
Tyrannidae	*Lophotriccus pileatus*	0	0.315	0	0	0	0	0.625	0	0	0	0	0.004	0	0	0
Tyrannidae	*Mionectes macconnelli*	0	0.003	0	0	0	0	0.002	0	0	0	0	0.003	0	0	0
Tyrannidae	*Mionectes oleagineus*	0	0.822	0	0	0	0	0.003	0	0	0	0	0.002	0	0	0
Tyrannidae	*Mionectes olivaceus*	0	0.197	0	0	0	0	0.599	0	0	0	0	0.003	0	0	0
Tyrannidae	*Mionectes striaticollis*	0.894	0.993	0.993	0.993	0.993	0.139	0.325	0.325	0.325	0.325	0	0.014	0	0.014	0.014
Tyrannidae	*Zimmerius bolivianus*	0.009	0.092	0.092	0.092	0.092	0	0.524	0	0.524	0.524	0	0.001	0	0	0
Tyrannidae	*Zimmerius gracilipes*	0	0.010	0	0	0	0	0.017	0	0	0	0	0.013	0	0	0

Table S5. TSS evaluation of the species distribution models (SDMs). SDMs were fitted for all 232 frugivorous bird species of the Manú Biosphere Reserve (Peru) based on mean annual temperature and precipitation. For each species, five SDM methods were fitted: generalized linear models (GLM), generalized additive models (GAM), boosted regression trees (GBM), random forest (RF) and maximum entropy models (MAXENT). For all SDMs the evaluation metric for the evaluation dataset (20% of total number of occurrences) is given, in addition to the sensitivity and specificity values of the model. The best model per species was chosen by selecting the model with the highest true skill statistic (TSS) evaluation metric, i.e., the sum of sensitivity and specificity minus one. The values provided for RF and GBM models are the mean values of the 10 model runs.

			TSS															
			TSS value of evaluation data					Sensitivity					Specificity					
Family	Bird species	Number of GBIF occurrence records	RF	GBM	GAM	MAXENT	GLM	RF	GBM	GAM	MAXENT	GLM	RF	GBM	GAM	MAXENT	GLM	Best model selection
Cardinalidae	*Cyanocompsa cyanoides*	864	0.700	0.667	0.537	0.555	0.533	0.823	0.835	0.881	0.833	0.845	0.876	0.830	0.654	0.720	0.687	RF
Cardinalidae	*Pheucticus aureoventris*	717	0.732	0.653	0.595	0.642	0.479	0.880	0.817	0.841	0.841	0.783	0.852	0.835	0.754	0.802	0.697	RF
Cardinalidae	*Pheucticus chrysogaster*	1375	0.786	0.667	0.485	0.568	0.426	0.872	0.850	0.887	0.938	0.858	0.913	0.817	0.598	0.629	0.567	RF
Cardinalidae	*Saltator aurantiirostris*	1244	0.767	0.647	0.611	0.653	0.639	0.889	0.921	0.925	0.849	0.921	0.878	0.725	0.690	0.804	0.717	RF
Cardinalidae	*Saltator coerulescens*	2571	0.690	0.582	0.474	0.467	0.488	0.819	0.801	0.785	0.928	0.819	0.871	0.780	0.693	0.539	0.668	RF
Cardinalidae	*Saltator grossus*	1042	0.786	0.753	0.684	0.709	0.672	0.900	0.922	0.861	0.876	0.831	0.886	0.830	0.823	0.833	0.840	RF
Cardinalidae	*Saltator maximus*	3332	0.757	0.619	0.528	0.571	0.527	0.851	0.817	0.827	0.869	0.813	0.906	0.802	0.701	0.699	0.716	RF
Columbidae	*Geotrygon montana*	803	0.713	0.683	0.610	0.620	0.648	0.847	0.876	0.898	0.898	0.904	0.866	0.807	0.710	0.722	0.743	RF
Columbidae	*Patagioenas cayennensis*	2881	0.714	0.575	0.548	0.544	0.499	0.850	0.868	0.825	0.875	0.933	0.864	0.707	0.723	0.673	0.565	RF
Columbidae	*Patagioenas fasciata*	2417	0.794	0.647	0.569	0.605	0.584	0.853	0.870	0.916	0.842	0.835	0.941	0.776	0.653	0.761	0.750	RF
Columbidae	*Patagioenas plumbea*	3003	0.785	0.651	0.600	0.552	0.580	0.856	0.839	0.769	0.853	0.851	0.928	0.812	0.831	0.700	0.727	RF
Columbidae	*Patagioenas subvinacea*	2334	0.758	0.633	0.595	0.592	0.573	0.836	0.828	0.779	0.794	0.736	0.922	0.804	0.816	0.797	0.837	RF
Corvidae	*Cyanocorax cyanomelas*	1213	0.788	0.723	0.632	0.686	0.578	0.907	0.914	0.886	0.916	0.945	0.881	0.809	0.745	0.770	0.633	RF
Corvidae	*Cyanocorax violaceus*	1689	0.869	0.822	0.764	0.826	0.792	0.932	0.952	0.937	0.919	0.928	0.937	0.870	0.828	0.907	0.864	RF
Corvidae	*Cyanocorax yncas*	1911	0.803	0.683	0.623	0.591	0.538	0.871	0.878	0.837	0.784	0.771	0.931	0.804	0.786	0.804	0.767	RF
Cotingidae	*Ampelioides tschudii*	263	0.876	0.831	0.796	0.696	0.633	0.919	0.921	0.923	0.808	0.923	0.957	0.908	0.873	0.888	0.710	RF
Cotingidae	*Ampelion rubrocristatus*	838	0.762	0.702	0.653	0.684	0.673	0.916	0.896	0.927	0.897	0.964	0.846	0.805	0.726	0.787	0.709	RF
Cotingidae	*Ampelion rufaxilla*	103	0.792	0.830	0.719	0.622	0.688	0.930	0.935	0.950	1	0.950	0.857	0.895	0.769	0.622	0.737	GBM
Cotingidae	*Cephalopterus ornatus*	387	0.793	0.791	0.724	0.696	0.714	0.878	0.918	0.921	0.921	0.934	0.913	0.873	0.802	0.774	0.778	RF
Cotingidae	*Conioptilon mcilhennyi*	53	0.936	0.945	0.937	0.986	0.740	0.973	0.973	1	1	1	0.964	0.973	0	0.986	0.740	MAXENT
Cotingidae	*Cotinga cayana*	536	0.822	0.818	0.821	0.847	0.802	0.928	0.920	0.915	0.943	0.962	0.893	0.898	0.906	0.904	0.840	MAXENT
Cotingidae	*Cotinga maynana*	398	0.909	0.884	0.842	0.808	0.857	0.972	0.980	0.937	0.987	0.975	0.936	0.903	0.906	0.817	0.883	RF
Cotingidae	*Gymnoderus foetidus*	812	0.867	0.808	0.794	0.784	0.786	0.943	0.920	0.981	0.969	0.963	0.924	0.887	0.813	0.813	0.824	RF
Cotingidae	*Iodopleura isabellae*	298	0.852	0.828	0.793	0.839	0.732	0.945	0.990	0.914	0.983	0.897	0.907	0.838	0.879	0.856	0.835	RF
Cotingidae	*Laniocera hypopyrra*	284	0.795	0.822	0.762	0.779	0.835	0.921	0.977	0.982	0.857	1	0.870	0.846	0.780	0.922	0.835	GLM
Cotingidae	*Lipaugus vociferans*	1839	0.810	0.783	0.749	0.777	0.748	0.922	0.950	0.972	0.942	0.964	0.888	0.833	0.777	0.835	0.784	RF
Cotingidae	*Oxyruncus cristatus*	141	0.722	0.750	0.678	0.678	0.591	0.846	0.893	0.786	0.857	0.786	0.875	0.854	0.892	0.818	0.805	GBM
Cotingidae	*Pipreola arcuata*	457	0.813	0.712	0.688	0.719	0.677	0.900	0.874	0.890	0.912	0.879	0.913	0.837	0.798	0.807	0.797	RF
Cotingidae	*Pipreola frontalis*	51	0.780	0.770	0.862	0.656	0.864	0.980	0.940	1	0.900	1	0.800	0.840	0.862	0.756	0.863	GLM
Cotingidae	*Pipreola intermedia*	94	0.816	0.721	0.731	0.669	0.537	0.947	0.858	1	0.947	0.895	0.868	0.863	0.731	0.722	0.641	RF
Cotingidae	*Porphyrolaema porphyrolaema*	72	0.900	0.886	0.936	0.956	0.861	0.986	0.986	1	1	0.929	0.914	0.900	0.936	0.956	0.933	MAXENT
Cotingidae	*Querula purpurata*	1181	0.842	0.806	0.776	0.747	0.746	0.932	0.926	0.956	0.876	0.929	0.910	0.881	0.820	0.871	0.816	RF
Cotingidae	*Rupicola peruvianus*	1451	0.845	0.769	0.678	0.728	0.667	0.900	0.871	0.872	0.820	0.830	0.946	0.898	0.807	0.908	0.836	RF
Cotingidae	*Snowornis subalaris*	56	0.900	0.836	0.914	0.786	0.901	0.964	0.900	1	1	1	0.936	0.936	0.915	0.784	0.900	GAM
Cotingidae	*Tityra cayana*	1097	0.730	0.639	0.593	0.568	0.558	0.824	0.917	0.902	0.935	0.930	0.905	0.722	0.691	0.629	0.625	RF

Cotingidae	*Tityra inquisitor*	613	0.694	0.652	0.524	0.571	0.498	0.864	0.879	0.897	0.821	0.974	0.830	0.773	0.627	0.749	0.524	RF
Cotingidae	*Tityra semifasciata*	1308	0.735	0.664	0.623	0.617	0.604	0.877	0.859	0.757	0.749	0.869	0.858	0.803	0.866	0.868	0.735	RF
Cracidae	*Aburria aburri*	410	0.848	0.779	0.711	0.735	0.707	0.918	0.884	0.878	0.817	0.805	0.929	0.890	0.832	0.918	0.901	RF
Cracidae	*Mitu tuberosum*	323	0.853	0.810	0.826	0.849	0.811	0.925	0.886	0.953	0.953	0.906	0.928	0.925	0.872	0.896	0.905	RF
Cracidae	*Ortalis guttata*	2049	0.783	0.685	0.617	0.594	0.603	0.880	0.880	0.807	0.861	0.847	0.903	0.805	0.809	0.734	0.756	RF
Cracidae	*Penelope jacquacu*	1067	0.838	0.795	0.743	0.725	0.727	0.915	0.922	0.953	0.948	0.934	0.923	0.872	0.791	0.776	0.794	RF
Cracidae	*Penelope montagnii*	829	0.796	0.748	0.649	0.666	0.604	0.902	0.932	0.880	0.904	0.892	0.892	0.815	0.770	0.761	0.712	RF
Cracidae	*Pipile cumanensis*	817	0.818	0.781	0.794	0.783	0.688	0.884	0.859	0.919	0.919	0.944	0.933	0.921	0.875	0.864	0.744	RF
Icteridae	*Cacicus cela*	4304	0.793	0.665	0.648	0.645	0.648	0.895	0.911	0.874	0.938	0.893	0.898	0.754	0.773	0.707	0.755	RF
Icteridae	*Cacicus chrysonotus*	643	0.817	0.771	0.702	0.722	0.665	0.916	0.895	0.945	0.898	0.930	0.901	0.876	0.756	0.823	0.734	RF
Icteridae	*Cacicus haemorrhous*	964	0.761	0.647	0.557	0.575	0.579	0.868	0.829	0.915	0.905	0.952	0.893	0.818	0.641	0.670	0.627	RF
Icteridae	*Clypicterus oseryi*	300	0.945	0.930	0.899	0.919	0.856	0.975	0.977	0.983	0.950	0.950	0.970	0.953	0.916	0.969	0.906	RF
Icteridae	*Icterus icterus*	391	0.795	0.800	0.741	0.728	0.704	0.888	0.932	0.857	0.818	0.831	0.906	0.868	0.884	0.909	0.871	GBM
Icteridae	*Molothrus oryzivorus*	1674	0.691	0.603	0.546	0.574	0.505	0.810	0.848	0.878	0.884	0.862	0.881	0.754	0.667	0.689	0.642	RF
Icteridae	*Psarocolius angustifrons*	3654	0.860	0.740	0.674	0.679	0.655	0.922	0.875	0.851	0.818	0.841	0.938	0.864	0.822	0.862	0.814	RF
Icteridae	*Psarocolius atrovirens*	624	0.860	0.697	0.659	0.626	0.582	0.898	0.876	0.808	0.752	0.840	0.962	0.821	0.849	0.874	0.742	RF
Icteridae	*Psarocolius bifasciatus*	667	0.799	0.787	0.769	0.768	0.734	0.895	0.942	0.985	0.886	0.970	0.904	0.844	0.784	0.882	0.764	RF
Icteridae	*Psarocolius decumanus*	3404	0.717	0.549	0.466	0.483	0.472	0.825	0.813	0.918	0.734	0.961	0.892	0.735	0.547	0.743	0.510	RF
Odontophoridae	*Odontophorus balliviani*	57	0.736	0.618	0.646	0.731	0.552	0.955	0.873	1	1	0.909	0.782	0.745	0.646	0.726	0.643	RF
Odontophoridae	*Odontophorus speciosus*	206	0.781	0.685	0.681	0.613	0.671	0.861	0.888	0.854	0.878	0.854	0.912	0.798	0.828	0.734	0.816	RF
Odontophoridae	*Odontophorus stellatus*	236	0.932	0.919	0.879	0.780	0.797	0.962	0.964	1	0.915	1	0.970	0.955	0.879	0.865	0.797	RF
Picidae	*Celeus elegans*	536	0.846	0.830	0.773	0.810	0.755	0.944	0.926	0.924	0.952	0.933	0.901	0.905	0.850	0.858	0.822	RF
Picidae	*Celeus flavus*	474	0.777	0.798	0.766	0.739	0.724	0.948	0.947	0.957	0.946	0.903	0.828	0.849	0.809	0.791	0.820	GBM
Picidae	*Celeus grammicus*	421	0.844	0.842	0.818	0.773	0.823	0.930	0.968	0.902	0.976	0.976	0.913	0.873	0.916	0.796	0.847	RF
Picidae	*Celeus spectabilis*	97	0.921	0.874	0.890	0.842	0.905	0.942	0.895	0.947	0.895	0.947	0.979	0.979	0.943	0.948	0.958	RF
Picidae	*Celeus torquatus*	256	0.818	0.822	0.743	0.776	0.722	0.937	0.978	0.922	0.980	0.941	0.880	0.843	0.822	0.796	0.781	GBM
Picidae	*Colaptes rubiginosus*	1759	0.775	0.679	0.527	0.638	0.538	0.859	0.839	0.710	0.867	0.855	0.915	0.839	0.817	0.771	0.680	RF
Pipridae	*Chiroxiphia boliviana*	340	0.868	0.778	0.653	0.665	0.679	0.944	0.921	0.794	0.838	0.971	0.922	0.857	0.859	0.827	0.708	RF
Pipridae	*Chiroxiphia pareola*	395	0.767	0.777	0.663	0.686	0.555	0.905	0.906	0.910	0.923	0.808	0.862	0.871	0.752	0.762	0.747	GBM
Pipridae	*Lepidothrix coeruleocapilla*	100	0.938	0.913	0.891	0.810	0.841	0.953	0.963	1	1	0.947	0.985	0.950	0.891	0.811	0.893	RF
Pipridae	*Lepidothrix coronata*	981	0.864	0.884	0.796	0.821	0.829	0.938	0.963	0.979	0.989	0.963	0.926	0.921	0.818	0.831	0.866	GBM
Pipridae	*Machaeropterus pyrocephalus*	362	0.879	0.871	0.784	0.770	0.814	0.944	0.953	0.889	0.889	0.944	0.935	0.918	0.895	0.881	0.869	RF
Pipridae	*Manacus manacus*	2059	0.815	0.701	0.628	0.590	0.596	0.890	0.829	0.924	0.934	0.874	0.925	0.872	0.704	0.656	0.722	RF
Pipridae	*Neopelma sulphureiventer*	50	0.880	0.880	0.821	0.681	0.658	0.950	0.990	1	0.800	1	0.930	0.890	0.821	0.881	0.658	RF
Pipridae	*Pipra chloromeros*	370	0.848	0.809	0.742	0.766	0.742	0.925	0.921	0.875	0.847	0.847	0.923	0.888	0.866	0.919	0.895	RF
Pipridae	*Pipra fasciicauda*	670	0.878	0.787	0.761	0.784	0.676	0.948	0.940	0.962	0.985	0.932	0.929	0.847	0.799	0.799	0.745	RF
Pipridae	*Xenopipo holochlora*	165	0.863	0.826	0.821	0.830	0.812	0.969	0.910	1	0.897	0.897	0.894	0.915	0.821	0.933	0.915	RF
Pipridae	*Xenopipo unicolor*	53	0.810	0.852	0.664	0.617	0.510	0.910	0.970	0.800	0.900	0.900	0.900	0.882	0.863	0.716	0.609	GBM
Psittacidae	*Amazona farinosa*	1976	0.809	0.767	0.743	0.730	0.764	0.909	0.908	0.894	0.935	0.948	0.901	0.859	0.849	0.794	0.816	RF
Psittacidae	*Amazona mercenaria*	65	0.709	0.823	0.633	0.643	0.538	0.917	1	0.833	0.750	0.833	0.792	0.823	0.799	0.893	0.705	GBM
Psittacidae	*Amazona ochrocephala*	1458	0.804	0.732	0.711	0.720	0.743	0.898	0.904	0.913	0.941	0.920	0.905	0.827	0.798	0.779	0.823	RF
Psittacidae	*Ara ararauna*	1776	0.816	0.811	0.697	0.705	0.717	0.903	0.945	0.892	0.835	0.934	0.913	0.866	0.804	0.863	0.783	RF
Psittacidae	*Ara chloropterus*	1304	0.790	0.750	0.696	0.720	0.698	0.882	0.877	0.973	0.938	0.923	0.908	0.872	0.723	0.781	0.775	RF
Psittacidae	*Ara macao*	1437	0.860	0.812	0.757	0.826	0.759	0.934	0.910	0.969	0.944	0.986	0.926	0.901	0.788	0.882	0.773	RF
Psittacidae	*Ara militaris*	219	0.759	0.756	0.609	0.543	0.544	0.884	0.965	0.977	0.674	0.907	0.875	0.791	0.631	0.868	0.637	RF
Psittacidae	*Ara severus*	2178	0.825	0.734	0.686	0.666	0.651	0.906	0.913	0.862	0.937	0.836	0.918	0.820	0.824	0.730	0.815	RF
Psittacidae	*Aratinga leucophthalma*	2308	0.740	0.616	0.549	0.552	0.489	0.837	0.836	0.772	0.896	0.905	0.902	0.778	0.777	0.655	0.584	RF
Psittacidae	*Aratinga mitrata*	651	0.803	0.758	0.739	0.736	0.716	0.932	0.933	0.922	0.899	0.907	0.872	0.824	0.816	0.837	0.809	RF
Psittacidae	*Aratinga weddellii*	1240	0.863	0.829	0.770	0.795	0.770	0.945	0.953	0.967	0.975	0.996	0.918	0.877	0.803	0.819	0.774	RF
Psittacidae	*Bolborhynchus lineola*	276	0.835	0.818	0.749	0.837	0.756	0.953	0.956	0.891	0.982	0.964	0.882	0.862	0.858	0.855	0.792	MAXENT

Psittacidae	*Bolborhynchus orbygnesius*	100	0.790	0.805	0.757	0.750	0.564	0.900	0.905	1	1	0.850	0.890	0.900	0.758	0.750	0.714	GBM
Psittacidae	*Brotogeris cyanoptera*	1903	0.881	0.857	0.768	0.774	0.773	0.957	0.952	0.957	0.968	0.899	0.924	0.904	0.811	0.806	0.874	RF
Psittacidae	*Brotogeris sanctithomae*	391	0.883	0.835	0.821	0.887	0.816	0.962	0.938	0.987	0.987	0.974	0.921	0.897	0.834	0.900	0.842	MAXENT
Psittacidae	*Forpus modestus*	296	0.832	0.820	0.901	0.804	0.783	0.915	0.914	0.966	0.915	0.847	0.917	0.907	0.935	0.889	0.936	GAM
Psittacidae	*Nannopsittaca dachilleae*	46	0.922	0.989	0.979	0.973	0.734	0.967	1	1	1	0.889	0.956	0.989	0.979	0.973	0.846	GBM
Psittacidae	*Orthopsittaca manilata*	1040	0.798	0.757	0.734	0.786	0.761	0.921	0.938	0.893	0.922	0.956	0.876	0.819	0.841	0.863	0.805	RF
Psittacidae	*Pionites leucogaster*	476	0.943	0.928	0.891	0.882	0.871	0.975	0.959	0.958	0.926	0.958	0.968	0.968	0.934	0.956	0.913	RF
Psittacidae	*Pionus menstruus*	3293	0.756	0.661	0.644	0.646	0.627	0.873	0.872	0.906	0.925	0.891	0.882	0.789	0.737	0.721	0.735	RF
Psittacidae	*Pionus tumultuosus*	544	0.805	0.762	0.661	0.699	0.678	0.900	0.937	0.944	0.870	0.889	0.905	0.825	0.717	0.828	0.790	RF
Psittacidae	*Primolius couloni*	239	0.923	0.873	0.876	0.877	0.835	0.944	0.937	0.979	0.958	0.896	0.979	0.935	0.897	0.919	0.940	RF
Psittacidae	*Pyrilia barrabandi*	432	0.881	0.888	0.910	0.887	0.849	0.960	0.952	1	0.977	0.965	0.921	0.934	0.911	0.911	0.884	GAM
Psittacidae	*Pyrrhura roseifrons*	117	0.896	0.892	0.899	0.772	0.809	0.978	0.983	1	0.957	1	0.909	0.909	0.899	0.815	0.810	GAM
Psittacidae	*Pyrrhura rupicola*	180	0.903	0.842	0.918	0.763	0.769	0.953	0.897	0.972	0.861	0.889	0.950	0.942	0.946	0.901	0.880	GAM
Psittacidae	*Touit huetii*	140	0.904	0.832	0.808	0.875	0.851	0.954	0.961	0.964	0.964	1	0.950	0.871	0.843	0.911	0.851	RF
Psophiidae	*Psophia leucoptera*	184	0.908	0.895	0.807	0.827	0.863	0.965	0.986	0.946	0.919	0.973	0.943	0.908	0.862	0.909	0.890	RF
Ramphastidae	*Andigena hypoglauca*	365	0.834	0.721	0.611	0.713	0.582	0.903	0.854	0.944	0.903	0.778	0.932	0.867	0.666	0.809	0.804	RF
Ramphastidae	*Aulacorhynchus coeruleicinctis*	238	0.779	0.806	0.644	0.721	0.632	0.881	0.885	0.872	0.872	0.872	0.898	0.921	0.772	0.849	0.759	GBM
Ramphastidae	*Aulacorhynchus derbianus*	201	0.755	0.698	0.566	0.680	0.594	0.838	0.858	0.775	0.925	0.875	0.918	0.840	0.791	0.755	0.719	RF
Ramphastidae	*Aulacorhynchus prasinus*	1122	0.790	0.707	0.591	0.638	0.553	0.908	0.886	0.882	0.869	0.923	0.882	0.820	0.708	0.769	0.630	RF
Ramphastidae	*Capito auratus*	1622	0.869	0.814	0.770	0.781	0.749	0.938	0.947	0.874	0.893	0.877	0.931	0.867	0.896	0.888	0.872	RF
Ramphastidae	*Eubucco richardsoni*	660	0.883	0.870	0.820	0.797	0.827	0.948	0.973	0.962	0.901	0.939	0.935	0.898	0.858	0.896	0.888	RF
Ramphastidae	*Eubucco tucinkae*	86	0.959	0.941	0.924	0.936	0.937	0.982	0.965	0.941	0.941	1	0.976	0.976	0.983	0.995	0.937	RF
Ramphastidae	*Eubucco versicolor*	345	0.826	0.774	0.719	0.683	0.730	0.906	0.875	0.913	0.826	0.928	0.919	0.897	0.806	0.855	0.802	RF
Ramphastidae	*Pteroglossus azara*	499	0.860	0.817	0.739	0.820	0.763	0.937	0.925	0.838	0.970	0.919	0.923	0.892	0.901	0.850	0.844	RF
Ramphastidae	*Pteroglossus beauharnaesii*	265	0.889	0.851	0.824	0.837	0.805	0.953	0.938	0.962	0.943	1	0.934	0.913	0.862	0.894	0.805	RF
Ramphastidae	*Pteroglossus castanotis*	1287	0.775	0.675	0.664	0.663	0.547	0.898	0.944	0.908	0.920	0.944	0.877	0.731	0.756	0.742	0.603	RF
Ramphastidae	*Pteroglossus inscriptus*	496	0.814	0.824	0.749	0.783	0.801	0.922	0.977	0.979	0.958	0.895	0.892	0.847	0.770	0.826	0.907	GBM
Ramphastidae	*Ramphastos tucanus*	2555	0.815	0.796	0.756	0.781	0.746	0.934	0.938	0.943	0.941	0.927	0.881	0.857	0.813	0.840	0.819	RF
Ramphastidae	*Ramphastos vitellinus*	1703	0.761	0.672	0.629	0.683	0.634	0.887	0.841	0.768	0.934	0.831	0.874	0.830	0.860	0.751	0.803	RF
Ramphastidae	*Selenidera reinwardtii*	598	0.868	0.845	0.761	0.776	0.742	0.944	0.962	0.958	0.924	0.856	0.924	0.883	0.803	0.852	0.886	RF
Steatornithidae	*Steatornis caripensis*	203	0.645	0.463	0.568	0.551	0.539	0.763	0.583	0.875	0.925	0.825	0.883	0.878	0.693	0.625	0.714	RF
Thraupidae	*Anisognathus igniventris*	1405	0.841	0.769	0.706	0.699	0.698	0.911	0.901	0.898	0.814	0.938	0.929	0.868	0.807	0.886	0.760	RF
Thraupidae	*Anisognathus somptuosus*	2194	0.855	0.765	0.748	0.717	0.676	0.915	0.867	0.896	0.863	0.866	0.939	0.897	0.851	0.854	0.809	RF
Thraupidae	*Buthraupis montana*	995	0.834	0.768	0.696	0.747	0.667	0.913	0.942	0.878	0.919	0.873	0.921	0.826	0.816	0.828	0.793	RF
Thraupidae	*Catamblyrhynchus diadema*	459	0.762	0.764	0.613	0.677	0.685	0.876	0.938	0.844	0.911	0.889	0.886	0.826	0.769	0.765	0.796	GBM
Thraupidae	*Chlorochrysa calliparaea*	491	0.877	0.841	0.752	0.760	0.772	0.913	0.934	0.969	0.898	1	0.963	0.907	0.783	0.862	0.772	RF
Thraupidae	*Chlorophanes spiza*	2100	0.772	0.684	0.599	0.579	0.564	0.888	0.861	0.949	0.915	0.866	0.883	0.823	0.650	0.663	0.698	RF
Thraupidae	*Chlorophonia cyanea*	976	0.772	0.648	0.665	0.653	0.653	0.881	0.930	0.894	0.788	0.841	0.891	0.716	0.771	0.864	0.812	RF
Thraupidae	*Chlorornis riefferii*	929	0.830	0.768	0.691	0.742	0.667	0.899	0.891	0.780	0.852	0.852	0.930	0.877	0.911	0.889	0.814	RF
Thraupidae	*Chlorospingus canigularis*	487	0.821	0.787	0.644	0.609	0.565	0.910	0.918	0.969	0.948	0.781	0.910	0.868	0.675	0.660	0.783	RF
Thraupidae	*Chlorospingus flavigularis*	1090	0.909	0.823	0.674	0.729	0.728	0.949	0.910	0.877	0.791	0.829	0.961	0.912	0.797	0.938	0.898	RF
Thraupidae	*Chlorospingus ophthalmicus*	1665	0.826	0.649	0.543	0.579	0.491	0.892	0.900	0.823	0.856	0.878	0.934	0.749	0.720	0.726	0.613	RF
Thraupidae	*Chlorospingus parvirostris*	193	0.826	0.826	0.727	0.685	0.598	0.921	0.938	0.949	0.897	0.821	0.905	0.887	0.778	0.787	0.776	GBM
Thraupidae	*Cissopis leverianus*	1759	0.787	0.681	0.624	0.653	0.628	0.875	0.836	0.944	0.868	0.895	0.911	0.845	0.680	0.782	0.733	RF
Thraupidae	*Conirostrum cinereum*	1979	0.799	0.735	0.653	0.679	0.546	0.883	0.884	0.885	0.813	0.932	0.915	0.851	0.767	0.865	0.613	RF
Thraupidae	*Conirostrum speciosum*	553	0.710	0.661	0.604	0.581	0.493	0.806	0.920	0.848	0.981	0.924	0.904	0.741	0.755	0.598	0.569	RF
Thraupidae	*Cyanerpes caeruleus*	1633	0.769	0.658	0.589	0.624	0.531	0.869	0.875	0.913	0.875	0.791	0.898	0.782	0.675	0.748	0.740	RF
Thraupidae	*Cyanerpes cyaneus*	492	0.754	0.670	0.571	0.520	0.570	0.916	0.912	0.894	1	0.926	0.838	0.758	0.678	0.520	0.645	RF
Thraupidae	*Dacnis cayana*	2543	0.682	0.570	0.504	0.532	0.442	0.821	0.849	0.911	0.921	0.929	0.859	0.720	0.593	0.611	0.512	RF
Thraupidae	*Dacnis flaviventer*	556	0.836	0.829	0.772	0.801	0.828	0.939	0.941	0.937	0.946	0.955	0.897	0.886	0.835	0.855	0.872	RF

Thraupidae	*Dacnis lineata*	1418	0.766	0.720	0.601	0.598	0.629	0.858	0.893	0.888	0.870	0.775	0.908	0.825	0.713	0.727	0.853	RF
Thraupidae	*Delothraupis castaneoventris*	238	0.804	0.732	0.720	0.763	0.631	0.904	0.938	0.915	0.872	0.851	0.900	0.794	0.806	0.891	0.780	RF
Thraupidae	*Diglossa brunneiventris*	851	0.823	0.794	0.703	0.674	0.634	0.937	0.963	0.888	0.905	0.858	0.885	0.831	0.815	0.768	0.777	RF
Thraupidae	*Diglossa caerulescens*	633	0.802	0.742	0.624	0.643	0.634	0.931	0.938	0.848	0.808	0.896	0.870	0.804	0.775	0.835	0.736	RF
Thraupidae	*Diglossa cyanea*	2564	0.851	0.766	0.676	0.707	0.694	0.923	0.885	0.866	0.957	0.953	0.927	0.881	0.810	0.750	0.741	RF
Thraupidae	*Diglossa glauca*	282	0.858	0.871	0.848	0.827	0.813	0.938	0.965	0.927	0.927	0.964	0.920	0.905	0.920	0.900	0.849	GBM
Thraupidae	*Diglossa mystacalis*	283	0.832	0.786	0.674	0.609	0.750	0.913	0.904	0.964	0.821	0.875	0.919	0.881	0.709	0.788	0.875	RF
Thraupidae	*Diglossa sittoides*	680	0.721	0.607	0.513	0.545	0.534	0.886	0.854	0.858	0.799	0.806	0.835	0.753	0.655	0.746	0.728	RF
Thraupidae	*Dubusia taeniata*	416	0.806	0.748	0.760	0.677	0.730	0.917	0.952	0.951	0.878	0.890	0.889	0.795	0.809	0.798	0.840	RF
Thraupidae	*Euphonia chlorotica*	994	0.647	0.638	0.486	0.518	0.463	0.844	0.909	0.938	0.928	0.778	0.801	0.728	0.548	0.589	0.683	RF
Thraupidae	*Euphonia chrysopasta*	774	0.799	0.809	0.763	0.765	0.768	0.893	0.903	0.948	0.922	0.967	0.906	0.905	0.814	0.844	0.801	GBM
Thraupidae	*Euphonia laniirostris*	2821	0.738	0.601	0.411	0.487	0.331	0.826	0.823	0.698	0.704	0.582	0.911	0.776	0.712	0.783	0.749	RF
Thraupidae	*Euphonia mesochrysa*	348	0.794	0.697	0.653	0.614	0.638	0.899	0.836	0.914	0.886	0.914	0.893	0.861	0.739	0.728	0.724	RF
Thraupidae	*Euphonia minuta*	349	0.723	0.718	0.676	0.738	0.622	0.875	0.891	0.928	0.870	0.841	0.847	0.827	0.747	0.868	0.782	MAXENT
Thraupidae	*Euphonia rufiventris*	716	0.830	0.805	0.813	0.813	0.736	0.914	0.904	0.930	0.965	0.894	0.915	0.901	0.883	0.846	0.842	RF
Thraupidae	*Euphonia xanthogaster*	3722	0.837	0.691	0.597	0.646	0.602	0.904	0.861	0.737	0.799	0.946	0.933	0.828	0.862	0.847	0.656	RF
Thraupidae	*Iridophanes pulcherrimus*	198	0.879	0.839	0.753	0.793	0.790	0.964	0.979	0.923	0.974	1	0.915	0.860	0.829	0.817	0.789	RF
Thraupidae	*Iridosornis analis*	268	0.841	0.765	0.778	0.808	0.723	0.919	0.911	0.962	0.962	0.887	0.922	0.852	0.815	0.845	0.836	RF
Thraupidae	*Iridosornis jelskii*	150	0.840	0.803	0.639	0.690	0.668	0.920	0.870	0.867	0.933	0.833	0.920	0.930	0.773	0.756	0.834	RF
Thraupidae	*Lamprospiza melanoleuca*	151	0.830	0.840	0.880	0.773	0.834	0.950	0.973	1	1	1	0.880	0.867	0.880	0.774	0.834	GAM
Thraupidae	*Pipraeidea melanonota*	1333	0.752	0.642	0.568	0.570	0.593	0.856	0.816	0.773	0.885	0.777	0.896	0.825	0.795	0.685	0.816	RF
Thraupidae	*Piranga leucoptera*	537	0.797	0.699	0.653	0.695	0.686	0.892	0.819	0.876	0.876	0.943	0.904	0.878	0.777	0.819	0.744	RF
Thraupidae	*Ramphocelus carbo*	5139	0.774	0.600	0.560	0.575	0.527	0.870	0.874	0.917	0.864	0.754	0.904	0.725	0.644	0.711	0.772	RF
Thraupidae	*Ramphocelus nigrogularis*	872	0.892	0.881	0.853	0.863	0.865	0.963	0.956	0.959	0.971	0.959	0.929	0.924	0.894	0.892	0.906	RF
Thraupidae	*Schistochlamys melanopis*	514	0.658	0.642	0.507	0.545	0.515	0.856	0.908	0.772	0.911	0.941	0.799	0.733	0.735	0.633	0.574	RF
Thraupidae	*Tachyphonus cristatus*	700	0.749	0.723	0.640	0.634	0.658	0.887	0.953	0.876	0.883	0.883	0.862	0.769	0.764	0.751	0.774	RF
Thraupidae	*Tachyphonus rufiventer*	201	0.763	0.763	0.692	0.630	0.609	0.870	0.938	0.900	0.925	0.975	0.893	0.823	0.791	0.705	0.634	RF
Thraupidae	*Tangara arthus*	2792	0.890	0.794	0.672	0.711	0.679	0.935	0.924	0.796	0.918	0.767	0.955	0.869	0.876	0.794	0.911	RF
Thraupidae	*Tangara callophrys*	356	0.898	0.918	0.882	0.832	0.842	0.967	0.991	1	0.929	0.900	0.930	0.927	0.881	0.903	0.942	GBM
Thraupidae	*Tangara chilensis*	2072	0.812	0.720	0.654	0.687	0.605	0.912	0.895	0.820	0.908	0.813	0.900	0.824	0.835	0.780	0.792	RF
Thraupidae	*Tangara chrysotis*	315	0.865	0.830	0.748	0.735	0.760	0.938	0.924	1	0.968	0.952	0.925	0.906	0.749	0.767	0.808	RF
Thraupidae	*Tangara cyanicollis*	2781	0.828	0.700	0.631	0.686	0.629	0.901	0.867	0.855	0.832	0.856	0.927	0.832	0.776	0.854	0.772	RF
Thraupidae	*Tangara cyanotis*	104	0.800	0.800	0.812	0.752	0.758	0.929	0.910	1	1	0.905	0.871	0.890	0.812	0.751	0.853	GAM
Thraupidae	*Tangara gyrola*	3030	0.821	0.677	0.622	0.620	0.608	0.900	0.848	0.861	0.900	0.922	0.921	0.829	0.760	0.720	0.686	RF
Thraupidae	*Tangara mexicana*	1121	0.761	0.704	0.676	0.676	0.667	0.902	0.889	0.959	0.968	0.950	0.859	0.814	0.716	0.707	0.716	RF
Thraupidae	*Tangara nigrocincta*	477	0.792	0.737	0.696	0.731	0.587	0.900	0.885	0.853	0.916	0.905	0.892	0.849	0.843	0.816	0.681	RF
Thraupidae	*Tangara nigroviridis*	2226	0.877	0.731	0.733	0.784	0.690	0.930	0.877	0.859	0.895	0.893	0.946	0.853	0.874	0.889	0.798	RF
Thraupidae	*Tangara parzudakii*	1116	0.889	0.792	0.787	0.747	0.745	0.931	0.912	0.905	0.873	0.914	0.958	0.880	0.882	0.874	0.830	RF
Thraupidae	*Tangara punctata*	629	0.790	0.674	0.612	0.639	0.622	0.872	0.834	0.752	0.768	0.720	0.918	0.840	0.860	0.871	0.902	RF
Thraupidae	*Tangara ruficervix*	1112	0.877	0.816	0.749	0.782	0.727	0.925	0.916	0.886	0.891	0.864	0.952	0.900	0.863	0.889	0.863	RF
Thraupidae	*Tangara schrankii*	1400	0.856	0.800	0.727	0.719	0.677	0.932	0.955	0.896	0.892	0.928	0.923	0.845	0.832	0.827	0.749	RF
Thraupidae	*Tangara vassorii*	1355	0.825	0.720	0.675	0.662	0.658	0.887	0.904	0.926	0.941	0.926	0.937	0.815	0.749	0.721	0.732	RF
Thraupidae	*Tangara velia*	423	0.776	0.769	0.727	0.725	0.748	0.872	0.969	0.940	0.904	0.964	0.904	0.800	0.787	0.821	0.785	RF
Thraupidae	*Tangara viridicollis*	436	0.856	0.849	0.823	0.807	0.776	0.938	0.938	0.942	0.930	0.895	0.917	0.909	0.880	0.877	0.878	RF
Thraupidae	*Tangara xanthocephala*	1354	0.840	0.772	0.731	0.724	0.698	0.926	0.895	0.860	0.849	0.860	0.914	0.876	0.870	0.874	0.838	RF
Thraupidae	*Tangara xanthogastra*	509	0.803	0.771	0.712	0.716	0.650	0.893	0.881	0.949	0.818	0.798	0.910	0.890	0.763	0.897	0.852	RF
Thraupidae	*Tersina viridis*	2109	0.757	0.673	0.593	0.635	0.601	0.864	0.902	0.881	0.873	0.908	0.893	0.771	0.713	0.762	0.693	RF
Thraupidae	*Thlypopsis sordida*	418	0.630	0.609	0.503	0.432	0.492	0.872	0.896	0.861	0.886	0.937	0.758	0.711	0.641	0.546	0.554	RF
Thraupidae	*Thraupis bonariensis*	1634	0.747	0.612	0.550	0.606	0.562	0.870	0.892	0.830	0.886	0.839	0.876	0.720	0.721	0.720	0.723	RF
Thraupidae	*Thraupis cyanocephala*	1810	0.816	0.706	0.625	0.671	0.598	0.884	0.884	0.916	0.876	0.955	0.933	0.821	0.709	0.795	0.643	RF

Thraupidae	*Thraupis episcopus*	9138	0.750	0.507	0.370	0.456	0.369	0.832	0.768	0.697	0.729	0.622	0.918	0.738	0.674	0.727	0.746	RF
Thraupidae	*Thraupis palmarum*	6090	0.730	0.566	0.484	0.506	0.464	0.836	0.871	0.830	0.935	0.813	0.893	0.694	0.653	0.572	0.649	RF
Thraupidae	*Trichothraupis melanops*	1032	0.861	0.777	0.754	0.775	0.739	0.938	0.930	0.922	0.887	0.877	0.922	0.846	0.831	0.888	0.862	RF
Tinamidae	*Crypturellus atrocapillus*	275	0.869	0.876	0.776	0.773	0.720	0.904	0.965	0.945	0.836	0.836	0.965	0.911	0.831	0.936	0.884	GBM
Tinamidae	*Crypturellus bartletti*	282	0.914	0.902	0.878	0.875	0.868	0.964	0.964	1	0.929	0.964	0.950	0.938	0.878	0.947	0.904	RF
Tinamidae	*Crypturellus cinereus*	925	0.854	0.812	0.794	0.814	0.757	0.938	0.935	0.923	0.945	0.945	0.915	0.876	0.871	0.870	0.813	RF
Tinamidae	*Crypturellus obsoletus*	663	0.775	0.627	0.572	0.621	0.578	0.881	0.872	0.879	0.924	0.871	0.894	0.754	0.693	0.696	0.706	RF
Tinamidae	*Crypturellus soui*	1834	0.750	0.654	0.585	0.562	0.605	0.857	0.870	0.864	0.756	0.861	0.893	0.784	0.720	0.805	0.742	RF
Tinamidae	*Crypturellus strigulosus*	112	0.855	0.832	0.842	0.725	0.821	0.955	0.973	0.955	0.955	1	0.900	0.859	0.887	0.769	0.822	RF
Tinamidae	*Crypturellus undulatus*	1657	0.840	0.743	0.749	0.767	0.709	0.901	0.937	0.944	0.904	0.963	0.939	0.805	0.805	0.864	0.747	RF
Tinamidae	*Crypturellus variegatus*	447	0.825	0.816	0.782	0.763	0.732	0.915	0.969	0.955	0.876	0.933	0.910	0.847	0.827	0.887	0.800	RF
Tinamidae	*Nothocercus nigrocapillus*	122	0.829	0.767	0.762	0.748	0	0.929	0.942	1	1	0	0.900	0.825	0	0.748	0	RF
Tinamidae	*Tinamus guttatus*	300	0.882	0.822	0.825	0.880	0.836	0.958	0.925	0.933	0.983	0.983	0.923	0.895	0.891	0.896	0.853	RF
Tinamidae	*Tinamus major*	1034	0.807	0.776	0.768	0.772	0.748	0.877	0.888	0.975	0.871	0.876	0.929	0.888	0.793	0.901	0.872	RF
Tinamidae	*Tinamus tao*	255	0.783	0.789	0.664	0.651	0.644	0.934	0.940	0.940	0.840	0.880	0.849	0.849	0.724	0.809	0.764	GBM
Trogonidae	*Pharomachrus antisianus*	564	0.823	0.802	0.756	0.775	0.763	0.904	0.892	0.881	0.927	0.936	0.919	0.910	0.876	0.847	0.826	RF
Trogonidae	*Pharomachrus auriceps*	1413	0.864	0.804	0.712	0.735	0.738	0.924	0.913	0.867	0.885	0.867	0.939	0.891	0.845	0.850	0.871	RF
Trogonidae	*Pharomachrus pavoninus*	222	0.866	0.798	0.797	0.736	0.738	0.948	0.870	0.909	0.841	0.909	0.918	0.927	0.888	0.895	0.829	RF
Trogonidae	*Trogon collaris*	1355	0.779	0.671	0.592	0.575	0.554	0.896	0.857	0.848	0.726	0.760	0.882	0.813	0.744	0.849	0.793	RF
Trogonidae	*Trogon curucui*	1157	0.770	0.714	0.647	0.586	0.539	0.883	0.914	0.912	0.820	0.939	0.886	0.801	0.734	0.766	0.600	RF
Trogonidae	*Trogon melanurus*	1422	0.802	0.752	0.730	0.744	0.717	0.918	0.894	0.910	0.914	0.953	0.883	0.858	0.819	0.830	0.764	RF
Trogonidae	*Trogon personatus*	1686	0.818	0.767	0.592	0.682	0.628	0.892	0.879	0.722	0.784	0.808	0.926	0.888	0.869	0.897	0.820	RF
Trogonidae	*Trogon violaceus*	222	0.729	0.734	0.693	0.648	0.718	0.923	0.955	0.864	0.864	0.977	0.807	0.780	0.828	0.785	0.740	GBM
Trogonidae	*Trogon viridis*	1592	0.752	0.696	0.636	0.631	0.666	0.883	0.912	0.871	0.852	0.868	0.868	0.784	0.765	0.778	0.798	RF
Turdidae	*Catharus fuscater*	380	0.802	0.745	0.678	0.600	0.619	0.885	0.873	0.838	0.797	0.784	0.917	0.872	0.839	0.802	0.835	RF
Turdidae	*Entomodestes leucotis*	334	0.799	0.752	0.705	0.708	0.651	0.890	0.888	0.925	0.925	0.955	0.909	0.864	0.780	0.782	0.695	RF
Turdidae	*Myadestes ralloides*	2199	0.885	0.744	0.672	0.685	0.651	0.934	0.885	0.848	0.841	0.823	0.951	0.859	0.827	0.842	0.827	RF
Turdidae	*Turdus albicollis*	1473	0.768	0.695	0.532	0.584	0.550	0.868	0.856	0.783	0.838	0.752	0.899	0.837	0.749	0.746	0.798	RF
Turdidae	*Turdus chiguanco*	2306	0.786	0.664	0.585	0.640	0.574	0.900	0.944	0.951	0.881	0.945	0.885	0.720	0.634	0.756	0.629	RF
Turdidae	*Turdus fuscater*	4362	0.813	0.700	0.594	0.624	0.636	0.882	0.881	0.895	0.829	0.940	0.930	0.819	0.699	0.795	0.696	RF
Turdidae	*Turdus hauxwelli*	577	0.850	0.833	0.822	0.828	0.746	0.912	0.908	0.922	0.930	0.870	0.937	0.925	0.900	0.898	0.877	RF
Turdidae	*Turdus ignobilis*	3427	0.790	0.661	0.642	0.625	0.623	0.877	0.877	0.839	0.771	0.805	0.912	0.784	0.803	0.854	0.817	RF
Turdidae	*Turdus leucops*	270	0.819	0.774	0.645	0.747	0.661	0.904	0.854	0.833	0.963	0.926	0.913	0.920	0.811	0.784	0.735	RF
Turdidae	*Turdus nigriceps*	270	0.755	0.679	0.657	0.542	0.597	0.849	0.882	0.922	0.980	0.882	0.902	0.796	0.736	0.561	0.715	RF
Turdidae	*Turdus serranus*	1312	0.786	0.730	0.656	0.686	0.637	0.883	0.904	0.808	0.866	0.862	0.903	0.826	0.846	0.820	0.775	RF
Tyrannidae	*Elaenia albiceps*	4855	0.783	0.635	0.550	0.634	0.543	0.886	0.883	0.916	0.869	0.829	0.896	0.752	0.635	0.766	0.714	RF
Tyrannidae	*Elaenia flavogaster*	2433	0.641	0.479	0.417	0.404	0.433	0.754	0.865	0.868	0.895	0.935	0.887	0.613	0.550	0.509	0.498	RF
Tyrannidae	*Elaenia gigas*	291	0.837	0.694	0.723	0.721	0.726	0.926	0.854	0.825	0.895	0.860	0.910	0.840	0.898	0.823	0.866	RF
Tyrannidae	*Elaenia obscura*	374	0.703	0.750	0.599	0.641	0.596	0.847	0.908	0.889	0.903	0.903	0.856	0.841	0.711	0.738	0.693	GBM
Tyrannidae	*Elaenia pallatangae*	772	0.786	0.718	0.576	0.670	0.623	0.886	0.878	0.883	0.857	0.929	0.900	0.836	0.693	0.812	0.693	RF
Tyrannidae	*Lophotriccus pileatus*	1337	0.858	0.772	0.639	0.712	0.621	0.930	0.905	0.840	0.938	0.907	0.927	0.868	0.798	0.775	0.714	RF
Tyrannidae	*Mionectes macconnelli*	306	0.782	0.694	0.721	0.651	0.643	0.917	0.858	0.883	0.817	0.867	0.866	0.836	0.838	0.833	0.776	RF
Tyrannidae	*Mionectes oleagineus*	1767	0.779	0.713	0.661	0.595	0.676	0.874	0.909	0.873	0.746	0.870	0.905	0.803	0.788	0.849	0.806	RF
Tyrannidae	*Mionectes olivaceus*	1252	0.834	0.725	0.655	0.660	0.549	0.917	0.854	0.951	0.827	0.646	0.916	0.870	0.704	0.831	0.902	RF
Tyrannidae	*Mionectes striaticollis*	1558	0.813	0.722	0.629	0.664	0.661	0.890	0.868	0.840	0.918	0.830	0.922	0.854	0.788	0.744	0.831	RF
Tyrannidae	*Zimmerius bolivianus*	197	0.869	0.831	0.694	0.649	0.683	0.936	0.959	1	0.897	0.846	0.933	0.872	0.694	0.752	0.837	RF
Tyrannidae	*Zimmerius gracilipes*	519	0.786	0.763	0.762	0.696	0.746	0.926	0.900	0.912	0.833	0.922	0.860	0.863	0.850	0.863	0.825	RF

Table S6. Pearson's correlation coefficients between ten morphological traits which are relevant for seed dispersal by frugivorous birds.

	Bill width	Bill length	Bill height	Wing length	Kipp’s distance	Tail length	Tarsus length	Tarsus width (sagittal)	Tarsus width (lateral)
Body mass	0,82	0,79	0,8	0,96	0,78	0,76	0,72	0,96	0,95
Bill width		0,88	0,81	0,82	0,65	0,77	0,55	0,76	0,77
Bill length			0,75	0,76	0,52	0,69	0,71	0,76	0,7
Bill height				0,83	0,72	0,79	0,45	0,81	0,87
Wing length					0,86	0,85	0,63	0,93	0,94
Kipp’s distance						0,7	0,24	0,72	0,83
Tail length							0,5	0,77	0,74
Tarsus length								0,78	0,58
Tarsus width (sagittal)									0,94

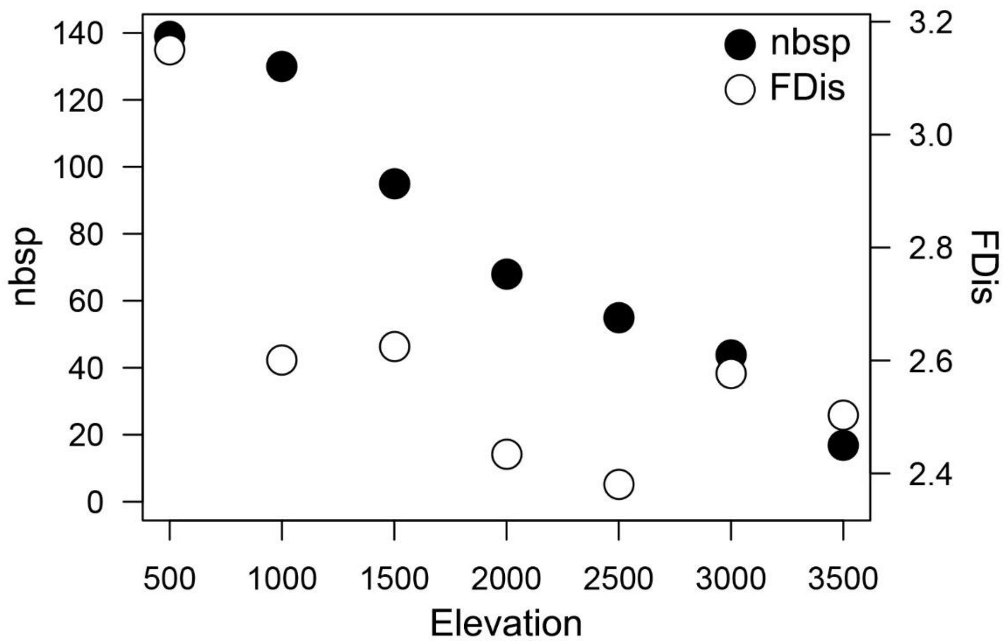

Figure S1. Species richness and functional dispersion under current conditions along the forested part of an elevational gradient in the Peruvian Andes. Current values of species richness (nbsp, closed circles) and functional dispersion (FDis, open circles) of bird assemblages (*n* = 232 species) do not show a statistically significant correlation (Pearson *r* = 0.54, *P* = 0.21).

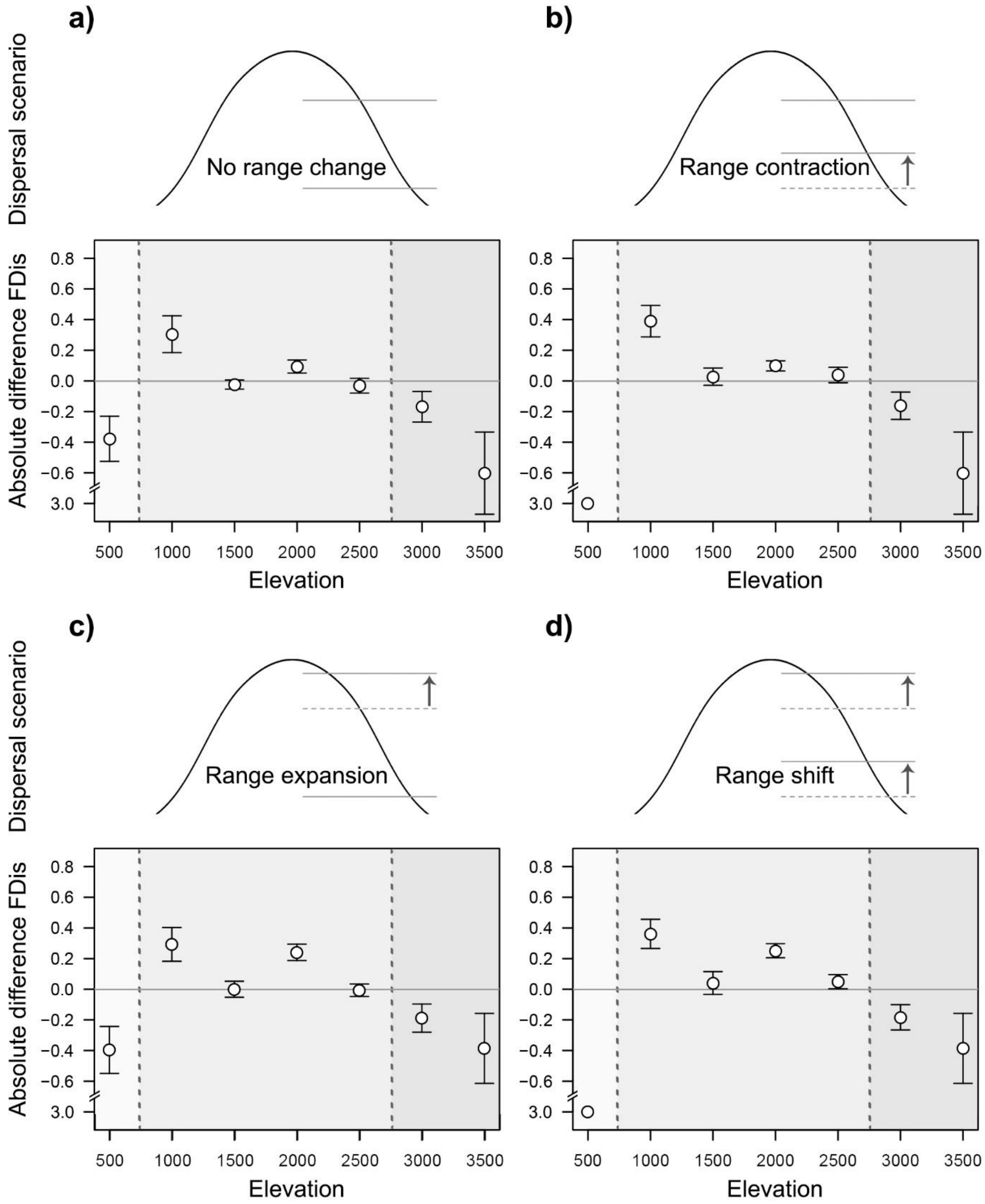

Figure S2. Potential changes in functional dispersion (FDis) of bird assemblages under projected future climate change (based on the RCP 6.0 scenario). Shown are four potential dispersal scenarios representing (a) *no range change*, (b) *range contraction* (lower range limit moves upwards), (c) *range expansion* (upper range limit moves upwards), and (d) *range shift* (both lower and upper range limit move upwards), with the lower panels showing the

projected absolute changes in FDis, for each of seven elevational levels, representing a gradient from 500 m to 3500 m (at 500 m intervals). Absolute changes in FDis are based on species distribution models (SDMs) of 232 birds under current and projected future climate change scenarios using representative concentration pathway RCP 8.5 and the respective dispersal scenario. The values represent mean and standard deviation of the results derived from five general circulation models (CC, HE, MC, MG, NO). FDis calculations were derived from the first five PCoA axes, computed from ten morphological bird traits and are based on the SDM-derived probability of occurrence of bird species. Positive FDis values indicate an increase of FDis under climate change, and negative values a decrease. Projected future assemblages at 500 m were completely depleted of species under range contraction and shift scenarios.

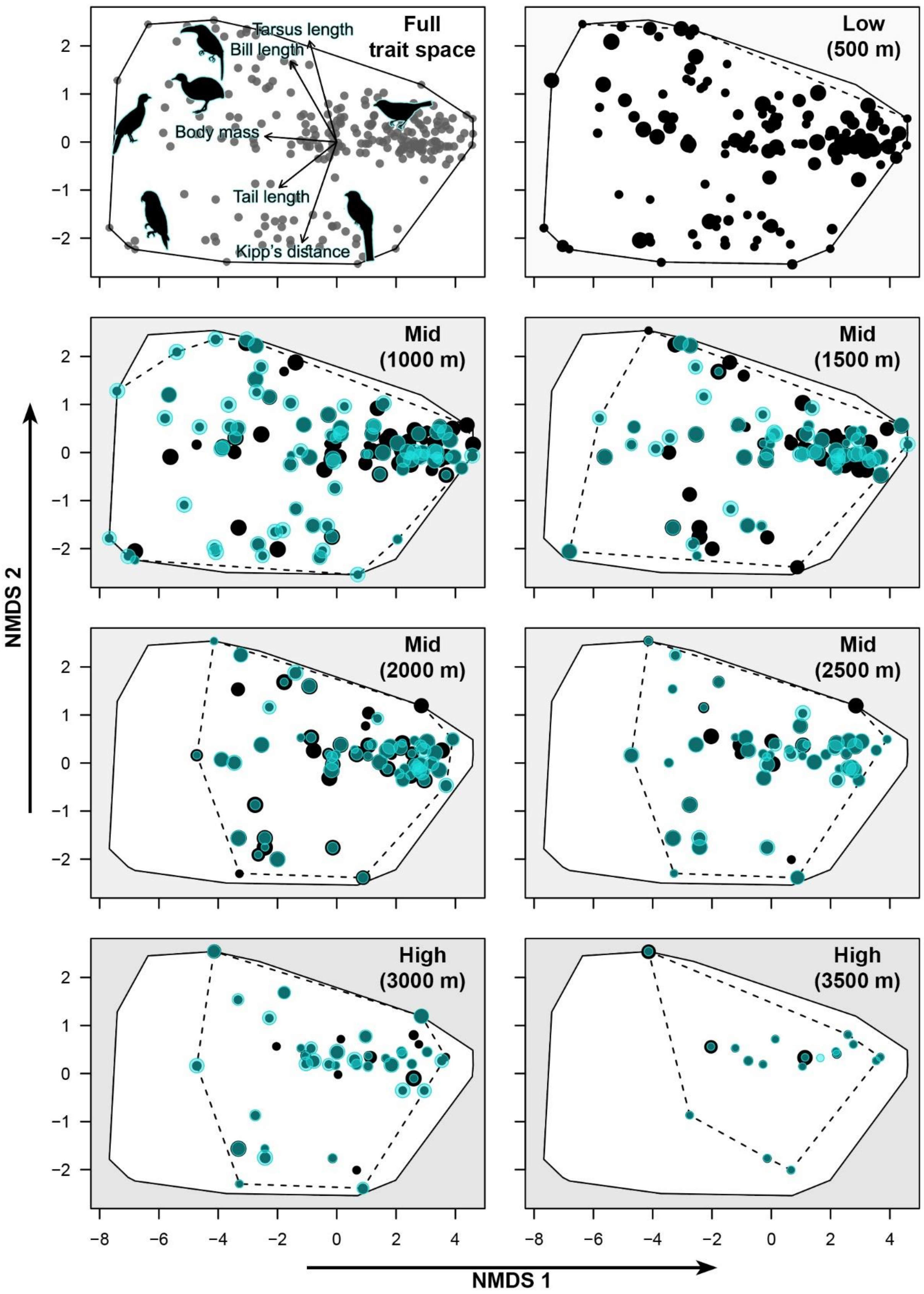

Figure S3. Projected changes in functional trait spaces of frugivorous bird assemblages under climate change assuming a *range contraction scenario*. The functional trait space is derived

from Nonmetric Multidimensional Scaling (NMDS, based on Euclidean distances) which condenses the dissimilarity of the first five PCoA axes, computed from ten morphological bird traits (bill width, bill length, bill height, tail length, tarsus length, tarsus sagittal width, tarsus lateral width, wing length, Kipp's distance and body mass) onto two axes for visualization (NMDS 1, NMDS 2). Trait spaces are shown for the entire bird assemblage (upper left) and for assemblages on each of the seven elevational levels (500 m to 3500 m, at 500 m intervals). Black polygons illustrate the functional trait space of the entire bird assemblage. Black stippled polygons illustrate the functional trait space of the current local assemblage at the given elevation. Each dot represents one bird species (n = 232). Within each elevational level, dot size represents the probability of occurrence as derived from SDMs. Black dots represent current assemblages, transparent green dots represent occurrence probabilities under projected climate change (based on the RCP 8.5 scenario for 2080, MIROC5 climate model). Species-level changes are visible in four ways: (1) complete black dots indicate future loss of species, (2) complete light green dots indicate future immigration of species, (3) dark green dots with a black ring indicate future decline in occurrence probability, and (4) dark green dots with a light green ring indicate future increase in occurrence probability. Bird silhouettes in the upper left panel indicate the approximate location of key taxonomic groups in trait space (counterclockwise from top): toucans, tinamous, guans, parrots, trogons and tanagers. Fitted trait arrows indicate the relationship between five morphological bird traits (representing the different types of traits) and the ordination axes.

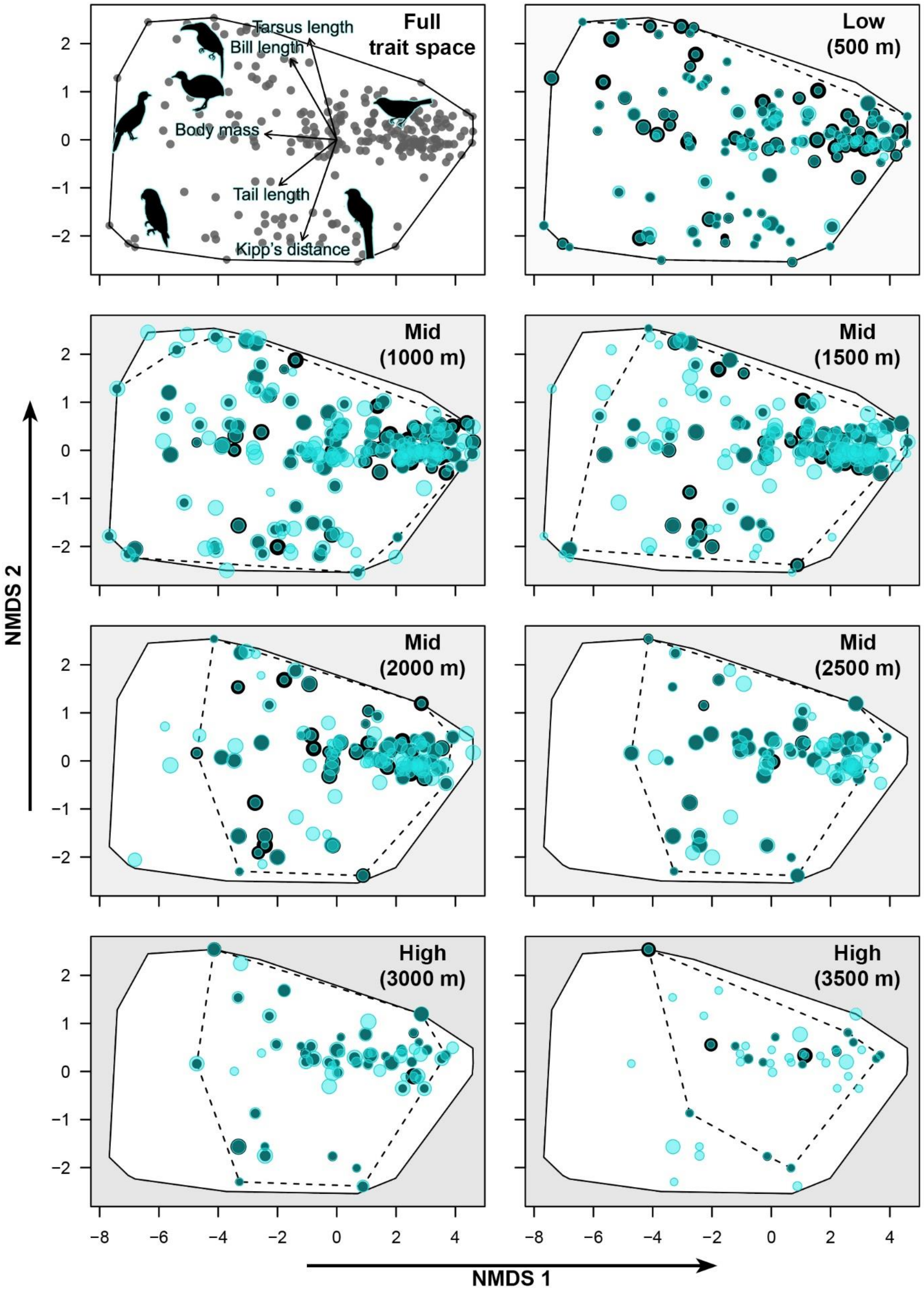

Figure S4. Projected changes in functional trait spaces of frugivorous bird assemblages under climate change assuming a *range expansion scenario*. The functional trait space is derived

from Nonmetric Multidimensional Scaling (NMDS, based on Euclidean distances) which condenses the dissimilarity of the first five PCoA axes, computed from ten morphological bird traits (bill width, bill length, bill height, tail length, tarsus length, tarsus sagittal width, tarsus lateral width, wing length, Kipp's distance and body mass) onto two axes for visualization (NMDS 1, NMDS 2). Trait spaces are shown for the entire bird assemblage (upper left) and for assemblages on each of the seven elevational levels (500 m to 3500 m, at 500 m intervals). Black polygons illustrate the functional trait space of the entire bird assemblage. Black stippled polygons illustrate the functional trait space of the current local assemblage at the given elevation. Each dot represents one bird species (n = 232). Within each elevational level, dot size represents the probability of occurrence as derived from SDMs. Black dots represent current assemblages, transparent green dots represent occurrence probabilities under projected climate change (based on the RCP 8.5 scenario for 2080, MIROC5 climate model). Species-level changes are visible in four ways: (1) complete black dots indicate future loss of species, (2) complete light green dots indicate future immigration of species, (3) dark green dots with a black ring indicate future decline in occurrence probability, and (4) dark green dots with a light green ring indicate future increase in occurrence probability. Bird silhouettes in the upper left panel indicate the approximate location of key taxonomic groups in trait space (counterclockwise from top): toucans, tinamous, guans, parrots, trogons and tanagers. Fitted trait arrows indicate the relationship between five morphological bird traits (representing the different types of traits) and the ordination axes.

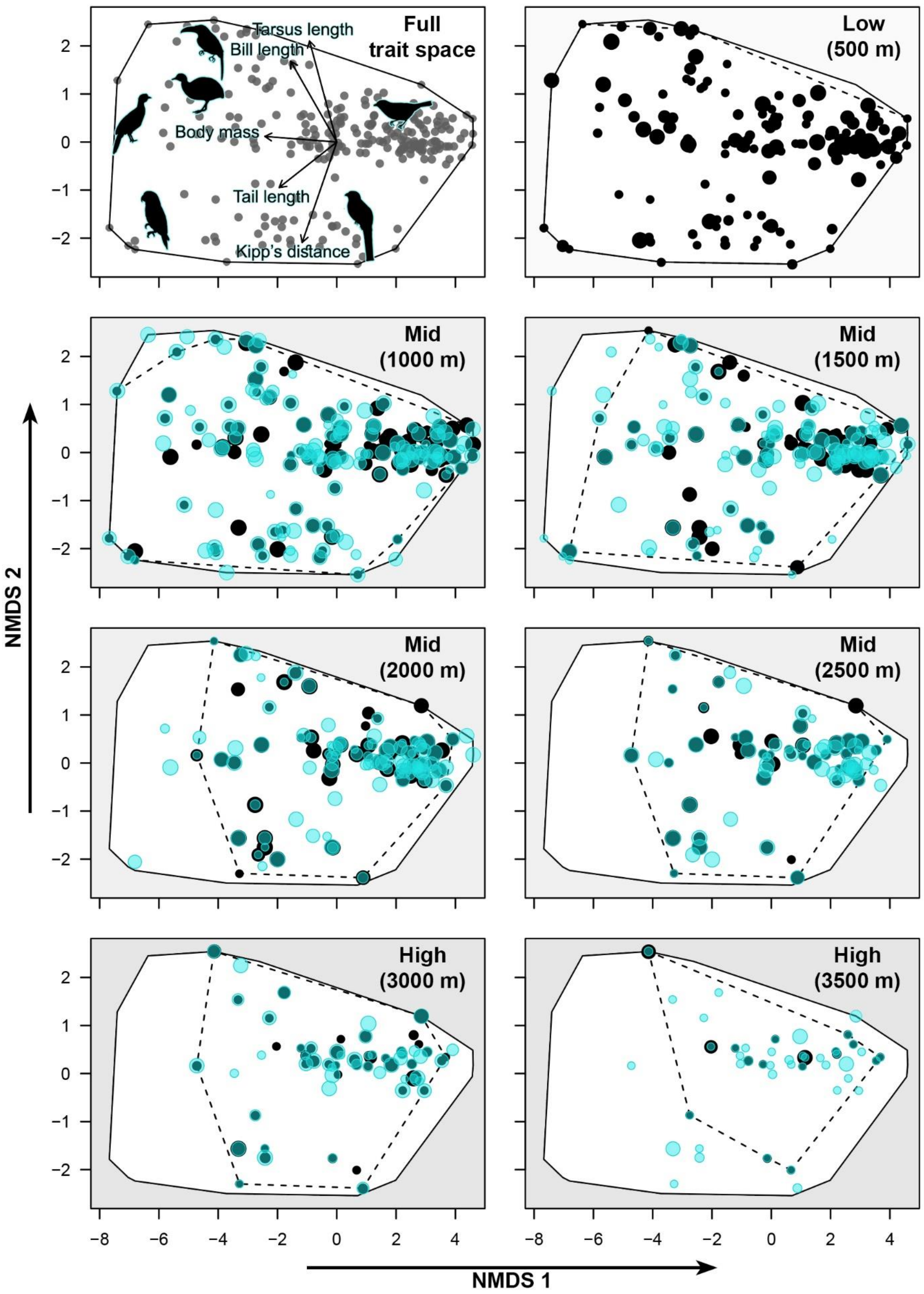

Figure S5. Projected changes in functional trait spaces of frugivorous bird assemblages under climate change assuming a *range shift scenario*. The functional trait space is derived from

Nonmetric Multidimensional Scaling (NMDS, based on Euclidean distances) which condenses the dissimilarity of the first five PCoA axes, computed from ten morphological bird traits (bill width, bill length, bill height, tail length, tarsus length, tarsus sagittal width, tarsus lateral width, wing length, Kipp's distance and body mass) onto two axes for visualization (NMDS 1, NMDS 2). Trait spaces are shown for the entire bird assemblage (upper left) and for assemblages on each of the seven elevational levels (500 m to 3500 m, at 500 m intervals). Black polygons illustrate the functional trait space of the entire bird assemblage. Black stippled polygons illustrate the functional trait space of the current local assemblage at the given elevation. Each dot represents one bird species (n = 232). Within each elevational level, dot size represents the probability of occurrence as derived from SDMs. Black dots represent current assemblages, transparent green dots represent occurrence probabilities under projected climate change (based on the RCP 8.5 scenario for 2080, MIROC5 climate model). Species-level changes are visible in four ways: (1) complete black dots indicate future loss of species, (2) complete light green dots indicate future immigration of species, (3) dark green dots with a black ring indicate future decline in occurrence probability, and (4) dark green dots with a light green ring indicate future increase in occurrence probability. Bird silhouettes in the upper left panel indicate the approximate location of key taxonomic groups in trait space (counterclockwise from top): toucans, tinamous, guans, parrots, trogons and tanagers. Fitted trait arrows indicate the relationship between five morphological bird traits (representing the different types of traits) and the ordination axes.
